# Italian norms and naming latencies for 357 high quality color images

**DOI:** 10.1371/journal.pone.0209524

**Published:** 2019-02-22

**Authors:** Eduardo Navarrete, Giorgio Arcara, Sara Mondini, Barbara Penolazzi

**Affiliations:** 1 Department of Developmental and Social Psychology, Università di Padova, Padova, Italy; 2 IRCCS Fondazione Ospedale San Camillo, Venezia, Italy; 3 Department of General Psychology, Università di Padova, Padova, Italy; 4 Human Inspired Technologies Research Centre-HIT, Padova, Italy; 5 Department of Life Sciences, Università di Trieste, Trieste, Italy; Italian National Research Council, ITALY

## Abstract

In the domain of cognitive studies on the lexico-semantic representational system, one of the most important means of ensuring effective experimental designs is using ecological stimulus sets accompanied by normative data on the most relevant variables affecting the processing of their items. In the context of image sets, color photographs are particularly suited to this purpose as they reduce the difficulty of visual decoding processes that may emerge with traditional image sets of line drawings. This is especially so in clinical populations. In this study we provide Italian norms for a set of 357 high quality image-items belonging to 23 semantic subcategories from the Moreno-Martínez and Montoro database. Data from several variables affecting image processing were collected from a sample of 255 Italian-speaking participants: age of acquisition, familiarity, lexical frequency, manipulability, name agreement, typicality and visual complexity. Lexical frequency data were derived from the CoLFIS corpus. Furthermore, we collected data on image oral naming latencies to explore how the variance in these latencies could be explained by these critical variables. Multiple regression analyses on the naming latencies show classical psycholinguistic phenomena, such as the effects of age of acquisition and name agreement. In addition, manipulability was also a significant predictor. The described Italian normative data and naming latencies are available for download as supplementary material.

## Introduction

Object naming is perhaps the most widely exploited task for studying lexical access during speech production. Decades of research using this paradigm have allowed researchers to identify some of the variables that influence the speed and the accuracy with which words are retrieved from the mental lexicon. It is undeniable that this advance in psycholinguistic knowledge of speech production processes has been closely linked to the appearance of standardized sets of stimuli to be named.

In this respect, one of the first and most influential normative data sets is the battery of Snodgrass and Vanderwart [1]. This set consists of 260 black and white line drawings containing values for four relevant variables affecting cognitive processing during object naming: familiarity, image agreement, name agreement and visual complexity (for a color version of the battery see [2]). Normative data studies have recently started to use a more ecologically valid type of stimuli, where line-drawings are being replaced by photographs. Under the assumption that photographs provide more surface and texture details than line-drawings, it has been hypothesized that photographs would accelerate visual processing and that this, in turn, could accelerate the lexicalization process. Congruent with this hypothesis, Salmon, Mateshon and McMullen [3] showed, for instance, that photographs of tools are named faster than their corresponding line-drawings. Salmon and colleagues interpreted these results as congruent with the notion that the automatic activation of the motor cortex areas associated with the use of tools (e.g., [4]) is facilitated by photographic stimuli in comparison to line-drawing stimuli. These results indicate the importance of controlling for visual features of the items to be named, at least for this specific semantic category. At the same time, visual features are likely to play an important role in other semantic categories beyond that of tools [5]. Perceptual characteristics of items also influence other cognitive domains besides word production. For instance, recent memory studies reported that the perceptual characteristics of the items to be learned constitute a cue that impacts memory predictions for those items. Specifically, items that are easier to perceive during encoding generate higher judgments of learning (a.k.a. JOLS), despite the fact that ease of perception does not generally influence subsequent recall performance. Such a phenomenon is observed with word stimuli [6] as well as with picture stimuli [7, 8].

In addition, color is an essential attribute of objects and therefore provides a more realistic representation. The greater richness provided by color photographs compared to black and white photographs has been shown to improve object perception (e.g., [9, 10]), although it does not seem to ameliorate semantic processing [11]. Consequently, normative studies have started to use more ecological color photographs instead of black and white stimuli (e.g., [12–14]).

Normative data studies have not only been concerned about the representation modality of the stimuli (black and white line-drawings or color photographs), but also about the number of critical variables included in these studies as predictors of lexical access during speech production. Apart from the four variables presented in the original study by Snodgrass and Vanderwart [1], compelling evidence shows that other variables affect the lexicalization process, like, for instance, the age at which a word is first learned. Specifically, early acquired words tend to be named faster and more accurately than late acquired words. This phenomenon, known as the age of acquisition (AoA) effect, is not exclusive to object naming but is widespread across several lexico-semantic processes such as semantic categorization [15], reading [16], or the probability of retrieving a word from the mental lexicon during language production [17] (for a review, see [18]). Another variable determining the speed and accuracy of word retrieval is word frequency. In object naming tasks, high frequency words are named faster and more accurately than low frequency words [19, 20]. Critically, such an advantage is absent when the task does not require lexical access, as for instance when participants are asked to indicate whether previously presented words denoting the objects depicted in target pictures (i.e., old/new decision task; [21]). This evidence suggests that the phenomenon is mainly ascribed to a lexico-phonological level of processing [22]. However, it is debatable whether word frequency is still a reliable predictor of naming latencies once AoA is taken into account [23, 24]. Indeed, a recent Bayesian meta-analysis indicates that the influence of word frequency in picture naming latencies is less relevant than traditionally thought [25]. A third critical predictor for object naming is manipulability. Broadly speaking, manipulability refers to the possibility of manually interacting with a specific object. It has been recently reported that items with a high level of manipulability are named faster than items with a low level of manipulability [3, 26]. Although this variable remains vague (see for discussion, [13]) and it is still unclear at which level of processing the advantage takes place (see for discussion, [26]), the phenomenon has been replicated in different languages.

In sum, since the original study of Snodgrass and Vanderwart [1] researchers have focused on more ecological stimuli such as color photographs and, at the same time, have discovered a number of standardized variables crucially affecting performance in lexical tasks. The objective of the present study is to offer researchers working with Italian-speaking participants a standardized set of 357 high quality color photographs ascribable to a high number of subcategories together with norms for eight variables affecting image processing: AoA, familiarity, lexical frequency, manipulability, two name agreement measures (see below), typicality and visual complexity. To this end, we standardized in the Italian language the set of images provided by Moreno-Martínez and Montoro [27]. In addition to the cross-linguistic validation, we conducted an oral naming study in order to identify the more relevant predictors of naming latencies for the set of images.

## Method

### Participants

A total of 255 healthy Italian native speakers (198 females, 57 males; mean age: 21.29; sd: ± 3.54; 238 right-handed, 17 left-handed) participated in the rating study. In addition, twenty Italian native speakers (15 females, 5 males; mean age: 20.6; sd: ± 1.39; 19 right-handed, 1 left-handed) took part in the oral naming study. All 275 participants provided their written consent, had normal or corrected-to-normal vision and were all students at Padova University, who attended a degree course in Psychology and participated to obtain university credits.

#### Ethical statement

The procedures were approved by the Ethical Committee for Psychological Research of the University of Padova before the study began (Protocol number: 1395-CB7EFAF01EE7652929D155AFEE6552FF; Title: *Mechanisms of Word Retrieval in Spoken Language Production*). Additionally, participation was voluntary and participants were advised that they were free to suspend their participation in the experiment at any time and for any reason.

### Stimuli

**Fig 1:**
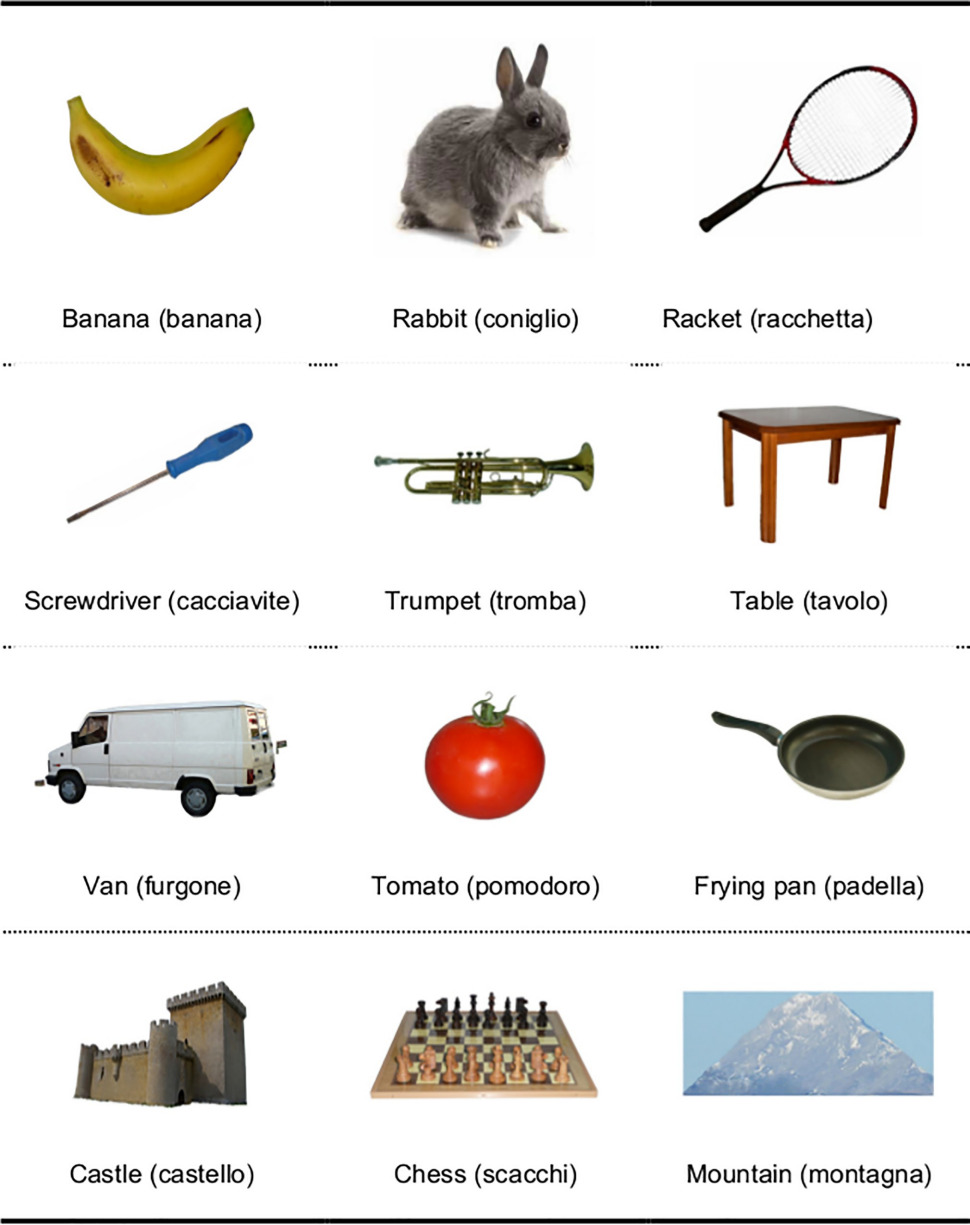
Some examples of the set of color photographs. Italian names are given in brackets. Reprinted from Moreno-Martínez and Montoro. “An ecological alternative to Snodgrass & Vanderwart: 360 high quality colour images with norms for seven psycholinguistic variables”. PLOS ONE, 2012 May 25;7(5): e37527 under a CC BY license, with permission from PLOS ONE, original copyright 2012.

The stimuli were the freely available set of color photographs by Moreno-Martínez and Montoro [27]. This set is composed of 360 high-quality color photographs belonging to 23 semantic subcategories. Specifically, ten subcategories were selected from the living domain: animals, birds, body parts, dried fruits, insects, flowers, fruits, sea creatures, trees, vegetables. Twelve subcategories were selected from the nonliving domain: buildings, clothing, foodstuff, furniture, jewelry, kitchen utensils, musical instruments, office material, sports/games, tools, vehicles, weapons; and finally, nonliving natural things (e.g., containing items like ‘mountain’, ‘stone’, etc.). Because three items of the original database (i.e., “zarajo” and “porra” from the foodstuff category and “churrera” from kitchen utensils) were typical items of the Spanish culture and were impossible to translate into Italian, they were not included in the Italian set of images. Therefore, we collected norms for a total of 357 items. As described by Moreno-Martínez and Montoro [27], the color photographs were taken by the authors, the images were then modified to remove their original backgrounds (except for the nonliving natural things) and placed on a plain white background. Images have a mean dimension of 265x223 pixels and, for each category susceptible to being oriented, half of the items were left-facing and the other half were right-facing. Some examples of items are presented in Fig 1. Italian lexical frequency values were retrieved from the CoLFIS database (which comprises 3,798,275 lexical occurrences; [28]).

### Procedure–rating tasks

To guarantee uniformity across studies, the experimental procedure was kept as similar as possible to that used in Moreno-Martínez and Montoro’s study [27]. The images were shown to a sample of 255 participants. To guarantee high consistency with the original study, the 357 images to be evaluated were divided into three lists (A, B, C) containing a similar number of exemplars from each of the 23 subcategories and totaling 119 items each. Participants were randomly assigned to perform the task on one of the three lists into which the entire image set was divided. Specifically, 81 participants were assigned to list A (64 females and 17 males; age: 21.46±2.61), 90 participants were assigned to list B (69 females and 21 males; age: 21.47±5.24) and 84 were assigned to list C (65 females and 19 males; age: 20.94±1.84). Without any time pressure, they had to typewrite on the computer keyboard the name of the object represented in each figure. Subsequently, they were required to rate the five psycholinguistic variables included in the study: visual complexity, AoA, familiarity, manipulability and typicality. Visual complexity was always the first rating assessed, and typicality was always the last. The presentation of the other three rating tasks (AoA, familiarity, manipulability) was randomly presented across subjects. Participants performed the task individually in one session lasting about 90 minutes (with self-administered rest periods), and were tested simultaneously in groups of about 30 people in the same room.

The task was preceded by a practice phase in which participants were required to typewrite the name of and subsequently rate 10 items not belonging to the main dataset for the same described variables. This practice phase was aimed at enabling participants to become familiar with the task and to develop anchor points useful for rating the subsequent stimulus material. Delivery of images and participants’ responses was controlled by E-Prime 2.0 software (Psychology Software Tools, Inc., Pittsburgh, PA). Pictures were displayed on computers with Dell monitor 21.52''; participants’ distance from the screen was approximately 60 cm. Each image was preceded by a 500 ms-fixation cross and remained visible for 3000 ms for the naming task or until a response was given for the rating task.

In the *typewriting* (written naming) task participants were asked to type the name of the object represented in each image, trying to select the most precise and specific name rather than general names indicating the category they belonged to (e.g., “rose”, instead of general names like “flower” or “plant”). Participants typed the name of each image on the computer keyboard without time pressure. They were also instructed to type the initials NC for “I don’t know” (NC = “non conosco” in Italian) if they did not recognize the object of the image, to type PL for “tip of the tongue” (PL = “punta della lingua” in Italian) if they knew what the object of the image represented but were momentarily unable to remember its name, and to type NR for “don’t remember” (NR = “non ricordo” in Italian) if they recognized the object of the image but did not know if there was a word to name it. Typed responses were saved by the program. Name agreement was calculated based on the percentage of participants who named the item according to its dominant name. Two measures of name agreement were calculated: the percentage of participants who gave the dominant name to each specific item and the H statistic. The H statistic is a logarithmic function describing the different names that an item received and the proportion of participants giving each name [1] to capture information about the dispersion of the names. It has been shown that the H statistic captures more information about the variability of names across participants than the simple percentage-of-agreement measure [1, 29, 30]. For example, if only one name is given to a photograph, H equals zero; if two names occur with equal frequencies, H equals 1. Thus, H increases with the number of names given for the same item, and it is higher if the alternatives have similar probabilities.

The *rating* tasks were performed by pressing the number on the keyboard that corresponded to the participant’s evaluation. In line with the original study [27], in the visual complexity ratings participants were required to “rate the visual complexity of the image itself, rather than that of the object it represents”, evaluating “the amount of details, intricacy of lines, pattern and quantity of colors presented in the image” using a 5-point scale (1 = very simple, 5 = very complex). For the AoA, familiarity, manipulability and typicality ratings, the image was presented together with the name of the item (i.e., the expected name). Additionally, in the typicality rating task, the category name of the item was also provided (e.g., “fruit”, for the item “lemon”).

For AoA participants were instructed to rate the age at which they thought they had first learned each word using a seven-point scale (1 = 0–2 years, 7 = 13 years or more). In the familiarity rating task, they were instructed to rate each item by assessing how often they thought they had come across each of them and how frequently they came into contact with the concept (both directly through real-life exemplars and in a mediated way, as represented in the media), using a 5-point scale (1 = very unfamiliar, 5 = very familiar). In the manipulability rating task, participants were instructed to rate each item by assessing “the degree to which using a human hand is necessary for this object to perform its function”, using a 5-point scale (1 = never necessary, 5 = totally indispensable). The typicality test aims at measuring the degree at which a concept is a representative exemplar of its category. Participants scored how representative of its category they thought an exemplar was (e.g., “ship” for “vehicle”) using a 5-point scale (1 = not at all prototypical, 5 = very prototypical).

### Procedure–oral naming task

In the oral naming task, participants were asked to name the photos as fast and accurately as possible. They were instructed to remain silent in case they did not recognize or did not know the name of the object. The pictures were displayed on a computer Dell OptiPlex 520, Pentium 4 3.0 GHz, with Dell monitor 21.52''. Participants were seated approximately 50 cm from the computer screen, wearing a headset microphone (BeeBang—BNG806UC—USB Headset Headphone). Naming latencies were measured from the appearance of the stimulus. An experimental trial contained the following: (1) a fixation cross in the center of the screen for 500 ms; (2) a blank screen for 500 ms; (3) and the target stimulus for 3000 ms or until the participant responded. The next trial began 1500 ms after the onset of participants’ response or the disappearance of the stimulus. Stimulus presentation and response recording were controlled by DMDX program [31]. The set of items was presented in a different random order for each participant. Due to a mistake during the randomization of the lists, one item (i.e., Square.jpg) was not presented. There was a short pause after 50 trials. The first two trials at the beginning of the experiment and after each pause were warm-up trials containing 2 filler images not presented in the experimental set. Naming latencies and accuracy were determined offline using the CheckVocal software [32].

## Results–normative study

Table 1 reports summary statistics for all the variables, and Table 2 the same statistics separately for all the subcategories. Table 3 shows Pearson’s correlations among the variables. Table in S1 Table provides descriptive statistics (means and standard deviations) for each variable assessed in the rating tasks (AoA, familiarity, manipulability, typicality, visual complexity) for all of the dataset stimuli (grouped by semantic category). In line with the original Spanish dataset, the word-form frequency of the dominant name is expressed as a natural logarithm. Two measures of name agreement are provided in S1 Table. In table in S2 Table we report the alternative names each item received; while in table in S3 Table we report indexes of individual item analysis, including an item difficulty measure and two indexes of item discrimination based on item-test correlations (biserial and point-biserial) [33, 34].

**Table 1:** Summary statistics for all the variables.

	Agr-P	Agr-H	Freq	AoA	Vis Com	Fam	Man	Typ
mean	0.56	1.49	7.61	3.99	2.61	3.10	2.84	3.48
sd	0.35	1.01	2.18	1.36	0.71	0.99	1.26	0.83
median	0.64	1.41	7.73	3.89	2.52	3.03	2.95	3.56
max	1.00	3.51	13.23	6.83	4.43	4.93	4.96	4.85
min	0.00	0.00	0.00	1.42	1.17	1.02	1.03	1.44
range	1.00	3.51	13.23	5.41	3.26	3.91	3.93	3.41
kurtosis	-1.44	-1.24	1.07	-0.94	-0.61	-1.00	-1.43	-0.70
skewness	-0.28	0.14	-0.51	0.14	0.25	0.12	0.07	-0.37
Q1	0.23	0.58	6.39	2.99	2.04	2.33	1.56	2.90
Q3	0.90	2.39	8.98	5.18	3.12	3.93	3.92	4.16
Mode	0.00	0.00	7.02	5.63	1.82	2.60	1.15	4.16

Note. Agr-P = Percentage of name agreement; Agr-H = H statistic on name agreement; Freq = Lexical frequency (natural logarithm); AoA = Age of Acquisition; Vis Com = Visual Complexity; Fam = Familiarity; Man = Manipulability; Typ = Typicality.

**Table 2:** Summary statistics for all the variables for each category.

	Agr-P		Agr-H		Freq		AoA		Vis Com		Fam		Man		Tip	
Category	M	SD	M	SD	M	SD	M	SD	M	SD	M	SD	M	SD	M	SD
Clothes	0.75	3.98	0.27	0.49	0.93	0.62	3.17	0.97	8.36	1.02	1.07	3.61	1.39	1.94	1.01	3.35
Tress	0.20	2.65	0.30	0.33	0.72	0.23	4.93	0.66	7.47	0.49	2.10	3.62	0.84	2.81	0.79	1.45
Food	0.37	3.18	0.28	0.58	0.71	0.52	4.42	0.95	6.58	0.71	2.29	3.08	1.47	2.51	1.99	3.42
Animals	0.79	2.56	0.25	0.27	1.02	0.42	3.46	0.73	7.34	0.81	0.92	3.61	1.25	3.11	1.56	1.39
Weapons	0.61	2.12	0.37	0.73	0.92	0.67	4.44	0.35	8.35	0.92	1.21	3.17	1.10	2.56	1.34	4.11
Tools	0.39	2.53	0.37	0.39	0.97	0.42	4.82	0.58	7.18	0.65	1.88	3.42	1.10	2.06	1.72	4.33
Desk Material	0.63	3.74	0.35	0.59	0.96	0.30	3.95	0.90	7.41	0.86	1.39	3.69	1.14	2.01	3.27	4.54
Marine Creatures	0.44	2.13	0.37	0.57	0.94	0.60	4.82	0.73	6.10	0.89	1.68	3.08	1.43	2.73	2.36	1.63
Buildings	0.54	2.79	0.32	0.68	0.89	0.36	4.33	0.89	9.10	1.02	1.83	2.91	1.36	3.41	2.09	1.99
Flowers	0.46	2.95	0.40	0.51	1.32	0.19	4.56	0.82	6.43	0.66	1.97	3.82	1.35	2.62	2.08	1.82
Fruits	0.56	3.46	0.42	0.42	1.09	0.31	3.64	1.02	7.09	0.91	1.28	3.62	1.57	1.89	2.54	3.39
Nuts	0.45	3.06	0.32	0.32	0.82	0.56	4.17	0.68	6.89	0.80	1.91	3.46	0.67	2.06	1.47	3.09
Jewelry	0.49	3.16	0.33	0.60	0.89	0.70	4.17	1.09	7.17	0.99	1.63	3.55	1.23	2.83	2.67	3.51
Insects	0.61	2.95	0.30	0.53	0.88	0.16	3.79	0.87	6.63	0.58	1.49	3.75	1.28	2.97	1.50	1.33
Furniture	0.67	4.10	0.37	0.49	1.25	0.66	3.39	0.83	8.36	0.88	1.21	3.72	1.22	2.48	2.01	2.84
Nature	0.62	3.47	0.34	0.71	1.03	0.44	3.08	0.88	9.90	0.89	1.40	3.34	1.06	2.67	1.64	1.44
Body parts	0.64	3.98	0.34	0.51	1.01	0.94	3.06	0.98	9.86	0.77	1.09	3.80	1.68	2.37	1.52	2.09
Sports/Games	0.57	3.10	0.34	0.76	0.94	0.94	3.55	0.63	6.76	0.71	1.32	3.39	1.21	2.82	2.27	3.93
Musical instruments	0.64	2.66	0.31	0.67	0.79	0.21	4.48	0.64	7.83	0.77	1.36	3.75	1.08	3.29	1.65	4.71
Birds	0.55	2.52	0.34	0.46	0.93	0.19	4.31	0.80	7.03	0.60	1.66	3.17	1.40	3.30	1.02	1.30
Kitchen utensils	0.44	3.59	0.39	0.61	1.22	0.26	4.39	1.07	6.14	0.93	1.78	3.60	1.53	2.22	3.07	4.31
Vehicles	0.62	3.48	0.34	0.58	0.85	0.77	3.48	0.81	9.16	1.10	1.30	3.25	0.88	3.11	1.91	3.61
Vegetables	0.60	3.42	0.39	0.47	1.08	0.27	4.15	0.84	7.35	0.62	1.38	3.57	1.14	2.08	1.36	3.30

Note. Agr-P = Percentage of name agreement; Agr-H = H statistic on name agreement; Freq = Lexical frequency (natural logarithm); AoA = Age of Acquisition; Vis Com = Visual Complexity; Fam = Familiarity; Man = Manipulability; Typ = Typicality.

**Table 3:** Correlation matrix between variables.

	Agr-P	Agr-H	Freq	AoA	Vis Com	Fam	Man	Tip
Agr		-0.82**	0.45**	-0.71**	-0.20**	0.56**	0.04	0.49**
H			-0.34**	0.62**	0.24**	-0.47**	-0.05	-0.42**
Freq				-0.59**	-0.06	0.52**	-0.003	0.42**
AoA					0.29**	-0.81**	-0.02	-0.65**
Vis Com						-0.38**	-0.26**	-0.21**
Fam							0.21**	0.71**
Man								0.11*
Tip								

Note. Agr-P = Percentage of name agreement; Agr-H = H statistic on name agreement; Freq = Lexical frequency (natural logarithm); AoA = Age of Acquisition; Vis Com = Visual Complexity; Fam = Familiarity; Man = Manipulability; Typ = Typicality.

*p < .05

**p < .01.

### Reliability

To determine the reliability of our data, we correlated the variables among items sharing the same dominant name in the present study and other studies in the literature [27]. In particular, 357 items overlapped with Moreno-Martínez and Montoro [27], 50 with Adlington, Laws and Gale [35], 69 with Brodeur et al. [12], 113 with Moreno-Martínez, Montoro and Laws [36], 107 with Snodgrass & Vanderwart [1], 81 with the English version of Viggiano et al. [14], and 80 with the Italian version of Viggiano et al. [14]. Pearson’s correlations are reported in Table 4. Correlations fluctuated between .28 and .98.

**Table 4:** Correlations between current stimuli and those of Moreno-Martínez and Montoro (2012), Adlington et al. (2009), Brodeur et al. (2010), Moreno- Martínez et al. (2011), Snodgrass & Vanderwart (1980) and Viggiano et al. (2004).

	Items (n)	Agr-P	Freq	AoA	Vis Com	Fam	Man
Moreno-Martínez & Montoro	357	0.65	0.57	0.87	0.94	0.88	0.96
Adlington et al.’s	50	0.53	0.77	0.85	0.64	0.76	NA
Brodeur et al.’s	69	0.38	NA	NA	0.66	0.65	0.52
Moreno-Martinez et al	113	0.75	0.62	0.9	0.88	0.87	0.98
Snodgrass & Vanderwart	107	0.36	0.58	0.79	0.75	0.85	NA
Viggiano et al. (English)	81	0.28	NA	NA	0.79	0.74	NA
Viggiano et al. (Italian)	80	0.31	NA	NA	0.84	0.82	NA

Note. Agr-P = Percentage of name agreement; Agr-H = H statistic on name agreement; Freq = Lexical frequency (natural logarithm); AoA = Age of Acquisition; Vis Com = Visual Complexity; Fam = Familiarity; Man = Manipulability; Typ = Typicality.

## Results–oral naming study

All answers classified as incorrect names (e.g., semantic superordinates such as “tool” instead of “pliers”; semantic coordinates such as “boat” instead of “ship”), missing responses and verbal dysfluencies were excluded from the analysis. Following those criteria, a total of 30.2% of the data were excluded (15.2% of incorrect responses; 15% of no-responses and 0.03% of voice key problems). Fourteen items did not elicit correct responses (i.e., all responses were incorrect, missing responses or verbal dysfluencies) and were excluded from the analyses. Correct responses have a mean latency of 1203 ms with a standard deviation of 361 ms. Preliminary analyses were performed to control whether phonemic properties of the word-initial phonemes affected voice key activation differently, influencing response latencies [37]. Following previous research in Italian [38], we divided the items into five phonetic categories. ANOVA results did not show any significant effect of the word-initial phoneme on naming latencies (p = .255). Three different kinds of analysis were performed on naming latencies.

In a first descriptive level of analysis (Analysis Type-1), we performed correlations between naming latencies and the eight variables of the normative study.

In a second type of analysis (Analysis Type-2), we assessed how much of the variance in the naming latencies was explained by each of the above variables. Analysis was performed on the average of the naming latencies of each stimulus. Given the high level of correlation among some of the variables, we adopted the following approach in order to avoid problems of collinearity. In a first step, we assessed the correlations among the variables through a hierarchical clustering analysis using the *varclus* function of the “Hmisc*”* package [39] with the R statistical software [40]. This allowed us to identify clusters of variables (i.e., variables with a Spearman similarity coefficient > .35). This kind of analysis separates variables into clusters that can be scored as a single variable, thus resulting in data reduction. In a second step, in order to select the more important variable within each cluster, we performed likelihood ratio tests among those models containing separately each of the identified variables in each cluster. For model comparison we took into consideration the Bayesian information criterion (BIC) [41] using the *compareLM* function of the “rcompanion” package [42] with R. Once the most important variable for each cluster was selected, in a third and final step, we conducted a multiple regression analysis in order to explore how much of the variance in the naming latencies was explained by the selected variables. This second kind of analysis was performed on a subset of items which, on the normative typewriting task, received a name agreement value equal to or above 50% (i.e., items that elicited the expected name from at least half of participants). This criterion was selected in order to exclude spurious influences, as, for instance, poor visual structural descriptions of the photos, or the impact of idiosyncratic linguistic characteristics of the target words in Italian ([43]; for an example of the influence of name agreement on picture naming latencies see [44]). Following this criterion, the analysis was performed on 196 items (see S1 Text for a further multiple regression analysis including all the variables).

In a third type of analysis (Analysis Type-3), the influence of the variables was explored in those experimental trials in which participants used the expected name to denominate the photo stimuli. That is, correct alternative responses were not considered in the analysis, as, for instance, responses contained detailed description of the photo (e.g., “Indian elephant” instead of “elephant”) or abbreviations (e.g., “auto” instead of “automobile”, see for a similar procedure [45]). In this manner, we ensured that the analysis was performed on those oral responses that were identical to the name used in the normative study. Collinearity was reduced following steps 1 and 2 of the Analysis-Type 2. Naming latencies were analyzed using mixed effects regression model performed at the single trial level, which allowed us to test the influence of the variables considering both by-participants and by-item variabilities [46]. In addition, this approach allowed us to exclude from the analysis each single response that did not elicit the expected name. Analyses were performed on 3979 data points. As the data were not normally distributed, we use the Box-Cox test [47], using the function boxcox in the package ‘‘MASS” [48] to estimate the most appropriate transformation for the data to reduce skewedness and approximate a normal distribution. The test indicated that the reciprocal transformation was the most appropriate transformation (we used -1000/RT to facilitate reading of the results). Latencies of correct responses were analyzed with linear mixed models (LMM) using the package ‘‘lme4” [49]. Analyses were performed with the R statistical software [40] (see S1 Text for a further mixed effects regression model including all the variables).

### Analysis Type-1

As can be seen in Table 5, naming latencies correlated positively with H statistic, AoA and visual complexity and negatively with name agreement, lexical frequency, familiarity and typicality. No significant correlation was obtained between naming latencies and manipulability.

**Table 5:** Correlations between the average naming latencies and the variables.

	Agr-P	Agr-H	Freq	AoA	Vis Com	Fam	Man	Typ	Df
Set A	-0.56**	0.63**	-0.35**	0.57**	0.14**	-0.45**	0.02	-0.54**	341
Set B	-0.54**	0.62**	-0.32**	0.66**	0.24**	-0.48**	0.01	-0.50**	194

Note. Set A = 343 items; Set B = 196 items (items with agreement values in the normative study equal or above 0.5; see main text for details). See the Note in Table 1. Df = degrees of freedom.

**p < .01

### Analysis Type-2

**Fig 2:**
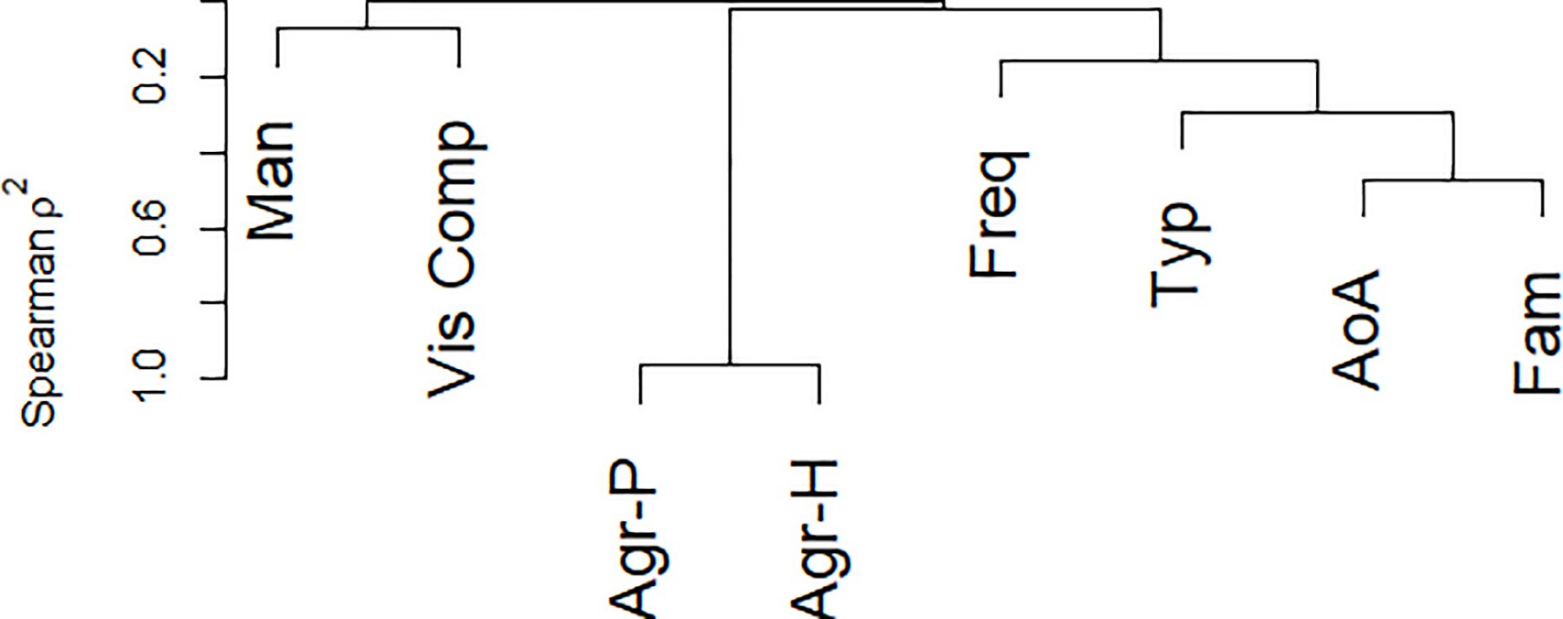
Hierarchical clustering analysis using Spearman’s p^2^ for the eight explored variables. See the Note in Table 1.

**Fig 3:**
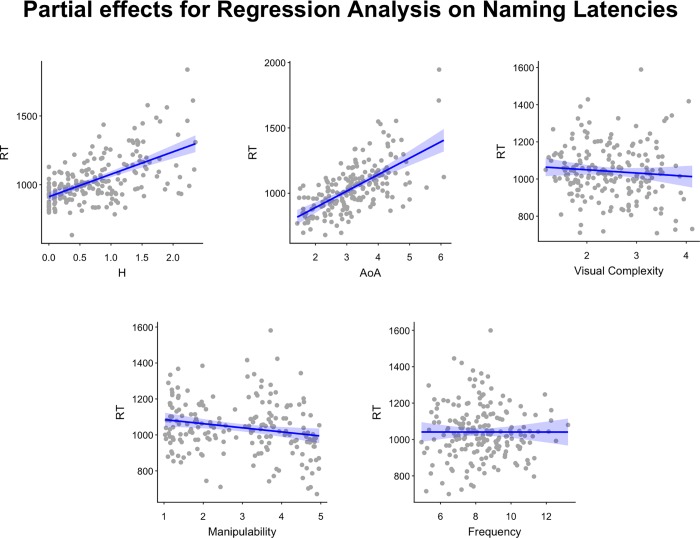
The figure shows the partial effects for the regression model on Naming Latencies. Each panel represents the partial effect of a predictor included in the regression. The blue lines represent the regression for that partial effect. The shaded blue represents the area delimiting the standard error of the estimate. The gray points indicate the partial residuals associated with the predictor.

Fig 2 shows the hierarchical clustering structure among the variables. Two clusters of highly correlated variables emerged. Agreement and H statistic formed a cluster and typicality, AoA and familiarity formed another. In the first cluster, the likelihood ratio test indicated that, compared to agreement, H statistic produced a significant increase in the explained variance (χ^2^ = 27.75, *p* < .001). In the second cluster, the likelihood ratio tests indicated that AoA produced a significant increase in the explained variance compared to typicality (χ^2^ = 58.47, *p* < .001) and familiarity (χ^2^ = 63.41, *p* < .001). Thus, the multiple regression analysis was conducted with five variables: H statistic, AoA, frequency, manipulability and visual complexity. Partial effects of the model are illustrated in Fig 3. As can be seen in Table 6, H statistic, AoA and manipulability were significant predictors of naming latencies (R^2^ = 0.611). Specifically, faster naming latencies were obtained for items with lower H statistic values, acquired early in life and with higher manipulability rating. No effects of visual complexity and lexical frequency were obtained. Tolerance statistics were all above .5 and the average of the variance inflation factor (VIF) was 1.38, suggesting that collinearity was not a problem of the regression model [34].

**Table 6:** Results of the multiple regression analysis, with naming latencies as the criterion variable and H statistic, age of acquisition, visual complexity, manipulability and lexical frequency as predictor variables.

	Estimate	St.Error	t value	Pr (>|t|)	Tolerance	VIF
Intercept	620.952	93.067	6.672	< .001		-
H statistic	163.114	18.523	8.806	< .001	0.82	1.21
Age of acquisition	125.974	14.729	8.552	< .001	0.54	1.84
Visual complexity	-17.335	17.115	-1.013	0.312	0.75	1.32
Manipulability	-22.860	8.740	-2.615	0.009	0.85	1.16
Lexical frequency	-0.064	7.382	-0.009	0.993	0.71	1.39

Notes: VIF = variance inflation factor. Degrees of freedom = 190.

### Analysis Type-3

**Fig 4:**
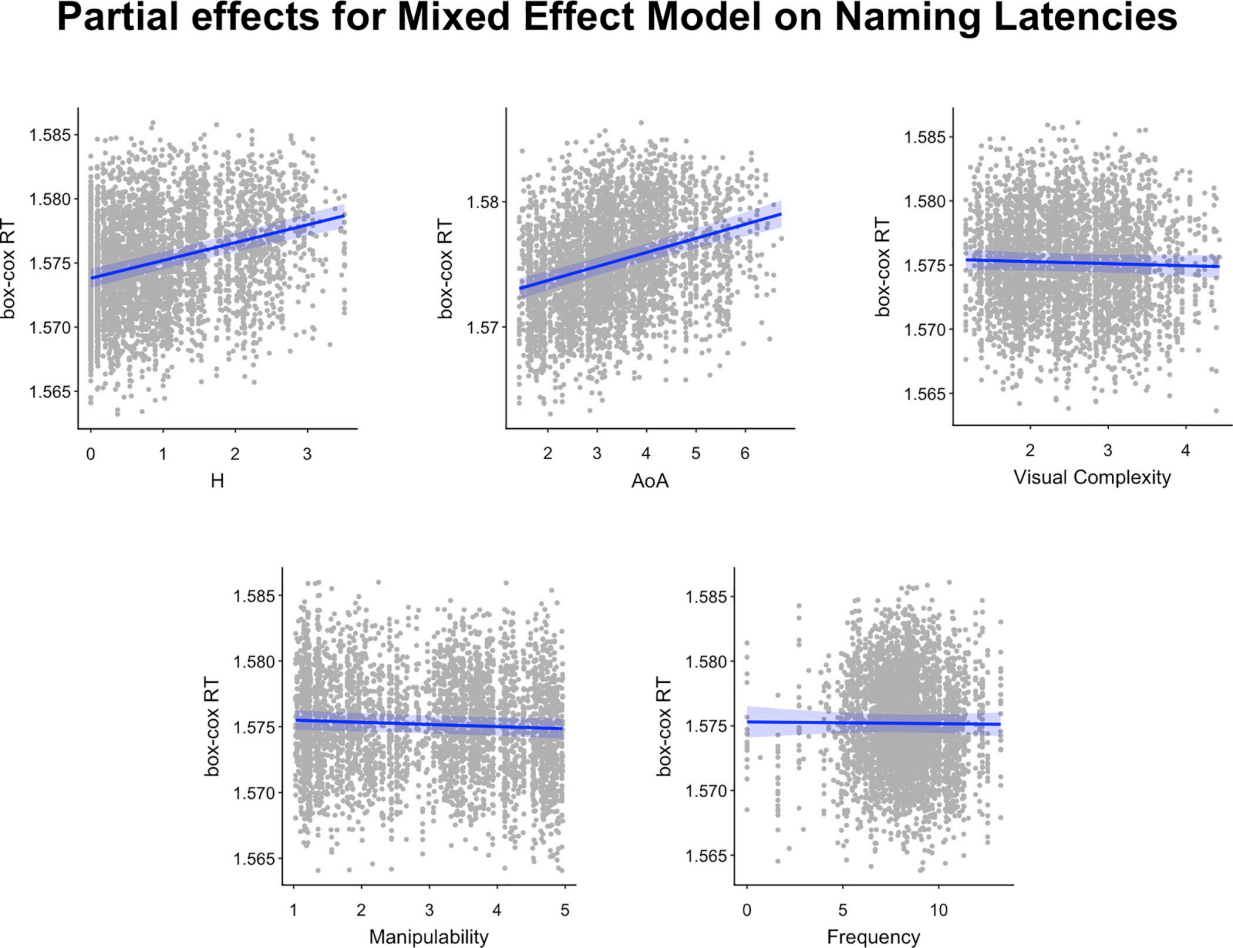
Partial effects of the mixed models on naming latencies. The figure shows the partial effects for the regression model on naming latencies. Each panel represents the partial effect of a predictor included in the regression. The blue line represents the regression line for that partial effect. The shaded blue area represents the area delimiting the standard error of the estimate. Gray points indicate the partial residuals associated with the predictor.

In the mixed effects analysis performed at the single trial level, the model contained random intercepts for participants and items, and the five fixed predictors of the multiple regression used in *Analysis Type-2*. The model was fitted with the following lme4 syntax: *NL bc ~ H + AoA + Visual Complexity + Manipulability + Frequency + (1|Subject) + (1|Item)*, *REML = FALSE*. Where NL_bc indicates Box-Cox transformed Naming Latencies (i.e., Naming Latencies). Diagnostic plots on model results showed a satisfactory fit. Results of the model are reported in Table 7, see also Fig 4.

**Table 7:** Results of Mixed effects Model with naming latencies as the criterion variable and H statistic, age of acquisition, visual complexity, manipulability and lexical frequency as predictor variables.

	Estimate	St.Error	t value	Pr (>|t|)
Intercept	1.571	8.993x10^-4^	1747	< .001
H statistic	1.390x10^-3^	1.326x10^-4^	10.481	< .001
Age of acquisition	1.125x10^-3^	1.262x10^-4^	8.915	< .001
Visual complexity	-1.610x10^-4^	1.525x10^-4^	-1.056	0.291
Manipulability	-1.632x10^-4^	7.999x10^-5^	-2.041	0.042
Lexical frequency	-1.394x10^-5^	6.214x10^-5^	-0.224	0.822

The table reports the results of the mixed effects model, with Box-Cox transformed Naming Latencies as dependent variable. The first column reports the name of each predictor. The second column reports the estimated coefficients (beta) for each predictor. The third column reports the standard error of the estimate. The fourth column reports the t-value associated with the predictors. The last column reports the p-values. The model included also a by-subject adjustment to intercept (SD = 0.001504) and a by-item adjustment to intercept (SD = 0.001405) as random effects.

## Discussion

The present study provides Italian norms for 357 high quality color photographs from the set of Moreno-Martínez and Montoro [27]. Several psycholinguistic variables that have been showed to affect latency and accuracy during object naming are included: agreement, H statistic, word-lexical frequency, age of acquisition, visual complexity, familiarity, manipulability and typicality. As reported in other normative studies (e.g., [50, 51]), the variables are highly correlated. It is noteworthy that the correlation pattern we obtained matches to a large extent the one reported in the original Moreno-Martínez and Montoro’s study in Spanish.

A second aim of this study, which is the most original part of this study on the Italian validation of Moreno-Martínez and Montoro’s database, was to explore how much of the variance in the latencies of the oral naming task could be explained by the above-mentioned crucial variables. A first descriptive analysis showed that all the variables, except manipulability, correlated with naming latencies. In a second analysis, in order to reduce multicollinearity problems, we separated the variables into clusters through a hierarchical cluster analysis and then we selected the most relevant variable for each cluster. The regression analysis using the five remaining variables as predictors showed a significant effect of H statistic, AoA and manipulability. In particular, the items that tended to elicit a similar name from participants in the typewriting naming normative study (i.e., lower H index) were named faster (e.g., [44]). At the same time, items acquired early in life were named faster than items acquired late in life, replicating the well-known effect of age of acquisition [18]. In addition, manipulability also modulated naming latencies once the control variables of name agreement (i.e., H statistic), age of acquisition and visual complexity were taken into account. Specifically, items that were ranked with high manipulability in the rating study were named faster in the oral naming task. This result suggests that manipulability is a critical variable affecting speech production in picture naming, replicating recent findings [13, 26, 52].

An apparently unexpected outcome is the lack of a significant effect of lexical frequency, since traditionally this variable has been demonstrated to be a very reliable predictor of naming latencies [19, 22]. In order to exclude the possibility that the lack of frequency effect might be due to the specific properties of the Italian database corpus we used (i.e., CoLFIS), further analyses were performed with Worldlex, a more up-to-date corpus [53]. The Worldlex corpus provides three different frequency measures based on Twitter posts, internet blogs and newspapers. Within the 357 items of our experimental set, Worldlex frequency measures were highly correlated with the frequency measures provided by the COLFIS corpus (0.89, 0.89 and 0.91, respectively). Three new multiple regressions with Twitter, blogs and newspapers Worldlex measures were performed. Again, no significant frequency effects were reported (*p* > .14). On the other hand, the lack of frequency effect appears congruent with recent findings in naming tasks which showed no lexical frequency effects when AoA was also included in the statistical analysis as a predictor. This suggests that AoA is a more reliable predictor of naming latencies and that it assimilates part of the effect tied to frequency [23, 24, 54], for a different approach see also [17, 55]. In line with that, when in our analysis the variable AoA is excluded from the multiple regression model the effect of the frequency turns out to be significant (t = -4.375, *p* < .001), with faster naming latencies for more frequent words, for further discussion see [37, 56, 57]. Furthermore, the pattern of results we obtained in the naming task matches the main conclusions of a recent Bayesian meta-analysis [25]. In this meta-analysis, AoA and name agreement measures have a strong influence on naming latencies, while the influence of lexical frequency is unclear and visual complexity yields null effects.

Other studies have provided psycholinguistic indexes in Italian (e.g., [14, 51]). For instance, Duñabeitia and colleagues provided a normative study with name agreement and visual complexity data for 750 color drawings in six different European Languages, including Italian [58]. However, to our knowledge, our study is the first to provide eight psycholinguistic indexes in Italian for such a high number of very ecological items (i.e., 357 quality color photographs). Examining all these variables in detail is of critical relevance in object naming research, as well as in other cognitive research domains, such as memory or object perception. Having well-controlled and ecological stimuli sets is just as important in clinical and neuropsychological domains [59], both to improve assessment procedures and to disclose which processing level can be the most impaired in patients’ failures [11]. This normative study could help item selection for the design of experimental work and clinical trials.

## Supporting information

S1 Table Normative psycholinguistic ratings for each item. (DOCX)

S2 Table Alternative names and responses. (DOCX)

S3 Table Indexes of individual item analysis. (DOCX)

S1 Text Supplemental analysis including all the variables. (DOCX)

S1 Data Data for each item. (CSV)

S2 Data Raw data on expected responses at single trial level. (CSV)
