# Correction: Italian norms and naming latencies for 357 high quality color images

**DOI:** 10.1371/journal.pone.0214561

**Published:** 2019-03-25

**Authors:** The PLOS ONE Staff

There is an error in affiliation 2 for author Giorgio Arcara. The correct affiliation 2 is: IRCCS San Camillo Hospital, Venice, Italy. The publisher apologizes for the error.
