# Supplementary material for: Italian norms and naming latencies for 357 high quality color images
Source: PLoS One. 2019 Feb 22;14(2):e0209524. doi: 10.1371/journal.pone.0209524 (PMC6386297; doi:10.1371/journal.pone.0209524)
Supplement: S1 Table — (DOCX) [file pone.0209524.s001.docx]

|  |  | **Agr** | | **Freq** | **AoA** | | **Vis Com** | | **Fam** | | **Man** | | **Tip** | |
| --- | --- | --- | --- | --- | --- | --- | --- | --- | --- | --- | --- | --- | --- | --- |
| **Englih** | **Italian** | **p** | **H** | **M** | **M** | **SD** | **M** | **SD** | **M** | **SD** | **M** | **SD** | **M** | **SD** |
| **Animals** |  |  |  |  |  |  |  |  |  |  |  |  |  |  |
| Armadillo | armadillo | 0.36 | 2.76 | 5.03 | 5.96 | 1.18 | 3.51 | 1.06 | 1.48 | 0.95 | 1.19 | 0.51 | 2.31 | 1.29 |
| Bat | pipistrello | 0.99 | 0.09 | 7.33 | 3.58 | 1.29 | 3.22 | 1.14 | 2.79 | 1.41 | 1.21 | 0.47 | 3.14 | 1.28 |
| Cat | gatto | 0.96 | 0.28 | 10.01 | 1.70 | 0.95 | 3.01 | 1.20 | 4.48 | 0.91 | 2.20 | 1.19 | 4.82 | 0.47 |
| Cheetah | ghepardo | 0.40 | 2.84 | 7.14 | 4.05 | 1.38 | 3.27 | 1.06 | 2.19 | 1.17 | 1.15 | 0.48 | 3.60 | 1.15 |
| Cow | mucca | 0.94 | 0.37 | 9.17 | 2.05 | 0.91 | 2.84 | 1.10 | 3.64 | 1.31 | 2.16 | 1.19 | 4.58 | 0.78 |
| Crocodile | coccodrillo | 0.88 | 0.53 | 7.77 | 2.95 | 1.34 | 3.27 | 1.08 | 2.43 | 1.42 | 1.15 | 0.57 | 3.38 | 1.15 |
| Dromedary | dromedario | 0.72 | 0.93 | 5.45 | 4.45 | 1.45 | 2.89 | 0.94 | 2.05 | 1.15 | 1.43 | 0.77 | 3.12 | 1.21 |
| Elephant | elefante | 0.99 | 0.09 | 8.40 | 2.46 | 0.93 | 3.00 | 1.22 | 2.80 | 1.45 | 1.38 | 0.67 | 4.41 | 0.86 |
| Giraffe | giraffa | 1.00 | 0.00 | 6.67 | 2.65 | 1.24 | 3.09 | 1.19 | 2.78 | 1.47 | 1.15 | 0.36 | 3.88 | 1.02 |
| Hippopotamus | ippopotamo | 0.87 | 0.74 | 6.43 | 3.03 | 1.37 | 2.69 | 0.90 | 2.25 | 1.24 | 1.16 | 0.47 | 3.56 | 1.14 |
| Horse | cavallo | 0.99 | 0.09 | 10.78 | 2.25 | 0.94 | 3.31 | 1.30 | 3.51 | 1.34 | 2.66 | 1.15 | 4.67 | 0.66 |
| Kangaroo | canguro | 1.00 | 0.00 | 6.79 | 3.40 | 1.36 | 2.89 | 1.01 | 2.48 | 1.35 | 1.23 | 0.57 | 3.46 | 1.12 |
| Lioness | leonessa | 0.60 | 1.36 | 6.42 | 3.05 | 1.27 | 3.08 | 1.09 | 2.44 | 1.37 | 1.20 | 0.52 | 4.21 | 0.90 |
| Lynx | lince | 0.48 | 2.59 | 6.18 | 5.21 | 1.49 | 3.69 | 1.07 | 1.81 | 1.01 | 1.25 | 0.49 | 3.05 | 1.41 |
| Platypus | ornitorinco | 0.44 | 2.15 | 5.49 | 5.84 | 1.33 | 3.17 | 1.11 | 1.63 | 1.04 | 1.13 | 0.34 | 2.06 | 1.27 |
| Rhino | rinoceronte | 0.96 | 0.31 | 6.50 | 3.21 | 1.30 | 3.25 | 1.16 | 2.24 | 1.36 | 1.12 | 0.40 | 3.69 | 1.14 |
| Snake | serpente | 0.89 | 0.71 | 9.04 | 2.85 | 1.19 | 2.49 | 1.11 | 2.74 | 1.44 | 1.36 | 0.74 | 3.80 | 1.15 |
| Tapir | tapiro | 0.28 | 2.65 | 6.09 | 5.59 | 1.42 | 2.95 | 0.95 | 1.51 | 0.82 | 1.11 | 0.31 | 2.00 | 1.28 |
| Tiger | tigre | 0.97 | 0.20 | 8.67 | 2.57 | 1.07 | 3.40 | 1.08 | 2.60 | 1.52 | 1.21 | 0.62 | 4.48 | 0.81 |
| Turtle | tartaruga | 0.92 | 0.56 | 8.34 | 2.89 | 1.19 | 3.09 | 1.25 | 3.25 | 1.36 | 1.56 | 0.84 | 3.99 | 1.03 |
| Zebra | zebra | 0.99 | 0.10 | 6.52 | 3.01 | 1.36 | 3.21 | 1.20 | 2.65 | 1.43 | 1.16 | 0.40 | 3.71 | 1.12 |
| **Birds** |  |  |  |  |  |  |  |  |  |  |  |  |  |  |
| Barn owl | barbagianni | 0.11 | 1.37 | 5.71 | 5.69 | 1.39 | 3.31 | 1.09 | 1.83 | 1.01 | 1.23 | 0.71 | 2.89 | 1.15 |
| Duck | anatra | 0.64 | 1.73 | 7.66 | 3.27 | 1.28 | 3.56 | 1.17 | 3.26 | 1.30 | 1.36 | 0.67 | 3.42 | 1.12 |
| Goldfinch | cardellino | 0.00 | 2.74 | 5.71 | 6.18 | 1.30 | 3.77 | 1.11 | 1.67 | 0.99 | 1.17 | 0.47 | 2.90 | 1.41 |
| Goose | oca | 0.84 | 0.84 | 8.44 | 2.39 | 1.09 | 2.41 | 1.01 | 2.95 | 1.37 | 1.37 | 0.67 | 3.27 | 1.29 |
| Hen | gallina | 0.91 | 0.53 | 8.48 | 2.39 | 1.11 | 3.18 | 1.16 | 3.66 | 1.43 | 1.91 | 0.95 | 3.06 | 1.38 |
| Hummingbird | colibrì | 0.46 | 2.02 | 5.93 | 5.57 | 1.51 | 4.33 | 0.96 | 1.78 | 0.94 | 1.16 | 0.46 | 3.11 | 1.41 |
| Kiwi | kiwi | 0.13 | 2.44 | 7.28 | 6.57 | 0.99 | 3.25 | 1.04 | 1.35 | 0.91 | 1.16 | 0.52 | 1.84 | 1.20 |
| Magpie | gazza | 0.15 | 3.21 | 6.95 | 5.22 | 1.53 | 3.34 | 0.92 | 2.29 | 1.18 | 1.26 | 0.58 | 3.55 | 1.35 |
| Ostrich | struzzo | 0.87 | 0.98 | 7.41 | 3.99 | 1.54 | 3.38 | 1.04 | 2.27 | 1.17 | 1.22 | 0.47 | 2.51 | 1.21 |
| Owl | gufo | 0.84 | 0.83 | 7.22 | 3.31 | 1.24 | 3.32 | 1.02 | 2.52 | 1.14 | 1.20 | 0.59 | 3.55 | 1.09 |
| Partridge | pernice | 0.01 | 2.88 | 6.73 | 6.45 | 1.12 | 3.60 | 0.94 | 1.52 | 0.87 | 1.35 | 0.77 | 2.72 | 1.41 |
| Pelican | pellicano | 0.50 | 2.34 | 5.54 | 4.87 | 1.38 | 3.50 | 1.09 | 1.93 | 1.12 | 1.21 | 0.58 | 2.76 | 1.13 |
| Penguin | pinguino | 0.97 | 0.20 | 7.34 | 2.85 | 1.24 | 3.25 | 1.10 | 2.43 | 1.29 | 1.23 | 0.63 | 2.61 | 1.39 |
| Pheasant | fagiano | 0.35 | 2.51 | 7.13 | 5.42 | 1.58 | 3.87 | 1.18 | 2.18 | 1.10 | 1.39 | 0.71 | 2.93 | 1.35 |
| Pigeon | piccione | 0.88 | 0.73 | 7.50 | 3.20 | 1.47 | 2.93 | 1.15 | 4.16 | 1.00 | 1.21 | 0.62 | 4.11 | 0.92 |
| Raven | corvo | 0.77 | 1.43 | 7.72 | 3.85 | 1.40 | 2.24 | 0.93 | 2.56 | 1.22 | 1.19 | 0.63 | 3.81 | 1.10 |
| Rooster | gallo | 0.87 | 0.79 | 8.08 | 2.68 | 1.17 | 3.18 | 1.26 | 3.48 | 1.31 | 1.69 | 0.83 | 3.08 | 1.42 |
| Seagull | gabbiano | 0.89 | 0.70 | 7.89 | 3.51 | 1.45 | 2.99 | 1.08 | 3.16 | 1.29 | 1.16 | 0.40 | 3.60 | 1.10 |
| Sparrow | passero | 0.41 | 2.51 | 7.13 | 3.57 | 1.44 | 3.32 | 1.03 | 3.51 | 1.21 | 1.28 | 0.65 | 4.52 | 0.76 |
| Toucan | tucano | 0.46 | 2.47 | 4.73 | 5.26 | 1.48 | 3.35 | 1.01 | 1.85 | 1.15 | 1.21 | 0.54 | 3.21 | 1.33 |
| **Body pars** |  |  |  |  |  |  |  |  |  |  |  |  |  |  |
| Arm | braccio | 0.74 | 1.11 | 10.71 | 1.80 | 1.18 | 1.61 | 0.72 | 4.87 | 0.49 | 2.84 | 1.61 | 4.62 | 0.73 |
| Beard | barba | 0.20 | 1.09 | 9.52 | 3.12 | 1.78 | 3.16 | 1.26 | 3.35 | 1.54 | 2.69 | 1.28 | 2.08 | 1.08 |
| Bone | osso | 0.93 | 0.41 | 9.13 | 2.85 | 1.19 | 1.35 | 0.59 | 3.25 | 1.40 | 1.66 | 0.96 | 3.44 | 1.43 |
| Brain | cervello | 0.85 | 0.75 | 10.84 | 3.82 | 1.66 | 2.70 | 1.32 | 4.26 | 1.15 | 1.50 | 1.01 | 4.00 | 1.17 |
| Ear | orecchio | 0.99 | 0.10 | 9.77 | 1.73 | 0.86 | 2.40 | 1.09 | 4.57 | 1.14 | 1.72 | 0.95 | 4.45 | 0.84 |
| Eye | occhio | 0.99 | 0.09 | 11.23 | 1.66 | 0.85 | 3.12 | 1.38 | 4.88 | 0.42 | 1.87 | 1.14 | 4.48 | 0.92 |
| Finger | dito | 0.26 | 1.40 | 10.00 | 1.65 | 1.08 | 2.00 | 0.99 | 4.84 | 0.58 | 4.15 | 1.50 | 4.20 | 0.99 |
| Foot | piede | 0.99 | 0.10 | 10.47 | 1.59 | 0.86 | 2.33 | 1.08 | 4.71 | 0.94 | 1.79 | 0.95 | 4.64 | 0.65 |
| Hand | mano | 0.91 | 0.54 | 12.55 | 1.42 | 0.59 | 2.49 | 1.32 | 4.85 | 0.63 | 4.26 | 1.39 | 4.65 | 0.72 |
| Kidney | rene | 0.24 | 2.63 | 8.12 | 5.45 | 1.37 | 2.09 | 1.29 | 2.43 | 1.39 | 1.34 | 0.74 | 2.91 | 1.40 |
| Leg | gamba | 0.96 | 0.31 | 9.76 | 1.77 | 0.85 | 2.33 | 1.07 | 4.71 | 0.83 | 1.89 | 1.12 | 4.69 | 0.72 |
| Liver | fegato | 0.47 | 2.35 | 9.52 | 5.44 | 1.38 | 2.39 | 1.20 | 2.49 | 1.24 | 1.61 | 1.05 | 3.22 | 1.43 |
| Lung | polmone | 0.26 | 2.87 | 8.59 | 4.98 | 1.44 | 2.67 | 1.20 | 2.93 | 1.33 | 1.30 | 0.75 | 3.46 | 1.39 |
| Mouth | bocca | 0.53 | 1.00 | 11.32 | 1.61 | 0.85 | 1.91 | 0.99 | 4.77 | 0.83 | 1.85 | 1.06 | 4.40 | 0.96 |
| Nail (bodypart) | unghia | 0.89 | 0.62 | 7.58 | 2.36 | 0.92 | 2.28 | 1.08 | 4.84 | 0.53 | 3.69 | 1.41 | 3.39 | 1.46 |
| Nose | naso | 1.00 | 0.00 | 10.39 | 1.66 | 0.81 | 1.67 | 0.84 | 4.79 | 0.71 | 2.11 | 1.23 | 4.41 | 0.92 |
| Pelvis | bacino | 0.37 | 3.51 | 10.39 | 5.63 | 1.31 | 3.15 | 1.08 | 2.90 | 1.40 | 1.24 | 0.68 | 3.11 | 1.36 |
| Skull | cranio | 0.17 | 0.84 | 8.59 | 4.59 | 1.37 | 2.96 | 1.27 | 3.12 | 1.44 | 1.48 | 0.81 | 3.81 | 1.20 |
| Tongue | lingua | 0.88 | 0.70 | 12.27 | 2.04 | 1.31 | 2.44 | 1.19 | 4.70 | 0.75 | 1.38 | 0.80 | 3.55 | 1.22 |
| Vertebra | vertebra | 0.08 | 1.38 | 6.40 | 6.12 | 1.15 | 2.32 | 1.10 | 2.32 | 1.39 | 1.49 | 0.84 | 2.53 | 1.42 |
| **Flowers** |  |  |  |  |  |  |  |  |  |  |  |  |  |  |
| Araceae | calla | 0.38 | 2.59 | 5.11 | 5.36 | 1.66 | 1.96 | 0.97 | 2.55 | 1.35 | 1.84 | 0.91 | 3.58 | 1.32 |
| Bellflower | campanule | 0.11 | 3.46 | 4.60 | 5.61 | 1.62 | 2.52 | 0.76 | 2.13 | 1.18 | 1.50 | 0.69 | 3.01 | 1.32 |
| Carnation | garofano | 0.15 | 2.91 | 7.59 | 5.27 | 1.41 | 2.87 | 0.99 | 2.52 | 1.16 | 1.91 | 0.89 | 3.36 | 1.23 |
| Daisy | margherita | 0.99 | 0.09 | 8.13 | 2.42 | 0.96 | 2.04 | 1.09 | 4.31 | 1.05 | 2.07 | 1.02 | 4.79 | 0.67 |
| Geranium | geranio | 0.06 | 2.60 | 5.85 | 5.27 | 1.78 | 3.37 | 0.98 | 2.68 | 1.27 | 1.84 | 1.01 | 3.76 | 1.11 |
| Lilac | lilla | 0.05 | 3.32 | 5.68 | 5.68 | 1.48 | 2.97 | 1.25 | 2.41 | 1.25 | 1.64 | 0.80 | 3.20 | 1.26 |
| Orchid | orchidea | 0.28 | 2.53 | 6.17 | 5.42 | 1.29 | 3.33 | 1.07 | 2.85 | 1.27 | 1.81 | 1.01 | 3.85 | 1.17 |
| Pansy | viola del pensiero | 0.00 | 3.31 | 2.20 | 6.49 | 1.31 | 2.82 | 1.01 | 1.65 | 1.00 | 1.56 | 0.77 | 2.82 | 1.45 |
| Poppy | papavero | 0.80 | 1.16 | 7.21 | 3.64 | 1.42 | 2.16 | 0.96 | 3.19 | 1.28 | 1.80 | 0.85 | 3.99 | 1.10 |
| Rose | rosa | 0.96 | 0.25 | 10.60 | 2.62 | 1.06 | 2.95 | 1.25 | 4.22 | 0.96 | 2.13 | 1.09 | 4.75 | 0.69 |
| Sunflower | girasole | 1.00 | 0.00 | 7.60 | 3.15 | 1.45 | 2.52 | 0.97 | 3.83 | 1.25 | 1.79 | 1.02 | 4.45 | 0.74 |
| Tulip | tulipano | 0.72 | 1.39 | 6.38 | 3.84 | 1.39 | 1.96 | 0.76 | 3.00 | 1.14 | 1.95 | 0.85 | 4.24 | 0.90 |
| **Fruits** |  |  |  |  |  |  |  |  |  |  |  |  |  |  |
| Apple | mela | 0.96 | 0.26 | 8.94 | 1.91 | 1.06 | 1.53 | 0.84 | 4.68 | 0.71 | 3.94 | 1.11 | 4.78 | 0.70 |
| Avocado | avocado | 0.12 | 2.27 | 6.47 | 6.23 | 1.17 | 2.40 | 1.27 | 1.66 | 0.92 | 2.99 | 1.35 | 1.99 | 1.07 |
| Banana | banana | 1.00 | 0.00 | 8.15 | 1.91 | 0.96 | 1.37 | 0.67 | 4.47 | 0.91 | 3.68 | 1.10 | 4.59 | 0.76 |
| Cherries | ciliegie | 0.99 | 0.09 | 7.75 | 2.53 | 1.05 | 1.82 | 0.90 | 4.38 | 0.79 | 3.49 | 1.08 | 4.32 | 0.85 |
| Coconut | cocco | 0.36 | 2.03 | 8.33 | 3.90 | 1.29 | 2.12 | 1.12 | 3.17 | 1.34 | 3.33 | 1.29 | 2.84 | 1.18 |
| Custard apple | anona | 0.00 | 1.50 | 1.95 | 6.83 | 0.70 | 2.57 | 1.13 | 1.39 | 0.98 | 2.68 | 1.30 | 1.69 | 1.07 |
| Fig | fico | 0.75 | 1.38 | 8.37 | 3.73 | 1.49 | 1.82 | 1.04 | 3.47 | 1.25 | 3.49 | 1.11 | 3.39 | 1.24 |
| Flat peach | pesca tabacchiera | 0.04 | 2.21 | 0.00 | 4.89 | 2.02 | 2.04 | 0.85 | 2.76 | 1.46 | 3.38 | 1.30 | 2.98 | 1.37 |
| Grapes | uva | 0.79 | 1.22 | 9.33 | 2.29 | 0.97 | 2.23 | 0.95 | 3.92 | 1.17 | 3.25 | 1.16 | 4.28 | 0.88 |
| Kiwi fruit | kiwi | 0.98 | 0.18 | 7.28 | 3.49 | 1.26 | 1.86 | 0.89 | 4.06 | 1.12 | 3.72 | 1.21 | 3.84 | 1.19 |
| Lemon | limone | 1.00 | 0.00 | 9.50 | 2.20 | 1.07 | 1.34 | 0.74 | 4.39 | 0.95 | 3.35 | 1.30 | 4.02 | 1.05 |
| Mango | mango | 0.12 | 2.39 | 6.71 | 5.64 | 1.43 | 1.44 | 0.81 | 1.81 | 1.11 | 2.95 | 1.15 | 2.67 | 1.26 |
| Melon | melone | 0.09 | 2.81 | 7.42 | 3.79 | 1.43 | 2.49 | 1.03 | 3.46 | 1.12 | 3.58 | 1.19 | 3.56 | 1.15 |
| Orange | arancia | 0.94 | 0.39 | 8.60 | 2.12 | 0.88 | 1.49 | 0.76 | 4.39 | 0.98 | 3.74 | 1.27 | 4.56 | 0.79 |
| Peach | pesca | 0.32 | 2.82 | 11.34 | 2.52 | 1.25 | 1.31 | 0.68 | 3.72 | 1.30 | 3.36 | 1.04 | 4.36 | 0.90 |
| Pear | pera | 1.00 | 0.00 | 7.82 | 1.95 | 0.84 | 1.79 | 0.82 | 4.48 | 0.81 | 3.79 | 1.09 | 4.55 | 0.73 |
| Pomegranate | melagrana | 0.06 | 1.33 | 5.53 | 4.38 | 1.59 | 2.01 | 0.91 | 3.07 | 1.33 | 3.50 | 1.40 | 2.89 | 1.25 |
| Quince | mela cotogna | 0.01 | 2.46 | 4.13 | 5.47 | 1.75 | 1.56 | 0.79 | 2.32 | 1.29 | 3.08 | 1.21 | 3.05 | 1.35 |
| Redcurrant | ribes | 0.46 | 2.90 | 6.89 | 5.39 | 1.55 | 2.69 | 1.06 | 2.67 | 1.32 | 3.31 | 1.10 | 2.98 | 1.31 |
| Strawberry | fragola | 1.00 | 0.00 | 7.57 | 2.23 | 1.02 | 2.17 | 1.06 | 4.43 | 0.89 | 3.17 | 1.26 | 4.63 | 0.62 |
| Watermelon | anguria | 0.87 | 0.57 | 6.86 | 3.00 | 1.22 | 1.69 | 0.79 | 4.01 | 1.07 | 3.41 | 1.07 | 3.97 | 1.03 |
| **Insects** |  |  |  |  |  |  |  |  |  |  |  |  |  |  |
| Ant | formica | 0.92 | 0.59 | 7.52 | 2.41 | 1.28 | 1.82 | 0.97 | 3.82 | 1.36 | 1.42 | 0.81 | 4.39 | 1.00 |
| Bee | ape | 0.54 | 2.23 | 8.23 | 2.33 | 1.08 | 3.22 | 1.01 | 3.79 | 1.07 | 1.35 | 0.76 | 4.29 | 1.00 |
| Beetle | scarabeo | 0.44 | 2.11 | 6.29 | 4.87 | 1.46 | 2.67 | 1.00 | 2.17 | 1.08 | 1.33 | 0.76 | 3.79 | 1.09 |
| Butterfly | farfalla | 0.99 | 0.09 | 8.67 | 2.31 | 1.10 | 2.51 | 1.19 | 3.92 | 1.11 | 1.41 | 0.71 | 4.14 | 1.14 |
| Centipede | millepiedi | 0.45 | 2.29 | 5.81 | 4.21 | 1.50 | 3.15 | 1.11 | 2.27 | 1.18 | 1.22 | 0.57 | 3.13 | 1.39 |
| Cockroach | scarafaggio | 0.49 | 2.19 | 6.19 | 3.96 | 1.43 | 2.91 | 1.03 | 2.29 | 1.10 | 1.19 | 0.51 | 4.04 | 1.11 |
| Dragonfly | libellula | 0.65 | 1.77 | 5.97 | 4.28 | 1.53 | 3.25 | 1.15 | 2.55 | 1.22 | 1.24 | 0.45 | 3.41 | 1.27 |
| Fly | mosca | 0.93 | 0.54 | 8.43 | 2.65 | 1.22 | 3.05 | 1.13 | 4.13 | 1.05 | 1.55 | 0.97 | 4.34 | 1.08 |
| Grasshopper | cavalletta | 0.72 | 1.47 | 5.64 | 3.81 | 1.30 | 3.79 | 1.04 | 2.63 | 1.08 | 1.32 | 0.74 | 3.76 | 1.02 |
| Ladybird | coccinella | 1.00 | 0.00 | 5.79 | 2.76 | 1.20 | 2.55 | 1.12 | 3.82 | 1.10 | 1.36 | 0.63 | 4.09 | 1.01 |
| Mosquito | zanzara | 0.73 | 1.46 | 7.87 | 3.05 | 1.20 | 2.52 | 1.02 | 4.15 | 1.06 | 1.82 | 1.08 | 4.28 | 1.01 |
| Moth | tarma | 0.01 | 2.46 | 4.62 | 6.04 | 1.13 | 3.31 | 1.15 | 1.91 | 1.03 | 1.19 | 0.48 | 2.73 | 1.19 |
| Praying mantis | mantide religiosa | 0.41 | 2.64 | 4.01 | 5.65 | 1.36 | 3.76 | 1.16 | 2.15 | 1.24 | 1.24 | 0.53 | 3.22 | 1.39 |
| Scorpion | scorpione | 0.90 | 0.64 | 7.48 | 4.10 | 1.27 | 3.44 | 1.08 | 2.32 | 1.31 | 1.15 | 0.55 | 2.90 | 1.18 |
| Spider | ragno | 0.68 | 1.11 | 8.49 | 2.68 | 1.16 | 2.33 | 0.99 | 3.07 | 1.32 | 1.24 | 0.54 | 4.23 | 1.10 |
| Termite | termite | 0.01 | 2.62 | 4.36 | 6.11 | 1.15 | 2.84 | 1.15 | 1.60 | 0.88 | 1.28 | 0.61 | 2.84 | 1.45 |
| Wasp | vespa | 0.46 | 1.16 | 7.37 | 3.18 | 1.28 | 3.43 | 1.04 | 3.50 | 1.11 | 1.37 | 0.84 | 4.16 | 0.88 |
| **Marine Creatures** |  |  |  |  |  |  |  |  |  |  |  |  |  |  |
| Cockle | conchiglia | 0.89 | 0.60 | 7.91 | 2.43 | 1.33 | 2.37 | 0.94 | 3.27 | 1.33 | 1.96 | 0.95 | 2.73 | 1.34 |
| Crab | granchio | 0.94 | 0.44 | 7.06 | 3.40 | 1.22 | 3.11 | 1.19 | 2.89 | 1.28 | 1.69 | 1.05 | 3.89 | 1.11 |
| Dolphin | delfino | 0.95 | 0.35 | 8.06 | 3.04 | 1.21 | 2.30 | 0.95 | 2.93 | 1.35 | 1.32 | 0.68 | 4.43 | 0.89 |
| Eel | anguilla | 0.41 | 2.54 | 7.39 | 4.69 | 1.44 | 1.49 | 0.89 | 1.89 | 1.09 | 1.32 | 0.72 | 2.83 | 1.26 |
| Goose barnacle | cirripedi | 0.00 | 0.99 | 3.14 | 6.78 | 0.86 | 3.99 | 1.04 | 1.02 | 0.15 | 1.39 | 0.79 | 1.44 | 0.88 |
| Killer whale | orca | 0.71 | 1.35 | 5.77 | 3.89 | 1.49 | 2.45 | 0.90 | 2.44 | 1.25 | 1.16 | 0.48 | 3.79 | 1.14 |
| Lobster | aragosta | 0.59 | 2.09 | 7.19 | 5.00 | 1.26 | 3.41 | 1.13 | 2.44 | 1.18 | 2.08 | 1.25 | 3.56 | 1.11 |
| Manatee | lamantino | 0.05 | 3.09 | 2.94 | 6.33 | 1.18 | 2.73 | 1.06 | 1.54 | 1.02 | 1.19 | 0.57 | 2.80 | 1.47 |
| Mussel | cozza | 0.85 | 0.88 | 7.20 | 4.07 | 1.33 | 2.02 | 1.08 | 3.20 | 1.28 | 3.12 | 1.34 | 2.55 | 1.26 |
| Narwhal | narvalo | 0.07 | 2.62 | 4.51 | 6.43 | 1.12 | 2.93 | 0.95 | 1.23 | 0.76 | 1.13 | 0.45 | 2.40 | 1.42 |
| Oyster | ostrica | 0.16 | 2.52 | 6.09 | 5.61 | 1.37 | 3.16 | 1.21 | 2.19 | 1.18 | 2.71 | 1.25 | 2.51 | 1.15 |
| Pomfret | pampo | 0.00 | 2.50 | 0.69 | 6.55 | 1.28 | 2.93 | 1.06 | 1.11 | 0.47 | 1.74 | 1.13 | 2.26 | 1.49 |
| Ray | razza | 0.33 | 2.68 | 10.54 | 5.69 | 1.37 | 3.23 | 0.86 | 1.72 | 0.99 | 1.25 | 0.52 | 2.80 | 1.39 |
| Razor-shell | cannolicchio | 0.02 | 1.51 | 4.67 | 6.55 | 1.13 | 2.24 | 1.31 | 1.42 | 0.98 | 2.36 | 1.25 | 1.69 | 0.94 |
| Shark | squalo | 0.54 | 2.11 | 8.20 | 3.38 | 1.44 | 2.73 | 0.92 | 2.44 | 1.34 | 1.10 | 0.30 | 4.43 | 0.86 |
| Sperm whale | capodoglio | 0.08 | 1.92 | 5.27 | 5.81 | 1.47 | 2.40 | 0.94 | 1.44 | 0.81 | 1.16 | 0.52 | 3.24 | 1.50 |
| Starfish | stella marina | 1.00 | 0.00 | 5.08 | 3.52 | 1.35 | 2.85 | 1.15 | 2.81 | 1.42 | 1.45 | 0.84 | 3.94 | 1.19 |
| Whale | balena | 0.33 | 2.05 | 8.16 | 3.52 | 1.42 | 2.74 | 0.98 | 2.34 | 1.33 | 1.17 | 0.54 | 4.09 | 1.07 |
| **Nuts** |  |  |  |  |  |  |  |  |  |  |  |  |  |  |
| Acorn | ghianda | 0.65 | 1.78 | 6.18 | 4.13 | 1.38 | 2.23 | 0.86 | 2.40 | 1.14 | 2.27 | 1.12 | 2.35 | 1.18 |
| Almond | mandorla | 0.37 | 2.33 | 7.74 | 4.27 | 1.55 | 2.19 | 1.07 | 3.39 | 1.30 | 3.39 | 1.13 | 3.94 | 1.15 |
| Chestnut | castagna | 0.72 | 1.34 | 7.03 | 3.28 | 1.31 | 1.71 | 0.90 | 3.46 | 1.32 | 3.38 | 1.37 | 3.44 | 1.19 |
| Date | dattero | 0.35 | 2.47 | 5.78 | 5.23 | 1.34 | 2.07 | 0.99 | 2.21 | 1.20 | 2.61 | 1.13 | 3.40 | 1.27 |
| Hazelnut | nocciola | 0.42 | 2.66 | 7.40 | 3.61 | 1.34 | 1.76 | 0.91 | 3.54 | 1.22 | 3.38 | 1.12 | 4.01 | 0.98 |
| Peanut | arachide | 0.59 | 1.79 | 5.33 | 4.33 | 1.35 | 2.05 | 0.94 | 3.65 | 1.16 | 3.72 | 1.08 | 4.23 | 1.05 |
| Pine kernel | pinolo | 0.01 | 2.09 | 4.44 | 4.68 | 1.63 | 1.51 | 0.92 | 2.28 | 1.09 | 2.57 | 1.26 | 2.76 | 1.10 |
| Pipe | seme | 0.02 | 1.84 | 9.40 | 4.05 | 1.75 | 2.55 | 1.28 | 2.61 | 1.20 | 2.42 | 1.12 | 1.96 | 1.09 |
| Pistachio | pistacchio | 0.72 | 1.57 | 7.25 | 3.94 | 1.34 | 2.11 | 0.92 | 3.52 | 1.20 | 3.76 | 1.22 | 3.98 | 1.02 |
| Raisin | uva passa | 0.08 | 3.17 | 6.39 | 5.13 | 1.40 | 2.49 | 1.17 | 2.40 | 1.21 | 2.76 | 1.17 | 3.56 | 1.27 |
| Walnut | noce | 1.00 | 0.00 | 8.84 | 3.20 | 1.28 | 2.00 | 0.94 | 4.15 | 1.01 | 3.69 | 1.10 | 4.47 | 0.78 |
| **Trees** |  |  |  |  |  |  |  |  |  |  |  |  |  |  |
| Black poplar | pioppo | 0.06 | 2.82 | 7.01 | 5.39 | 1.65 | 2.59 | 1.14 | 2.40 | 1.18 | 1.22 | 0.50 | 3.61 | 1.16 |
| Cedar | cedro | 0.00 | 1.40 | 7.29 | 5.57 | 1.73 | 2.61 | 1.13 | 1.96 | 1.12 | 1.47 | 0.79 | 3.45 | 1.31 |
| Cypress | cipresso | 0.27 | 2.29 | 6.74 | 5.12 | 1.39 | 2.13 | 1.06 | 3.12 | 1.07 | 1.64 | 0.92 | 3.85 | 1.11 |
| Eucalyptus | eucalipto | 0.00 | 2.14 | 6.38 | 6.26 | 1.30 | 2.98 | 1.14 | 1.63 | 0.81 | 1.22 | 0.47 | 2.80 | 1.39 |
| Fig tree | fico | 0.04 | 2.36 | 8.37 | 4.68 | 1.68 | 3.04 | 1.21 | 2.48 | 1.34 | 1.79 | 1.02 | 3.29 | 1.21 |
| Fir | abete | 0.42 | 1.93 | 7.88 | 3.99 | 1.36 | 2.82 | 1.16 | 3.68 | 1.22 | 1.69 | 0.99 | 4.55 | 0.75 |
| Holm oak | leccio | 0.00 | 2.53 | 6.58 | 6.23 | 1.60 | 3.11 | 1.22 | 1.78 | 1.21 | 1.27 | 0.50 | 3.06 | 1.60 |
| Olive tree | olivo | 0.04 | 2.63 | 7.88 | 4.31 | 1.51 | 3.11 | 1.11 | 2.97 | 1.29 | 1.73 | 0.89 | 3.77 | 1.11 |
| Palm tree | palma | 0.96 | 0.25 | 8.46 | 4.00 | 1.28 | 3.20 | 1.07 | 3.34 | 1.19 | 1.46 | 0.73 | 3.47 | 1.26 |
| Pine tree | pino | 0.02 | 2.56 | 8.55 | 4.29 | 1.72 | 2.44 | 1.02 | 2.95 | 1.37 | 1.17 | 0.41 | 4.12 | 0.99 |
| Willow | salice | 0.35 | 2.16 | 7.03 | 4.40 | 1.36 | 2.93 | 1.14 | 2.84 | 1.29 | 1.33 | 0.58 | 3.84 | 1.09 |
| **Vegetables** |  |  |  |  |  |  |  |  |  |  |  |  |  |  |
| Artichoke | carciofo | 0.93 | 0.53 | 7.14 | 4.27 | 1.41 | 2.64 | 1.03 | 3.67 | 1.30 | 3.59 | 1.14 | 3.71 | 1.18 |
| Asparagus | asparago | 0.78 | 1.42 | 6.17 | 4.74 | 1.65 | 2.00 | 0.89 | 3.10 | 1.34 | 3.16 | 1.27 | 3.05 | 1.09 |
| Cabbage | cavolo | 0.09 | 2.47 | 9.32 | 4.23 | 1.37 | 2.23 | 0.98 | 3.37 | 1.16 | 3.16 | 1.14 | 3.87 | 0.99 |
| Carrot | carota | 0.99 | 0.09 | 8.22 | 2.47 | 1.00 | 1.58 | 0.71 | 4.47 | 0.84 | 3.62 | 1.03 | 4.36 | 0.92 |
| Cauliflower | cavolfiore | 0.76 | 1.08 | 6.62 | 4.29 | 1.49 | 2.84 | 1.08 | 3.50 | 1.32 | 3.28 | 1.18 | 3.60 | 1.12 |
| Celery | sedano | 0.87 | 0.92 | 8.34 | 4.48 | 1.21 | 2.01 | 0.85 | 3.43 | 1.16 | 3.35 | 1.10 | 3.67 | 1.09 |
| Chard | bietola | 0.08 | 3.25 | 5.55 | 5.64 | 1.58 | 2.46 | 0.95 | 2.79 | 1.39 | 3.41 | 1.09 | 3.38 | 1.30 |
| Cucumber | cetriolo | 0.87 | 0.72 | 6.76 | 4.01 | 1.37 | 1.68 | 0.94 | 3.49 | 1.41 | 3.27 | 1.32 | 3.66 | 1.15 |
| Eggplant | melanzana | 0.97 | 0.20 | 6.82 | 3.19 | 1.27 | 1.48 | 0.66 | 4.15 | 0.97 | 3.31 | 1.21 | 4.01 | 1.06 |
| Endive | indivia | 0.00 | 2.35 | 5.81 | 6.14 | 1.49 | 3.08 | 1.24 | 2.58 | 1.46 | 3.38 | 1.13 | 3.27 | 1.28 |
| Leek | porro | 0.42 | 2.87 | 6.68 | 5.38 | 1.65 | 2.38 | 0.94 | 2.72 | 1.41 | 3.10 | 1.33 | 2.93 | 1.19 |
| Lettuce | lattuga | 0.32 | 2.17 | 7.45 | 4.03 | 1.34 | 2.28 | 1.03 | 3.71 | 1.25 | 3.16 | 1.15 | 4.27 | 0.81 |
| Onion | cipolla | 0.91 | 0.66 | 9.32 | 3.19 | 1.12 | 1.62 | 0.83 | 4.31 | 1.01 | 3.72 | 1.11 | 3.49 | 1.12 |
| Pepper | peperone | 0.82 | 0.75 | 7.42 | 3.43 | 1.46 | 1.74 | 0.80 | 3.93 | 1.20 | 3.22 | 1.34 | 4.09 | 0.92 |
| Potato | patata | 0.99 | 0.10 | 8.24 | 2.32 | 0.95 | 1.55 | 0.84 | 4.44 | 0.99 | 3.53 | 1.21 | 3.87 | 1.08 |
| Pumpkin | zucca | 0.75 | 1.35 | 8.62 | 3.94 | 1.49 | 1.73 | 0.97 | 3.39 | 1.34 | 3.07 | 1.28 | 3.31 | 1.27 |
| Red cabbage | cavolo rosso | 0.01 | 2.73 | 4.23 | 5.80 | 1.54 | 2.22 | 1.28 | 1.73 | 1.02 | 2.98 | 1.42 | 2.29 | 1.39 |
| Spinach | spinaci | 0.37 | 3.07 | 8.07 | 3.67 | 1.52 | 2.25 | 0.99 | 3.41 | 1.16 | 3.12 | 1.09 | 3.93 | 0.99 |
| Tomato | pomodoro | 1.00 | 0.00 | 9.36 | 2.39 | 0.95 | 1.45 | 0.75 | 4.65 | 0.88 | 3.85 | 1.02 | 4.48 | 0.95 |
| Turnip | rapa | 0.00 | 0.89 | 6.83 | 5.49 | 1.44 | 2.29 | 1.25 | 1.57 | 1.07 | 2.70 | 1.50 | 2.18 | 1.25 |
| **Nature** |  |  |  |  |  |  |  |  |  |  |  |  |  |  |
| Cliff | scogliera | 0.45 | 3.00 | 7.98 | 4.35 | 1.46 | 3.72 | 1.25 | 2.96 | 1.21 | 1.37 | 0.75 | 3.26 | 1.10 |
| Cloud | nuvola | 0.92 | 0.45 | 8.74 | 1.88 | 1.01 | 2.05 | 1.24 | 4.61 | 0.90 | 1.07 | 0.41 | 3.89 | 1.23 |
| Coal | carbone | 0.09 | 3.04 | 9.47 | 4.62 | 1.86 | 2.22 | 1.29 | 2.09 | 1.06 | 1.98 | 0.95 | 2.00 | 1.14 |
| Gold | oro | 0.16 | 1.84 | 11.37 | 3.68 | 1.71 | 3.71 | 1.18 | 2.66 | 1.27 | 2.40 | 1.23 | 1.62 | 0.88 |
| Ice | ghiaccio | 0.20 | 1.95 | 10.05 | 3.16 | 1.38 | 2.76 | 1.16 | 3.04 | 1.27 | 1.53 | 0.84 | 3.32 | 1.09 |
| Iceberg | iceberg | 0.86 | 0.79 | 8.08 | 4.93 | 1.30 | 2.86 | 1.21 | 2.27 | 1.33 | 1.05 | 0.21 | 3.06 | 1.31 |
| Island | isola | 0.91 | 0.60 | 11.39 | 3.12 | 1.21 | 2.66 | 1.18 | 3.46 | 1.26 | 1.16 | 0.46 | 3.22 | 1.28 |
| Moon | luna | 0.84 | 0.80 | 10.51 | 1.76 | 0.91 | 1.67 | 0.89 | 4.53 | 1.03 | 1.03 | 0.16 | 3.76 | 1.21 |
| Mountain | montagna | 0.83 | 1.18 | 10.98 | 2.51 | 1.31 | 2.84 | 1.23 | 4.04 | 1.15 | 1.64 | 1.04 | 4.49 | 0.83 |
| Puddle | pozzanghera | 0.35 | 2.06 | 6.52 | 3.05 | 1.47 | 3.10 | 1.31 | 3.99 | 1.00 | 1.15 | 0.42 | 1.85 | 1.01 |
| Sea | mare | 0.95 | 0.30 | 12.28 | 1.64 | 0.80 | 1.56 | 0.98 | 4.37 | 0.87 | 1.96 | 1.18 | 4.44 | 0.79 |
| Stone | pietra | 0.18 | 2.77 | 10.82 | 3.08 | 1.52 | 2.34 | 1.22 | 3.47 | 1.34 | 2.06 | 1.09 | 2.85 | 1.26 |
| Sun | sole | 0.96 | 0.26 | 11.92 | 1.51 | 0.91 | 1.96 | 1.08 | 4.91 | 0.32 | 1.11 | 0.44 | 4.15 | 1.11 |
| Volcano | vulcano | 0.97 | 0.18 | 8.64 | 3.51 | 1.35 | 2.37 | 1.04 | 2.79 | 1.44 | 1.05 | 0.23 | 3.75 | 1.15 |
| Waterfall | cascata | 0.80 | 0.79 | 8.77 | 3.84 | 1.17 | 3.89 | 1.28 | 2.65 | 1.22 | 1.15 | 0.57 | 4.12 | 0.91 |
| Wave | onda | 0.44 | 2.43 | 10.90 | 2.63 | 1.31 | 2.99 | 1.13 | 3.71 | 1.16 | 1.35 | 0.81 | 3.61 | 1.19 |
| **Buildings** |  |  |  |  |  |  |  |  |  |  |  |  |  |  |
| Castle | castello | 0.72 | 1.52 | 10.49 | 2.91 | 1.30 | 3.04 | 1.07 | 2.97 | 1.35 | 1.87 | 1.12 | 3.31 | 1.13 |
| Cathedral | cattedrale | 0.45 | 2.26 | 9.15 | 5.28 | 1.31 | 4.39 | 0.90 | 3.21 | 1.01 | 1.82 | 1.14 | 3.24 | 1.29 |
| Church | chiesa | 0.68 | 1.52 | 11.54 | 2.79 | 1.58 | 3.20 | 1.06 | 3.90 | 1.16 | 1.82 | 1.15 | 3.76 | 1.06 |
| Factory | fabbrica | 0.49 | 2.51 | 10.38 | 4.39 | 1.51 | 4.36 | 1.00 | 3.16 | 1.33 | 2.97 | 1.48 | 3.45 | 1.19 |
| Granary | granaio | 0.00 | 2.07 | 6.97 | 5.71 | 1.51 | 3.80 | 1.02 | 1.69 | 1.00 | 2.42 | 1.18 | 1.76 | 0.92 |
| House | casa | 0.83 | 1.21 | 13.23 | 1.61 | 1.11 | 2.85 | 1.20 | 4.90 | 0.43 | 2.41 | 1.34 | 4.78 | 0.67 |
| Lighthouse | faro | 0.77 | 1.41 | 8.98 | 4.40 | 1.52 | 2.93 | 1.12 | 2.33 | 1.09 | 2.00 | 1.20 | 2.52 | 1.26 |
| Mill | mulino | 0.58 | 1.49 | 8.43 | 4.29 | 1.32 | 3.00 | 1.08 | 2.51 | 1.39 | 1.98 | 1.21 | 2.00 | 1.05 |
| Pagoda | pagoda | 0.15 | 3.47 | 6.51 | 6.20 | 1.33 | 3.91 | 1.10 | 1.59 | 0.97 | 1.68 | 1.08 | 2.15 | 1.18 |
| Palace | palazzo | 0.18 | 3.38 | 10.89 | 3.64 | 1.58 | 4.43 | 0.86 | 3.08 | 1.37 | 1.96 | 1.13 | 3.83 | 1.20 |
| Pyramid | piramide | 0.91 | 0.57 | 8.70 | 4.09 | 1.31 | 2.45 | 1.18 | 2.64 | 1.49 | 1.59 | 0.97 | 1.81 | 1.15 |
| Shanty | baracca | 0.51 | 2.32 | 8.31 | 5.93 | 1.27 | 3.71 | 1.13 | 1.85 | 0.97 | 1.98 | 1.20 | 1.71 | 0.85 |
| Silo | silo | 0.01 | 2.26 | 5.01 | 6.24 | 1.10 | 2.25 | 0.99 | 1.85 | 1.12 | 1.91 | 1.14 | 1.93 | 1.00 |
| Skyscraper | grattacielo | 0.88 | 0.58 | 7.92 | 4.21 | 1.27 | 3.49 | 1.13 | 3.18 | 1.25 | 1.84 | 1.25 | 4.49 | 0.78 |
| Tower | torre | 0.88 | 0.89 | 9.92 | 3.30 | 1.16 | 3.40 | 1.22 | 2.91 | 1.29 | 1.60 | 0.98 | 2.93 | 1.23 |
| **Clothing** |  |  |  |  |  |  |  |  |  |  |  |  |  |  |
| Bathrobe | accappatoio | 1.00 | 0.00 | 6.54 | 3.28 | 1.21 | 1.99 | 0.92 | 4.63 | 0.87 | 3.67 | 0.95 | 2.73 | 1.13 |
| Biretta | tocco | 0.13 | 3.03 | 9.54 | 6.73 | 0.68 | 2.26 | 1.01 | 2.01 | 1.26 | 2.71 | 1.18 | 1.81 | 1.18 |
| Cap | cappello | 0.46 | 2.25 | 9.48 | 2.15 | 1.25 | 1.77 | 0.71 | 3.88 | 1.16 | 2.90 | 1.16 | 3.33 | 1.18 |
| Clog | zoccolo | 0.39 | 1.82 | 7.82 | 5.16 | 1.49 | 3.07 | 1.07 | 1.85 | 1.19 | 2.04 | 0.96 | 1.69 | 0.96 |
| Coat | cappotto | 0.92 | 0.57 | 8.25 | 3.55 | 1.40 | 2.16 | 0.91 | 4.33 | 0.94 | 3.51 | 1.04 | 4.28 | 0.81 |
| Glove | guanto | 0.78 | 1.06 | 7.87 | 2.82 | 1.22 | 1.88 | 0.81 | 4.12 | 1.00 | 4.68 | 0.86 | 3.09 | 1.14 |
| Jacket | giacca | 0.91 | 0.65 | 9.27 | 3.24 | 1.31 | 2.03 | 0.88 | 3.87 | 1.19 | 3.43 | 1.00 | 4.48 | 0.62 |
| Shirt | camicia | 1.00 | 0.00 | 9.28 | 3.25 | 1.11 | 2.36 | 0.90 | 4.41 | 0.81 | 3.61 | 1.19 | 4.52 | 0.68 |
| Shoe | scarpa | 0.83 | 1.07 | 8.33 | 1.84 | 1.05 | 2.09 | 0.89 | 4.74 | 0.80 | 3.32 | 1.29 | 4.26 | 0.83 |
| Skirt | gonna | 0.96 | 0.31 | 8.54 | 2.43 | 1.08 | 1.28 | 0.53 | 3.95 | 1.24 | 3.27 | 1.11 | 4.33 | 0.81 |
| Socks | calzini | 0.58 | 1.87 | 7.48 | 2.31 | 0.98 | 1.48 | 0.78 | 4.79 | 0.73 | 3.80 | 1.22 | 3.94 | 1.11 |
| Trousers | pantaloni | 0.95 | 0.38 | 9.41 | 1.88 | 1.06 | 1.60 | 0.73 | 4.84 | 0.55 | 3.46 | 1.22 | 4.82 | 0.42 |
| Undershirt | canottiera | 0.79 | 0.86 | 6.89 | 2.55 | 1.03 | 1.23 | 0.42 | 4.31 | 1.04 | 3.21 | 1.12 | 3.68 | 1.02 |
| **Desk Material** |  |  |  |  |  |  |  |  |  |  |  |  |  |  |
| Compasses | compasso | 0.98 | 0.18 | 7.01 | 4.89 | 1.24 | 2.49 | 1.02 | 3.26 | 1.29 | 4.84 | 0.51 | 3.36 | 1.15 |
| Eraser | gomma | 0.51 | 2.08 | 9.72 | 2.52 | 0.98 | 1.74 | 1.03 | 4.46 | 0.89 | 4.55 | 0.83 | 4.59 | 0.67 |
| Felt-tip pen | pennarello | 0.65 | 1.55 | 7.07 | 2.59 | 1.23 | 1.89 | 0.85 | 4.37 | 1.06 | 4.84 | 0.47 | 4.35 | 0.85 |
| Folder | cartella | 0.06 | 3.43 | 10.05 | 4.53 | 1.34 | 1.69 | 0.79 | 4.21 | 0.91 | 4.05 | 0.91 | 3.55 | 1.13 |
| Fountain pen | penna stilografica | 0.54 | 2.13 | 5.19 | 4.39 | 1.39 | 2.33 | 0.93 | 3.13 | 1.34 | 4.90 | 0.34 | 3.44 | 1.11 |
| Ink pad | tampone di inchiostro | 0.00 | 2.75 | 0.00 | 5.25 | 1.42 | 2.09 | 0.82 | 2.39 | 1.21 | 3.99 | 1.02 | 2.55 | 1.26 |
| Paperclip | graffetta | 0.74 | 1.40 | 4.92 | 4.55 | 1.32 | 1.45 | 0.72 | 4.28 | 0.93 | 4.24 | 0.97 | 3.62 | 1.10 |
| Pen | penna | 0.65 | 1.74 | 9.65 | 2.29 | 1.17 | 1.65 | 0.69 | 4.78 | 0.67 | 4.88 | 0.53 | 4.84 | 0.46 |
| Pencil | matita | 0.97 | 0.20 | 8.82 | 2.19 | 0.94 | 1.17 | 0.48 | 4.87 | 0.68 | 4.87 | 0.53 | 4.85 | 0.43 |
| Pencil sharpener | temperino | 0.78 | 0.96 | 5.63 | 3.52 | 1.22 | 2.13 | 0.86 | 4.52 | 0.80 | 4.65 | 0.67 | 4.34 | 0.84 |
| Rubber stamp | timbro | 0.80 | 1.29 | 9.54 | 4.30 | 1.33 | 2.49 | 0.86 | 2.43 | 1.18 | 4.56 | 0.82 | 2.04 | 1.15 |
| Ruler | righello | 0.94 | 0.33 | 6.33 | 3.17 | 1.13 | 1.73 | 0.80 | 4.32 | 0.90 | 4.39 | 0.80 | 4.28 | 0.88 |
| Set square | squadra | 0.89 | 0.49 | 11.88 | 4.80 | 1.24 | 1.68 | 0.76 | 3.16 | 1.27 | 4.43 | 0.79 | 3.43 | 1.12 |
| Square ruler | squadra | 0.91 | 0.60 | 11.88 | 4.61 | 1.25 | 1.89 | 1.06 | 3.65 | 1.08 | 4.52 | 0.93 | 3.60 | 1.13 |
| Stapler | pinzatrice | 0.00 | 1.65 | 3.47 | 5.65 | 1.22 | 3.68 | 1.16 | 2.28 | 1.24 | 4.44 | 0.89 | 2.53 | 1.19 |
| **Food** |  |  |  |  |  |  |  |  |  |  |  |  |  |  |
| Anchovy | acciughe | 0.36 | 2.47 | 8.09 | 5.15 | 1.44 | 2.39 | 1.18 | 2.75 | 1.26 | 2.67 | 1.15 | 2.60 | 1.31 |
| Black pudding | sanguinaccio | 0.00 | 1.34 | 4.83 | 6.40 | 1.15 | 1.98 | 1.20 | 1.32 | 0.78 | 2.65 | 1.30 | 1.84 | 1.07 |
| Caviar | caviale | 0.41 | 2.48 | 6.99 | 5.89 | 1.21 | 2.65 | 1.22 | 1.68 | 1.07 | 2.55 | 1.15 | 2.17 | 1.22 |
| Cheese | formaggio | 0.63 | 2.05 | 9.71 | 2.92 | 1.47 | 2.31 | 1.14 | 4.32 | 0.98 | 3.64 | 0.97 | 3.89 | 1.01 |
| Chorizo | salsiccia | 0.39 | 2.87 | 7.82 | 3.56 | 1.34 | 2.11 | 1.12 | 3.34 | 1.14 | 3.00 | 1.18 | 3.15 | 1.23 |
| Cookie | biscotto | 0.84 | 0.96 | 7.25 | 1.93 | 1.24 | 1.84 | 0.85 | 4.45 | 1.02 | 4.05 | 0.96 | 4.04 | 0.85 |
| Cr√®me caramel | creme caramel | 0.45 | 2.26 | 3.85 | 5.18 | 1.31 | 2.60 | 1.10 | 3.40 | 1.22 | 3.75 | 1.08 | 3.08 | 1.31 |
| Fritter | frittella | 0.00 | 2.58 | 5.34 | 4.24 | 1.82 | 2.16 | 0.96 | 2.72 | 1.28 | 3.43 | 1.24 | 2.61 | 1.17 |
| Millefeuille | millefoglie | 0.05 | 3.27 | 5.43 | 4.96 | 1.58 | 2.62 | 1.17 | 3.28 | 1.32 | 3.88 | 1.06 | 2.84 | 1.25 |
| Paella | paella | 0.83 | 1.23 | 6.10 | 6.09 | 1.14 | 4.12 | 1.01 | 2.97 | 1.21 | 3.68 | 1.04 | 3.07 | 1.15 |
| Pasty | panzerotto | 0.27 | 2.84 | 3.26 | 5.51 | 1.51 | 2.44 | 1.01 | 2.91 | 1.39 | 3.67 | 1.07 | 2.87 | 1.21 |
| Pie | torta | 0.16 | 2.80 | 9.37 | 2.51 | 1.29 | 2.34 | 1.09 | 3.99 | 0.99 | 3.93 | 1.19 | 3.72 | 1.08 |
| Steak | bistecca | 0.37 | 2.61 | 7.54 | 3.16 | 1.49 | 3.11 | 1.40 | 4.19 | 1.12 | 3.57 | 1.10 | 4.16 | 0.94 |
| **Furniture** |  |  |  |  |  |  |  |  |  |  |  |  |  |  |
| Armchair | poltrona | 0.99 | 0.09 | 9.40 | 3.16 | 1.13 | 2.22 | 0.89 | 4.51 | 0.83 | 2.25 | 1.03 | 3.87 | 1.03 |
| Bed | letto | 0.90 | 0.63 | 12.13 | 1.65 | 0.99 | 2.35 | 0.93 | 4.90 | 0.34 | 2.57 | 1.21 | 4.12 | 0.99 |
| Bedside table | comodino | 0.74 | 1.51 | 7.96 | 3.25 | 1.16 | 2.63 | 0.96 | 4.27 | 1.22 | 3.13 | 1.20 | 4.04 | 0.99 |
| Bookcase | libreria | 0.94 | 0.44 | 10.03 | 3.73 | 1.11 | 3.48 | 1.17 | 4.55 | 0.66 | 3.80 | 1.09 | 4.24 | 0.87 |
| Chair | sedia | 0.96 | 0.28 | 9.79 | 1.77 | 1.03 | 2.01 | 0.88 | 4.79 | 0.72 | 2.32 | 1.11 | 4.44 | 0.89 |
| Chest of drawers | cassettiera | 0.41 | 2.83 | 5.55 | 4.20 | 1.40 | 2.48 | 1.03 | 4.05 | 1.01 | 3.69 | 1.09 | 4.24 | 0.91 |
| Couch | divano | 0.36 | 3.07 | 9.11 | 3.42 | 1.73 | 3.08 | 1.00 | 4.20 | 1.18 | 2.01 | 1.02 | 3.89 | 1.15 |
| Filling cabinet | schedario | 0.07 | 3.46 | 7.38 | 5.85 | 1.23 | 2.49 | 0.95 | 2.63 | 1.22 | 3.79 | 0.94 | 2.11 | 1.08 |
| Lamp | lampada | 0.81 | 0.94 | 8.88 | 2.87 | 1.15 | 2.60 | 0.96 | 4.25 | 1.03 | 3.48 | 1.04 | 3.44 | 1.21 |
| Lectern | leggio | 0.13 | 3.12 | 6.25 | 5.68 | 1.30 | 2.48 | 0.97 | 2.11 | 1.11 | 2.88 | 1.12 | 1.84 | 1.01 |
| Rocking chair | sedia a dondolo | 0.96 | 0.28 | 5.35 | 3.26 | 1.32 | 3.22 | 1.27 | 3.11 | 1.27 | 2.18 | 0.94 | 2.72 | 1.21 |
| Sofa | sofà | 0.00 | 0.10 | 6.24 | 3.53 | 1.55 | 2.47 | 1.09 | 4.64 | 0.88 | 2.19 | 1.05 | 4.16 | 0.87 |
| Stool | sgabello | 0.95 | 0.34 | 7.38 | 3.73 | 1.27 | 2.04 | 0.85 | 4.19 | 0.88 | 2.52 | 1.13 | 3.31 | 1.17 |
| Table | tavolo | 0.89 | 0.71 | 11.07 | 1.87 | 0.99 | 1.77 | 0.86 | 4.73 | 0.74 | 2.30 | 1.17 | 4.66 | 0.71 |
| Wardrobe | armadio | 0.95 | 0.41 | 8.88 | 2.81 | 1.01 | 1.89 | 0.76 | 4.60 | 0.97 | 3.48 | 1.13 | 4.72 | 0.48 |
| **Jewellery** |  |  |  |  |  |  |  |  |  |  |  |  |  |  |
| Bangle | braccialetto | 0.40 | 2.23 | 7.03 | 3.19 | 1.30 | 2.71 | 1.04 | 4.33 | 0.96 | 3.92 | 1.11 | 4.09 | 1.04 |
| Bracelet | braccialetto | 0.50 | 1.43 | 7.03 | 2.88 | 1.22 | 2.91 | 1.10 | 4.43 | 0.86 | 3.88 | 1.07 | 4.59 | 0.74 |
| Brooch | spilla | 0.72 | 1.59 | 6.77 | 4.00 | 1.39 | 4.05 | 0.98 | 2.87 | 1.26 | 3.89 | 0.91 | 2.97 | 1.11 |
| Cufflinks | gemelli | 0.53 | 2.23 | 8.84 | 5.94 | 1.34 | 3.09 | 1.10 | 2.13 | 1.31 | 3.72 | 1.21 | 2.75 | 1.31 |
| Diadem | diadema | 0.27 | 1.57 | 6.44 | 4.95 | 1.62 | 3.29 | 1.19 | 2.01 | 1.07 | 2.76 | 1.10 | 2.55 | 1.26 |
| Diamond | diamante | 0.73 | 1.57 | 8.45 | 4.25 | 1.34 | 2.55 | 1.29 | 2.87 | 1.22 | 2.05 | 1.13 | 4.44 | 0.90 |
| Medal | medaglia | 0.07 | 3.25 | 9.84 | 4.42 | 1.14 | 3.55 | 1.03 | 3.20 | 1.15 | 2.64 | 1.14 | 2.91 | 1.25 |
| Necklace | collana | 0.94 | 0.45 | 9.76 | 2.57 | 1.14 | 2.37 | 0.84 | 4.22 | 1.03 | 3.22 | 1.14 | 4.66 | 0.67 |
| Pendant | orecchini | 0.61 | 1.91 | 7.50 | 3.15 | 1.27 | 1.87 | 0.84 | 4.27 | 1.18 | 3.93 | 1.13 | 4.36 | 0.86 |
| Ring | anello | 1.00 | 0.00 | 10.04 | 3.13 | 1.35 | 2.58 | 1.02 | 4.20 | 1.04 | 4.38 | 1.07 | 4.61 | 0.83 |
| Seal ring | anello con sigillo | 0.01 | 0.89 | 1.61 | 5.48 | 1.68 | 2.33 | 1.02 | 1.77 | 1.14 | 4.11 | 1.24 | 2.56 | 1.41 |
| Tie clip | fermacravatta | 0.09 | 2.45 | 2.71 | 6.08 | 1.29 | 2.63 | 1.04 | 1.67 | 0.92 | 3.67 | 1.13 | 2.08 | 1.18 |
| **Kitchen Utensils** |  |  |  |  |  |  |  |  |  |  |  |  |  |  |
| Cooking pot | pentola da cottura | 0.00 | 3.29 | 0.00 | 5.47 | 1.48 | 2.08 | 0.94 | 2.60 | 1.37 | 3.91 | 1.05 | 3.11 | 1.21 |
| Cup | tazza | 0.70 | 1.44 | 8.84 | 2.10 | 1.05 | 1.70 | 0.81 | 4.52 | 1.02 | 4.45 | 0.74 | 3.79 | 1.10 |
| Fondue | fonduta | 0.15 | 2.97 | 6.24 | 6.41 | 1.14 | 3.83 | 1.03 | 1.91 | 1.12 | 4.25 | 0.93 | 2.08 | 0.87 |
| Fork | forchetta | 1.00 | 0.00 | 8.18 | 1.92 | 0.85 | 1.82 | 0.97 | 4.93 | 0.30 | 4.82 | 0.66 | 4.75 | 0.53 |
| Frying pan | padella | 0.83 | 0.85 | 9.02 | 3.35 | 1.18 | 1.83 | 0.75 | 4.59 | 0.74 | 4.28 | 0.93 | 4.70 | 0.54 |
| Peeler | pelapatate | 0.36 | 2.46 | 4.26 | 5.41 | 1.33 | 2.51 | 0.95 | 3.07 | 1.27 | 4.67 | 0.72 | 3.03 | 1.27 |
| Pot | pentola | 0.93 | 0.48 | 8.93 | 2.89 | 1.12 | 1.91 | 0.86 | 4.68 | 0.65 | 4.11 | 0.94 | 4.68 | 0.68 |
| Saucepan | casseruola | 0.03 | 0.72 | 8.26 | 5.35 | 1.72 | 1.88 | 0.72 | 3.45 | 1.40 | 4.01 | 0.91 | 3.69 | 1.09 |
| Sharpening steel | affilacoltelli | 0.01 | 3.50 | 0.69 | 6.04 | 1.28 | 1.84 | 0.81 | 1.85 | 1.16 | 4.37 | 1.02 | 2.10 | 1.08 |
| Small saucepan | pentolino | 0.22 | 1.79 | 6.93 | 3.75 | 1.33 | 1.94 | 0.93 | 4.44 | 0.87 | 4.39 | 0.87 | 4.31 | 0.86 |
| Strainer | colino | 0.24 | 2.95 | 6.48 | 5.20 | 1.48 | 2.44 | 0.95 | 3.55 | 1.13 | 4.31 | 0.77 | 3.36 | 1.18 |
| Teapot | teiera | 0.84 | 0.90 | 5.91 | 4.80 | 1.45 | 2.82 | 1.05 | 3.54 | 1.14 | 4.21 | 0.96 | 3.62 | 1.09 |
| **Musical Instruments** |  |  |  |  |  |  |  |  |  |  |  |  |  |  |
| Accordion | fisarmonica | 0.89 | 0.61 | 8.04 | 4.56 | 1.27 | 3.51 | 1.17 | 2.70 | 1.19 | 4.96 | 0.19 | 3.43 | 1.20 |
| Balalaika | balalaika | 0.03 | 2.42 | 3.76 | 6.79 | 0.60 | 4.29 | 0.91 | 1.29 | 0.87 | 4.77 | 0.71 | 2.31 | 1.44 |
| Bugle | tromba | 0.80 | 1.12 | 8.84 | 4.06 | 1.33 | 2.26 | 0.82 | 2.59 | 1.28 | 4.20 | 1.01 | 4.16 | 1.03 |
| Clarinet | clarinetto | 0.52 | 2.26 | 7.74 | 5.54 | 1.26 | 3.78 | 1.24 | 2.26 | 1.21 | 4.74 | 0.77 | 3.51 | 1.18 |
| Drum | tamburo | 0.87 | 0.89 | 8.37 | 2.99 | 1.32 | 3.00 | 1.08 | 3.03 | 1.42 | 4.79 | 0.55 | 4.07 | 0.86 |
| Flute | flauto | 0.89 | 0.49 | 8.58 | 4.14 | 1.25 | 1.81 | 0.82 | 3.38 | 1.25 | 4.74 | 0.73 | 4.13 | 1.03 |
| Guitar | chitarra | 0.76 | 1.02 | 10.34 | 3.10 | 1.31 | 2.84 | 1.02 | 3.88 | 1.15 | 4.95 | 0.22 | 4.83 | 0.60 |
| Harmonica | armonica | 0.29 | 2.19 | 8.56 | 4.87 | 1.54 | 2.73 | 0.84 | 2.37 | 1.31 | 4.39 | 0.88 | 2.89 | 1.19 |
| Harp | arpa | 0.93 | 0.53 | 7.81 | 5.00 | 1.30 | 3.38 | 1.15 | 2.42 | 1.34 | 4.64 | 1.01 | 3.76 | 1.28 |
| Maracas | maracas | 0.79 | 1.14 | 5.48 | 4.40 | 1.62 | 2.84 | 0.92 | 2.29 | 1.21 | 4.74 | 0.52 | 2.78 | 1.19 |
| Piano | pianoforte | 0.76 | 1.02 | 9.66 | 3.44 | 1.37 | 3.95 | 1.09 | 3.53 | 1.33 | 4.95 | 0.28 | 4.81 | 0.48 |
| Saxophone | sassofono | 0.41 | 2.34 | 6.95 | 5.64 | 1.23 | 4.16 | 1.17 | 2.27 | 1.19 | 4.67 | 0.97 | 4.06 | 1.05 |
| Tambourine | tamburello | 0.52 | 2.36 | 6.39 | 3.39 | 1.49 | 3.52 | 1.18 | 2.46 | 1.34 | 4.67 | 0.69 | 2.62 | 1.30 |
| Trumpet | tromba | 0.96 | 0.28 | 8.84 | 3.89 | 1.27 | 3.48 | 1.23 | 2.76 | 1.30 | 4.80 | 0.49 | 4.47 | 0.68 |
| Tuba | tuba | 0.04 | 2.20 | 6.79 | 5.91 | 1.33 | 3.55 | 1.18 | 2.06 | 1.11 | 4.49 | 1.00 | 3.79 | 1.33 |
| Violin | violino | 0.85 | 0.86 | 9.17 | 3.93 | 1.24 | 3.52 | 1.17 | 3.20 | 1.21 | 4.84 | 0.58 | 4.40 | 0.84 |
| **Sport/Games** |  |  |  |  |  |  |  |  |  |  |  |  |  |  |
| Ball | palla | 0.80 | 0.86 | 10.55 | 1.48 | 0.70 | 2.61 | 1.30 | 4.21 | 1.02 | 4.13 | 1.10 | 4.53 | 0.77 |
| Chess | scacchi | 0.35 | 1.49 | 9.17 | 3.89 | 1.30 | 3.66 | 1.27 | 3.26 | 1.29 | 4.67 | 0.85 | 3.12 | 1.13 |
| Dart | freccetta | 0.83 | 0.99 | 5.65 | 4.31 | 1.35 | 2.47 | 0.92 | 2.65 | 1.26 | 4.57 | 0.79 | 2.93 | 1.14 |
| Dartboard | bersaglio | 0.27 | 2.57 | 9.57 | 4.74 | 1.34 | 3.74 | 1.12 | 2.64 | 1.19 | 3.56 | 1.41 | 3.11 | 1.13 |
| Diabolo | diabolo | 0.00 | 2.06 | 3.89 | 6.21 | 1.25 | 2.89 | 1.09 | 1.56 | 0.90 | 4.59 | 0.86 | 2.06 | 1.20 |
| Dice | dado | 0.99 | 0.10 | 7.91 | 2.48 | 1.16 | 1.81 | 0.80 | 3.69 | 1.29 | 4.53 | 0.78 | 3.33 | 1.26 |
| Doll | bambola | 1.00 | 0.00 | 8.19 | 1.61 | 0.74 | 3.27 | 1.27 | 3.56 | 1.29 | 3.85 | 1.04 | 3.85 | 1.20 |
| Jump rope | corda per saltare | 0.49 | 1.57 | 2.71 | 3.24 | 1.33 | 2.59 | 0.94 | 3.12 | 1.36 | 4.70 | 0.66 | 3.07 | 1.18 |
| Ludo | gioco da tavolo | 0.15 | 2.95 | 5.93 | 3.80 | 1.43 | 4.24 | 0.98 | 3.37 | 1.18 | 4.60 | 0.74 | 3.77 | 1.17 |
| Racket | racchetta | 0.66 | 1.00 | 6.68 | 3.75 | 1.15 | 2.47 | 0.89 | 3.33 | 1.35 | 4.78 | 0.71 | 4.04 | 1.06 |
| Skate | pattino | 0.43 | 2.11 | 5.78 | 3.46 | 1.22 | 3.20 | 1.17 | 2.90 | 1.26 | 2.44 | 1.25 | 3.04 | 1.16 |
| Ski | sci | 1.00 | 0.00 | 9.96 | 3.52 | 1.33 | 2.04 | 0.88 | 2.91 | 1.35 | 2.41 | 1.07 | 3.51 | 1.11 |
| Skittle | birillo | 0.96 | 0.28 | 5.24 | 4.14 | 1.42 | 1.88 | 0.85 | 3.08 | 1.36 | 2.52 | 1.35 | 3.29 | 1.16 |
| Soccer ball | pallone da calcio | 0.32 | 1.25 | 4.91 | 2.24 | 1.17 | 2.01 | 0.98 | 3.71 | 1.33 | 2.53 | 1.07 | 4.72 | 0.63 |
| Spinning top | trottola | 0.71 | 1.52 | 6.74 | 3.32 | 1.52 | 2.46 | 0.91 | 2.32 | 1.21 | 4.15 | 1.09 | 2.24 | 1.11 |
| Table football | calcio balilla | 0.23 | 2.42 | 5.21 | 4.67 | 1.43 | 3.84 | 1.15 | 3.36 | 1.33 | 4.91 | 0.50 | 3.61 | 1.06 |
| **Tools** |  |  |  |  |  |  |  |  |  |  |  |  |  |  |
| Axe | ascia | 0.39 | 1.97 | 7.91 | 4.53 | 1.36 | 2.14 | 0.87 | 2.51 | 1.32 | 4.65 | 0.80 | 3.45 | 1.15 |
| Bit | punta da trapano | 0.09 | 3.12 | 3.22 | 5.67 | 1.32 | 2.16 | 1.05 | 2.27 | 1.36 | 3.37 | 1.29 | 2.73 | 1.27 |
| Chisel | cesello | 0.00 | 2.62 | 5.93 | 6.56 | 1.07 | 2.28 | 0.99 | 1.52 | 1.02 | 4.47 | 0.95 | 2.63 | 1.16 |
| Cold chisel | scalpello | 0.18 | 2.78 | 6.58 | 5.62 | 1.39 | 1.80 | 1.15 | 1.79 | 1.08 | 4.35 | 1.17 | 2.86 | 1.21 |
| Hammer | martello | 0.98 | 0.17 | 8.72 | 3.12 | 1.40 | 1.87 | 0.73 | 3.41 | 1.29 | 4.56 | 0.79 | 4.57 | 0.79 |
| Handsaw | sega | 0.92 | 0.52 | 8.02 | 4.07 | 1.46 | 1.56 | 0.64 | 2.56 | 1.37 | 4.63 | 0.87 | 4.03 | 1.07 |
| Leveller | livella | 0.11 | 2.63 | 6.07 | 6.29 | 1.19 | 2.99 | 1.14 | 2.15 | 1.33 | 4.01 | 1.25 | 2.56 | 1.19 |
| Nail (tool) | chiodo | 0.90 | 0.67 | 8.43 | 3.27 | 1.41 | 1.32 | 0.61 | 3.23 | 1.34 | 3.72 | 1.13 | 3.62 | 1.22 |
| Nut | dado | 0.09 | 1.63 | 7.91 | 5.35 | 1.50 | 2.05 | 1.00 | 2.47 | 1.20 | 3.92 | 1.02 | 2.87 | 1.28 |
| Pincers | pinze | 0.08 | 1.90 | 7.79 | 4.74 | 1.48 | 2.36 | 0.90 | 2.94 | 1.19 | 4.67 | 0.78 | 4.22 | 0.97 |
| Pliers | tenaglie | 0.06 | 2.92 | 5.73 | 5.16 | 1.36 | 1.87 | 0.73 | 2.49 | 1.16 | 4.72 | 0.55 | 3.59 | 1.11 |
| Screw | vite | 0.61 | 1.62 | 10.55 | 4.04 | 1.40 | 2.33 | 1.09 | 2.77 | 1.26 | 4.08 | 0.93 | 3.21 | 1.17 |
| Screwdriver | cacciavite | 0.88 | 0.77 | 6.86 | 4.33 | 1.37 | 1.79 | 0.80 | 3.28 | 1.35 | 4.78 | 0.68 | 4.20 | 1.01 |
| Shovel | pala | 0.49 | 2.02 | 8.25 | 3.48 | 1.27 | 2.01 | 0.78 | 2.89 | 1.33 | 4.57 | 0.77 | 3.79 | 1.09 |
| Trowel | cazzuola | 0.07 | 2.83 | 5.79 | 6.05 | 1.26 | 2.32 | 0.97 | 1.64 | 0.91 | 4.52 | 1.03 | 2.97 | 1.25 |
| **Vehicles** |  |  |  |  |  |  |  |  |  |  |  |  |  |  |
| Boat | barca | 0.80 | 1.25 | 10.72 | 2.69 | 1.24 | 2.65 | 1.10 | 3.20 | 1.34 | 3.49 | 1.14 | 2.71 | 1.25 |
| Bus | autobus | 0.27 | 1.90 | 10.44 | 3.55 | 1.38 | 2.76 | 1.11 | 4.45 | 1.03 | 3.06 | 1.44 | 3.98 | 0.98 |
| Car | automobile | 0.49 | 2.41 | 9.75 | 2.57 | 1.29 | 3.13 | 1.30 | 4.77 | 0.76 | 4.80 | 0.52 | 4.83 | 0.69 |
| Cart | carro | 0.33 | 2.07 | 9.46 | 3.87 | 1.45 | 3.66 | 1.04 | 2.60 | 1.29 | 3.40 | 1.35 | 2.33 | 1.19 |
| Motorbike | moto | 0.66 | 1.53 | 11.30 | 3.05 | 1.41 | 3.79 | 1.39 | 3.99 | 1.14 | 4.54 | 0.79 | 4.30 | 0.81 |
| Paragliding | parapendio | 0.11 | 2.31 | 6.74 | 5.52 | 1.27 | 3.01 | 1.18 | 1.97 | 1.01 | 3.77 | 1.25 | 1.55 | 0.96 |
| Plane | aereo | 0.78 | 1.08 | 10.93 | 2.81 | 1.30 | 3.00 | 1.21 | 4.04 | 0.91 | 2.52 | 1.44 | 4.21 | 0.94 |
| Scooter | monopattino | 0.98 | 0.17 | 5.84 | 3.70 | 1.21 | 2.45 | 0.94 | 2.90 | 1.35 | 4.23 | 0.89 | 2.01 | 1.19 |
| Ship | nave | 0.03 | 2.12 | 10.63 | 2.84 | 1.29 | 3.48 | 1.02 | 3.08 | 1.31 | 3.41 | 1.19 | 3.21 | 1.17 |
| Skateboard | skateboard | 0.93 | 0.37 | 6.47 | 4.79 | 1.40 | 1.86 | 0.87 | 2.88 | 1.30 | 2.44 | 1.18 | 1.75 | 1.10 |
| Tractor | trattore | 1.00 | 0.00 | 7.59 | 2.91 | 1.41 | 3.87 | 1.30 | 3.29 | 1.30 | 4.10 | 1.13 | 3.04 | 1.17 |
| Train | treno | 0.95 | 0.38 | 10.77 | 2.97 | 1.55 | 3.52 | 1.17 | 4.25 | 1.19 | 2.81 | 1.27 | 4.17 | 0.81 |
| Van | furgone | 0.74 | 1.38 | 8.49 | 3.91 | 1.13 | 3.20 | 1.20 | 3.86 | 1.17 | 4.34 | 1.17 | 4.13 | 1.06 |
| **Weapons** |  |  |  |  |  |  |  |  |  |  |  |  |  |  |
| Armour | armatura | 0.89 | 0.81 | 8.69 | 4.17 | 1.32 | 3.60 | 1.20 | 2.28 | 1.32 | 3.30 | 1.20 | 2.98 | 1.28 |
| Arrow | freccia | 0.96 | 0.31 | 9.21 | 3.04 | 1.25 | 1.37 | 0.61 | 2.41 | 1.28 | 4.11 | 1.07 | 2.91 | 1.32 |
| Bayonet | baionetta | 0.12 | 1.37 | 6.87 | 6.47 | 1.00 | 2.12 | 1.30 | 1.35 | 0.74 | 3.13 | 1.41 | 2.28 | 1.17 |
| Boomerang | boomerang | 0.66 | 1.90 | 7.84 | 4.57 | 1.41 | 1.87 | 1.15 | 2.21 | 1.25 | 4.49 | 0.98 | 1.66 | 0.96 |
| Bow | arco | 0.99 | 0.10 | 11.06 | 3.13 | 1.21 | 1.57 | 0.66 | 2.52 | 1.26 | 4.57 | 0.72 | 2.83 | 1.23 |
| Cannon | cannone | 0.95 | 0.35 | 8.45 | 3.94 | 1.37 | 2.93 | 1.03 | 2.34 | 1.30 | 3.61 | 1.24 | 3.79 | 1.20 |
| Crossbow | balestra | 0.38 | 2.43 | 7.14 | 5.72 | 1.33 | 3.06 | 1.05 | 1.68 | 1.00 | 4.71 | 0.56 | 2.73 | 1.36 |
| Grenade | granata | 0.21 | 2.34 | 8.34 | 5.68 | 1.48 | 2.77 | 0.94 | 1.77 | 1.11 | 4.19 | 1.00 | 3.67 | 1.35 |
| Gun | pistola | 0.87 | 0.86 | 9.71 | 3.86 | 1.43 | 2.67 | 0.92 | 2.47 | 1.42 | 4.72 | 0.73 | 4.73 | 0.81 |
| Helmet | elmo | 0.27 | 2.58 | 7.73 | 4.50 | 1.36 | 2.05 | 0.72 | 2.09 | 1.31 | 2.61 | 1.09 | 2.26 | 1.13 |
| Machine gun | mitragliatrice | 0.13 | 2.59 | 7.13 | 5.36 | 1.25 | 3.49 | 1.03 | 1.80 | 1.20 | 4.63 | 0.77 | 4.16 | 1.09 |
| Revolver | rivoltella | 0.00 | 0.46 | 6.68 | 5.64 | 1.53 | 2.92 | 1.00 | 1.85 | 1.02 | 4.53 | 1.04 | 4.36 | 1.11 |
| Shield | scudo | 0.88 | 0.80 | 9.27 | 3.85 | 1.47 | 3.49 | 1.23 | 2.32 | 1.32 | 3.87 | 1.18 | 3.02 | 1.25 |
| Slingshot | fionda | 0.84 | 0.92 | 6.92 | 3.71 | 1.32 | 2.83 | 0.92 | 2.21 | 1.20 | 4.72 | 0.56 | 2.12 | 1.13 |
| Sword | spada | 0.95 | 0.34 | 10.25 | 2.99 | 1.19 | 1.71 | 0.78 | 2.51 | 1.44 | 4.52 | 0.95 | 4.07 | 1.01 |

Note. Agr-P = Percentage of name agreement; Agr-H= H statistic on name agreement; Freq = Lexical frequency (natural logarithm); AoA =Age of Acquisition; Vis Com = Visual Complexity; Fam = Familiarity; Man=Manipulability; Tip = tipicality
