# Supplementary material for: Italian norms and naming latencies for 357 high quality color images
Source: PLoS One. 2019 Feb 22;14(2):e0209524. doi: 10.1371/journal.pone.0209524 (PMC6386297; doi:10.1371/journal.pone.0209524)
Supplement: S2 Table — (DOCX) [file pone.0209524.s002.docx]

| **Item** | **Target name (Italian)** | **Alternative/synonyms name-s (Italian)** |
| --- | --- | --- |
| **Animals** |  |  |
| Armadillo | armadillo (27) | ermellino (2), formichiere (4), mandrillo (3), nc (11), nr (10), ornitorinco (1), paguro (1), pl (10), talpa (1), tapiro (4) |
| Bat | pipistrello (84) | nottolo (1) |
| Cat | gatto (79) | gatto comune (1), gatto europeo (1), nr (1) |
| Cheetah | ghepardo (30) | ghepardo delle nevi (1), giaguaro (3), iena (2), leopardo (14), lince (4), nc (3), nr (3), pantera (2), pl (7), puma (1), tigre (4), tigre siberiana (1) |
| Cow | mucca (80) | vacca (4), vitello (1) |
| Crocodile | coccodrillo (72) | alligatore (10) |
| Dromedary | dromedario (54) | cammello (20), lama (1) |
| Elephant | elefante (84) | elefante indiano (1) |
| Giraffe | giraffa (82) |  |
| Hippopotamus | ippopotamo (65) | nc (1), pl (6), rinoceronte (3) |
| Horse | cavallo (83) | pony (1) |
| Kangaroo | canguro (82) |  |
| Lioness | leonessa (45) | leone (24), leopardo (1), pl (1), tigre (4) |
| Lynx | lince (41) | felino (1), gatto (9), gattopardo (2), ghepardo (10), iena (1), leopardo (3), nc (4), nr (3), ocelot (1), pantera (1), pl (8), tigre (1) |
| Platypus | ornitorinco (36) | castoro (1), nc (21), nr (10), pl (10), quadrupede (1), talpa (1), tapiro (1), tasso (1) |
| Rhino | rinoceronte (72) | ippopotamo (1), nc (1), pl (1) |
| Snake | serpente (76) | anaconda (2), biscia (1), serpe (1), serpente boa (1), vipera (4) |
| Tapir | tapiro (23) | armadillo (1), formichiere (11), nc (13), nr (16), ornitorinco (3), pl (12), rinoceronte (2), tasso (1) |
| Tiger | tigre (73) | leone (1), pl (1) |
| Turtle | tartaruga (78) | nr (1), tartaruga di mare (1), tartaruga di terra (3), testuggine (2) |
| Zebra | zebra (81) | nr (1) |
| **Birds** |  |  |
| Barn owl | barbagianni (8) | civetta (46), gufo (20), nr (1) |
| Duck | anatra (54) | anatra selvatica (1), anitra (1), germano reale (2), nc (1), nr (1), oca (6), papera (17), papero (1), uccello (1) |
| Goldfinch | No Response | canarino (4), colibrì (1), fringuello (2), nc (37), nr (9), pappagallo (1), passero (5), passerotto (2), pettirosso (4), picchio (1), pl (3), uccellino (3), uccello (8) |
| Goose | oca (63) | anatra (7), cigno (1), papera (4) |
| Hen | gallina (77) | gallo (6), pollo (2) |
| Hummingbird | colibrì (37) | nc (23), nr (7), pavone (1), pettirosso (1), picchio (1), pl (1), uccello (10) |
| Kiwi | kiwi (10) | dodo (5), em√π (1), gnu (1), ibis (1), nc (34), nr (12), pl (7), struzzo (2), uccello (2) |
| Magpie | gazza (13) | canarino (1), corvo (2), fringuello (1), gazza ladra (15), gazzaladra (6), gazzella (1), merlo (5), nc (13), nr (13), passerotto (1), pl (5), uccello (9) |
| Ostrich | struzzo (71) | gru (1), nc (1), nr (2), oca (1), pavone (1), pl (1), poiana (1), struzzo maschio (1), tacchino (2) |
| Owl | gufo (63) | aquila (1), civetta (9), nc (1), pl (1) |
| Partridge | pernice (1) | anitra (1), fagiano (2), falco (1), faraona (1), ghiandaia (1), nc (19), nr (10), piccione (26), pl (11), quaglia (1), tortora (4), uccello (6), uncorrect alternative response (1) |
| Pelican | pellicano (41) | albatros (1), cicogna (4), fagiano (1), gabbiano (2), nc (7), nr (7), pavone (1), pl (15), stambecco (1), tucano (2) |
| Penguin | pinguino (73) | pinguino imperatore (1), pinguino reale (1) |
| Pheasant | fagiano (30) | faraona (1), nc (24), nr (5), pavone (13), pavone femmina (2), pernice (1), pl (3), quaglia (4), uccello (1), volatile (1) |
| Pigeon | piccione (72) | colomba (2), colombo (6), passero (1), pettirosso (1) |
| Raven | corvo (58) | cornacchia (1), corvo nero (1), falco (2), gazza (1), gazza ladra (1), merlo (5), nc (2), pl (3), uccello (1) |
| Rooster | gallo (74) | gallina (4), gallo cedrone (1), pl (2), pollo (4) |
| Seagull | gabbiano (73) | nc (2), nr (1), pl (4), uccello (2) |
| Sparrow | passero (31) | allodola (1), fringuello (2), nc (5), nr (3), passerotto (13), pettirosso (9), uccellino (2), uccello (9) |
| Toucan | tucano (39) | dodo (1), merlo (2), nc (5), nr (8), pappagallo (11), pellicano (7), pennuto (1), pl (10), uccello (1) |
| **Body Parts** |  |  |
| Arm | braccio (61) | arto (1), avambraccio (16), braccio sinistro (3), pugno (1) |
| Beard | barba (15) | baffi (1), nc (55), nr (2), peli (1) |
| Bone | osso (79) | femore (5), pipistrello (1) |
| Brain | cervello (70) | cervello umano (2), emisfero sinistro (1), encefalo (9) |
| Ear | orecchio (74) | padiglione (1) |
| Eye | occhio (83) | bulbo oculare (1) |
| Finger | dito (21) | dito indice (14), indice (47) |
| Foot | piede (74) | nc (1) |
| Hand | mano (77) | mano femminile (3), mano sinistra (5) |
| Kidney | rene (20) | fagiolo (21), fegato (5), nc (22), nr (2), organo interno (1), patata (1), patata americana (1), pl (1), polmone (6), polmone destro (1), tubero (1) |
| Leg | gamba (72) | arto (1), arto inferiore (1), polpaccio (1) |
| Liver | fegato (40) | brioche (1), carne (6), cuore (5), interiora (1), nc (19), nr (6), organo (2), pl (3), polmone (2) |
| Lung | polmone (21) | bistecca (1), carne (1), cuore (3), fegato (12), lingua (2), nc (23), nr (10), organo (1), pelle (1), pietra (3), pl (1), rene (2), stomaco (1) |
| Mouth | bocca (40) | labbra (35) |
| Nail (bodypart) | unghia (76) | nc (6), nr (2), pl (1) |
| Nose | naso (85) |  |
| Pelvis | bacino (30) | anche (3), bacino osseo (1), cinto pelvico (1), cintura pelvica (1), coccige (2), femore (1), nc (4), nr (6), ossa (5), ossa del bacino (9), ossa dell'anca (1), ossa di bacino (1), ossa toraciche (1), osso (3), osso lombare (1), osso pelvico (2), osso pubico (1), osso sacro (3), pelvi (1), pl (2), pube (1), scheletro (2) |
| Skull | cranio (13) | scheletro (2), teschio (60) |
| Tongue | lingua (72) | lingua umana (2), nc (6), nr (2) |
| Vertebra | vertebra (6) | incudine (1), nc (57), nr (5), ossa (1), osso (2), pestello (2), reperto (1) |
| **Flowers** |  |  |
| Araceae | calla (32) | fiore (7), giglio (3), gladiolo (1), nc (9), nr (10), orchidea (3), pl (16), tulipano (3) |
| Bellflower | campanule (9) | campana (1), campanelle (4), campanelline (2), campanula (4), fiore (4), fiori (2), giacinto (1), giglio (2), glicine (1), mughetto (1), nc (14), nr (20), pl (7), tulipani (1), viole (5), violette (4) |
| Carnation | garofano (11) | crisantemo (2), fiore (14), fiori (1), geranio (2), mazzo di fiori (1), nc (13), nr (19), papavero (1), pl (7), rododendro (1), rosa (3) |
| Daisy | margherita (84) | fiore di margherita (1) |
| Geranium | geranio (5) | bouquet (2), bouquet di fiori (1), fiori (18), garofano (1), mazzo di fiori (2), nc (31), nr (13), orchidea (1), pl (4), rose (4) |
| Lilac | lilla (4) | fiore di ortensia (1), fiori (2), frangipane (1), glicine (13), ibiscus (1), lavanda (6), lilla (1), nc (15), nr (9), orchidea (2), ortensia (1), pl (11), viole (6), violette (2) |
| Orchid | orchidea (24) | achillea (1), fiore (18), foglia (1), lilla (1), nc (20), nr (12), pl (6), primula (1), viola (1) |
| Pansy | No Response | bocca di leone (1), ciclamino (2), fiore (14), fiore di vetro (1), nc (15), nr (14), orchidea (4), petali (1), petunia (2), pl (4), primula (10), viola (6), viola del pensiero (3), violetta (5) |
| Poppy | papavero (60) | fiore (1), nc (1), nr (2), papavero rosso (1), pl (1), polline (1), tulipano (8) |
| Rose | rosa (82) | nr (2), pl (1) |
| Sunflower | girasole (82) |  |
| Tulip | tulipano (54) | fiore (1), garofano (2), nc (3), nr (1), papavero (12), pl (2) |
| **Fruits** |  |  |
| Apple | mela (81) | fragola (1), mela rossa (2) |
| Avocado | avocado (10) | cedro (1), mango (1), melanzana (1), nc (45), nr (6), papaya (4), pl (7), prugna (5), prugna secca (1), zucca (1) |
| Banana | banana (75) |  |
| Cherries | ciliegie (84) | duroni (1) |
| Coconut | cocco (29) | mandorla (1), nc (6), noce (6), noce da cocco (1), noce di cocco (34), nr (2), pl (2) |
| Custard apple | No Response | avocado (2), carciofo (4), cavolo (1), mango (2), nc (55), nr (7), pl (3), pomello (1) |
| Fig | fico (64) | cipolla (1), fico d'india (3), fiorone (1), nc (6), nr (5), pl (5) |
| Flat peach | pesca tabacchiera (3) | caco (1), mela (4), nc (14), nr (5), pesca (46), pesca bianca (2), pesca gialla (1), pesca noce (2), pesca schiacciata (1), pl (3) |
| Grapes | uva (59) | grappolo (1), grappolo d'uva (2), grappolo di uva (2), uva fragola (1), uva nera (7), uva rossa (3) |
| Kiwi fruit | kiwi (83) | melone (1), nc (1) |
| Lemon | limone (82) |  |
| Mango | mango (9) | avocado (2), giuggiola (1), melanzana (2), nc (35), nr (16), papaya (4), pl (2), prugna (2), rapanello (1), susina (1) |
| Melon | melone (8) | anguria (31), carosello (1), cocomero (9), mango (5), nc (14), noce di cocco (1), nr (5), papaya (2), pl (7), zucca (2) |
| Orange | arancia (77) | arancio (2), mandarino (3) |
| Peach | pesca (24) | albicocca (5), mela (6), nc (16), nr (4), pl (10), prugna (1), prugna gialla (1), spugna (1), susina (5), uncorrect alternative response (2) |
| Pear | pera (85) |  |
| Pomegranate | melagrana (5) | caco (2), cipolla (1), melograna (4), melograno (64), nc (3), nr (1), pl (2) |
| Quince | mela cotogna (1) | cedro (1), frutto (1), limone (3), mango (1), mela (26), nc (24), nr (10), peperone (1), pesca (4), pl (3) |
| Redcurrant | ribes (39) | amarena (1), bacche (6), bacche rosse (1), cranberry (1), datterini (1), frutti di bosco (1), frutti rossi (1), iris (1), mirtilli (5), mirtilli rossi (1), nc (5), nr (5), pl (11), pomodori (2), pomodorini (1), ribes rosso (2), vischio (1) |
| Strawberry | fragola (82) |  |
| Watermelon | anguria (65) | cocomero (10) |
| **Insects** |  |  |
| Ant | formica (78) | insetto (1), nc (2), nr (1), pl (2), ragno (1) |
| Bee | ape (44) | calabrone (6), maggiolino (1), mosca (11), moscone (2), nc (5), nr (6), pl (1), vespa (6) |
| Beetle | scarabeo (33) | blatta (1), cimice (1), coleottero (1), maggiolino (1), nc (1), nr (3), pl (6), scarafaggio (26), stercorario (1), uncorrect alternative response (1) |
| Butterfly | farfalla (84) | nr (1) |
| Centipede | millepiedi (37) | centopiedi (2), insetto (2), lombrico (1), nc (21), nr (7), pl (3), scolopendra (6), scorpione (3) |
| Cockroach | scarafaggio (37) | blatta (1), cicala (1), coleottero (1), grillo (3), insetto (4), nc (16), nr (9), pl (3) |
| Dragonfly | libellula (55) | allodola (1), falena (5), farfalla (10), nc (5), nr (4), pl (5) |
| Fly | mosca (76) | ape (2), nc (1), nr (1), vespa (1), zanzara (1) |
| Grasshopper | cavalletta (54) | falena (1), grillo (7), insetto (1), locusta (2), nc (7), nr (3) |
| Ladybird | coccinella (85) |  |
| Mosquito | zanzara (60) | falena (1), libellula (4), nc (7), nr (7), pl (1), zampirone (1), zanzara di stagno (1) |
| Moth | tarma (1) | cimice (2), falena (32), farfalla (6), farfalla notturna (1), farfallina (1), insetto (2), nc (18), nr (5), pidocchio (1), pl (5) |
| Praying mantis | mantide religiosa (35) | cavalletta (11), grillo (5), insetto (3), locusta (1), mantide (13), nc (5), nr (5), pl (6), stercorario (1) |
| Scorpion | scorpione (74) | nc (1), nr (3), pl (3), tarantola (1) |
| Spider | ragno (51) | pl (4), tarantola (20) |
| Termite | termite (1) | acaro (1), cimice (2), formica (10), formica rossa (1), insetto (12), larva (2), nc (36), nr (8), pidocchio (1), pl (4), scarafaggio (4) |
| Wasp | vespa (38) | ape (42), nr (1), pl (1) |
| **Marine Creatures** |  |  |
| Cockle | conchiglia (67) | ostrica (1), pl (1), vongola (6) |
| Crab | granchio (80) | cancro (1), nc (1), pl (1), scorpione (2) |
| Dolphin | delfino (78) | nc (1), pesce spada (2), pl (1) |
| Eel | anguilla (31) | biscia (2), fagiolino (1), murena (7), nc (19), nr (3), pesce (1), pl (5), sanguisuga (2), serpente (2), uncorrect alternative response (1), verme (1) |
| Goose barnacle | No Response | corallo (1), mollusco (1), nc (72), nr (4), pl (4), scultura (1), statuetta (1), tubero (1) |
| Killer whale | orca (58) | balena (16), balenottera (1), delfino (2), foca (1), orca assassina (3), orca marina (1) |
| Lobster | aragosta (44) | astice (10), calamaro (1), gamberone (3), granchio (7), nc (1), nr (3), pl (4), scampo (1), scorpione (1) |
| Manatee | lamantino (4) | balena (2), beluga (1), dugongo (6), elefante marino (4), foca (26), leone marino (12), lupo marino (1), mammifero marino (1), nc (1), nr (12), otaria (1), pl (9), tricheco (5) |
| Mussel | cozza (70) | conchiglia (1), nc (1), ostrica (3), pl (1), vongola (6) |
| Narwhal | narvalo (5) | balena (6), n (1), nc (26), nr (16), orca (2), pesce spada (7), pescespada (2), pl (9) |
| Oyster | ostrica (14) | ambra (1), carne (1), conchiglia (5), fico (1), fossile (2), fungo (1), guscio (1), lumaca (3), mollusco (3), nc (44), nr (5), pl (1), uva passa (1), vongola (2) |
| Pomfret | No Response | bronzino (1), carpa (2), nc (35), nr (7), orata (2), pesce (23), pesce palla (1), pirana (2), pl (3), scorfano (1), sgombro (1), spigola (1), tonno (1), trota (2) |
| Ray | razza (25) | gazza (1), manta (9), mantide (1), mantina (1), nc (9), nr (8), pl (16), rana pescatrice (1), rombo (3), sogliola (1) |
| Razor-shell | cannolicchio (2) | canocchia (2), cappalonga (1), fasolare (1), mollusco (3), nc (63), nr (7), pl (3), tellina (1), vongola (1) |
| Shark | squalo (44) | nc (14), nr (12), pesce (4), pesce spada (2), pesce squalo (1), pescecane (1), pl (2), squaletto (1), squalo balena (1) |
| Sperm whale | capodoglio (6) | balena (49), beluga (1), cetaceo (1), lamantino (1), megattera (2), nc (6), nr (3), pl (2), squalo (4) |
| Starfish | stella marina (85) |  |
| Whale | balena (27) | balenottera (1), capodoglio (2), delfino (1), nc (34), nr (12), pesce (2), pl (3) |
| **Nuts** |  |  |
| Almond | mandorla (35) | arachide (1), ghianda (2), guscio (1), nc (32), nocciola (2), nocciolo (2), noce (11), noce moscata (1), nr (7), seme della pesca (1) |
| Chestnut | castagna (59) | cipolla (15), cipolla rossa (2), marrone (1), nc (2), pl (2), radicchio (1) |
| Date | dattero (26) | melanzana (1), nc (23), nr (7), pl (2), prugna (8), prugna secca (4), uva passa (2), uva secca (1), uvetta (1) |
| Hazelnut | nocciola (35) | castagna (8), cipolla (1), cipolla rossa (1), ghianda (1), mandorla (2), nc (13), nocciolina (10), noce (1), nr (2), pl (3), zucca (7) |
| Peanut | arachide (48) | bagigi (1), bagigio (15), nc (1), nocciolina (12), nocciolina americana (3), noce (1), nr (1) |
| Pine kernel | pinolo (1) | ciottolo (1), dattero (1), nc (41), nocciola (1), nr (5), patata (18), pietra (1), pl (2), sasso (1), seme (1), tubero (2) |
| Pipe | seme (2) | balena (1), melanzana (2), nc (54), nr (11), pl (3), sasso (1), seme di girasole (9), seme di zucca (2) |
| Pistachio | pistacchio (59) | arachide (7), biscotto della fortuna (1), capasanta (1), nc (4), nocciolina (1), noce (2), nr (1), pl (6) |
| Raisin | uva passa (6) | albicocca secca (1), dattero (19), dattero essiccato (1), frutta secca (3), nc (16), nr (1), pl (5), prugna (4), prugna secca (3), uva secca (1), uvetta (11), uvetta essiccata (1), uvetta secca (1), uvetta sultanina (2) |
| Walnut | noce (85) |  |
| **Trees** |  |  |
| Black poplar | pioppo (5) | abete (6), albero (9), cipresso (23), nc (7), nr (5), pino (18), pl (8), salice (1) |
| Cedar | No Response | abete (28), albero (4), nr (1), pino (41), pl (1) |
| Cypress | cipresso (23) | abete (9), acero (1), albero (5), larice (1), nc (1), nr (1), pino (36), pioppo (1), pl (7) |
| Eucalyptus | No Response | albero (33), betulla (3), frassino (1), nc (20), nr (20), pianta (1), pino marittimo (1), pl (2), platano (1) |
| Fig tree | fico (3) | albero (31), arancio (3), magnolia (2), nc (18), nr (13), pesco (1), pioppo (1), pl (2), quercia (1) |
| Fir | abete (36) | albero (7), nc (2), nr (2), pino (33), pino silvestre (1), pl (3), sequoia (1) |
| Holm oak | No Response | abete (3), albero (19), albero sempreverde (1), baobab (2), nc (12), nr (13), olivo (1), pl (2), quercia (27), sequoia (1) |
| Olive tree | olivo (3) | albero (18), baobab (2), betulla (1), faggio (1), nc (7), nr (3), pl (3), quercia (8), salice (1), ulivo (28) |
| Palm tree | palma (82) | albero (1), pl (2) |
| Pine tree | pino (2) | albero (29), baobab (2), nc (17), nr (13), pino marittimo (8), pl (4), quercia (5), sempreverde (1) |
| Willow | salice (26) | albero (4), albero secolare (1), cipresso (1), felce (1), nc (2), nr (2), pl (5), quercia (1), salice piangente (31) |
| **Vegetables** |  |  |
| Artichoke | carciofo (79) | asparago (1), broccolo (1), cavolfiore (1), nr (1), pl (2) |
| Asparagus | asparago (64) | asparago verde (2), bruscandolo (2), canna (1), carciofo (3), cime di rapa (1), erba (1), nc (2), nr (4), pl (2) |
| Cabbage | cavolo (7) | cappuccio (6), cavolfiore (1), cavolo cappuccio (1), insalata (29), insalata iceberg (2), lattuga (20), nr (1), pl (2), verza (6) |
| Carrot | carota (84) | nc (1) |
| Cauliflower | cavolfiore (62) | broccolo (3), carciofo (1), cavolo (15), pl (1) |
| Celery | sedano (65) | costa (1), lattuga (1), nc (1), pl (3), porro (1), prezzemolo (2), verza (1) |
| Chard | bietola (7) | bieta (1), cicoria (1), coste (3), insalata (25), insalata gentile (1), invidia (1), lattuga (8), nc (3), nr (8), pl (16), radicchio (1), sedano (2), spinaci (3), uncorrect alternative response (1), verdura (1), verza (3) |
| Cucumber | cetriolo (71) | nc (2), pl (1), zucchina (8) |
| Eggplant | melanzana (73) | verza (1), zucchina (1) |
| Endive | No Response | cavolfiore (1), cavolo (1), inalata riccia (1), insalata (42), insalata gentile (1), insalata iceberg (1), insalata radicchio (1), insalata riccia (9), lattuga (18), lattuga riccia (1), nc (2), nr (3), pl (4) |
| Leek | porro (34) | aloe (1), carciofo (1), cavolo (1), cipolla (1), cipollina fresca (1), cipollotto (4), erba cipollina (1), finocchio (2), gambo di sedano (1), nc (5), nr (9), pl (7), prezzemolo (1), rapa (1), sedano (11) |
| Lettuce | lattuga (24) | bietole (1), cavolo (1), costa (1), insalata (34), insalata romana (1), insalata verde (1), nc (2), nr (5), pl (4), verza (1) |
| Onion | cipolla (77) | aglio (2), cipolla bianca (2), cipolla rossa (2), scalogno (1), zucca (1) |
| Pepper | peperone (67) | melanzana (1), peperone verde (14) |
| Potato | patata (74) | nr (1) |
| Pumpkin | zucca (63) | arachide (1), nc (7), nr (4), pl (7), tubero (1), zucca marina (1) |
| Red cabbage | cavolo rosso (1) | carciofo (1), cavolo (5), cavolo cappuccio (4), cavolo nero (1), cavolo viola (1), cipolla (3), crauto (1), fico (1), insalata rossa (1), melanzana (2), nc (42), nr (8), pl (1), radicchio (6), rapa rossa (1), verza (1), verza rossa (1) |
| Spinach | spinaci (28) | barbabietola (1), barbabietole (1), basilico (2), bieta (1), biete (2), bietola (2), bietole (1), erbette (1), insalata (8), lattuga (2), nc (4), nr (12), pl (7), rucola (1), valeriana (1), verdura (1) |
| Tomato | pomodoro (85) |  |
| Turnip | No Response | barbabietola (1), cetriolo (1), lime (1), mango (1), mela (1), nc (72), nr (1), patata (2), pera (2) |
| **Nature** |  |  |
| Cliff | scogliera (37) | costa (12), costa rocciosa (2), costiera (2), fiordi (2), golfo (1), isola (1), mare (2), montagne (5), nc (3), nr (2), pl (3), precipizio (1), promontorio (3), rocce (2), rocce costiere (1), spiaggia (1), strapiombo (2) |
| Cloud | nuvola (69) | nr (1), nuvole (5) |
| Coal | carbone (8) | antracite (1), felce (1), manganese (1), minerale (7), nc (26), nr (4), onice (1), opale (1), pietra (18), pietra pomice (1), pl (2), roccia (5), sasso (8), scoglio (1) |
| Gold | oro (13) | gheriglio (2), gheriglio di noce (1), minerale (2), nc (50), noce (9), nr (2), oro grezzo (1), sasso (1) |
| Ice | ghiaccio (15) | ghiacciaio (15), iceberg (36), nc (1), neve (1), nuvola (1), pl (5) |
| Iceberg | iceberg (73) | ghiacciaio (8), ghiaccio (1), nc (1), nr (2) |
| Island | isola (75) | atollo (1), duna (1), isoletta (1), isolotto (3), scoglio (1) |
| Moon | luna (63) | luna piena (9), nc (1), pianeta (2) |
| Mountain | montagna (70) | cima (1), cima della montagna (3), everest (1), montagna innevata (2), montagna rocciosa (1), monte (2), nc (1), pl (1), vetta (1), vetta di una montagna (1) |
| Puddle | pozzanghera (29) | corteccia (1), cotoletta impanata (1), laghetto (1), nc (38), nr (2), pelle (1), pozza (2), sabbia (4), tappeto (1), tavola di legno (1), terra (1) |
| Sea | mare (71) | acqua (4) |
| Stone | pietra (15) | fungo (1), marmo (1), masso (4), minerale (5), nc (17), nr (2), pane (1), pietra focaia (2), pl (2), quarzo (1), roccia (2), sasso (31), tartufo (1) |
| Sun | sole (78) | flash (1), luce (2) |
| Volcano | vulcano (73) | montagna (2) |
| Waterfall | cascata (68) | cascate (16), niagara (1) |
| Wave | onda (36) | bagnasciuga (2), mare (16), mare mosso (1), nr (1), onda che si infrange (1), onda marina (1), onde (13), onde del mare (3), onde marine (1), riva (1), spiaggia (5) |
| **Buildings** |  |  |
| Castle | castello (54) | castello medievale (1), fortezza (11), fortino (1), mura (1), mura del castello (2), nr (2), rocca (1), torre (1), torri (1) |
| Cathedral | cattedrale (38) | abbazia (1), basilica (3), castello (1), cattedrale medievale (1), chiesa (27), chiesa gotica (1), duomo (6), edificio (1), edificio gotico (1), notre dame (1), nr (3), pl (1) |
| Church | chiesa (56) | abbazia (1), campanile (17), cappella (1), cattedrale (1), chiesa con campanile (2), chiesa romanica (1), nc (1), pl (1), uncorrect alternative response (1) |
| Factory | fabbrica (37) | azienda (1), centrale (6), centrale nucleare (1), ciminiera (3), inceneritore (1), industria (11), nc (5), nr (5), pl (3), stabilimento industriale (1), struttura industriale (1) |
| Granary | No Response | aia (1), casa per pescatori (1), casa per uccelli (1), cascina (1), catafalco (1), monumento (1), nc (51), nr (13), palafitta (6), pl (2), tempio (1), tempio antico (1), tomba (4) |
| House | casa (68) | baita (2), casa di montagna (1), casetta (1), casolare (1), malga (1), nc (1), pl (2), villa (2), villetta (2), villino (1) |
| Lighthouse | faro (58) | faro marittimo (1), nc (2), nr (4), pl (2), torre (4), torre del porto (1), torre di controllo (2), torretta (1) |
| Mill | mulino (49) | molino (2), mulino a vento (28), nc (1), nr (2), pl (3) |
| Pagoda | pagoda (12) | casa cinese (1), edificio (1), monumento (1), muraglia cinese (1), nc (8), nr (17), pagoda cinese (1), palazzina cinese (1), palazzo (1), pl (9), tempio (13), tempio buddhista (1), tempio cinese (7), tempio giapponese (1), tempio orientale (1), templio (2), templio cinese (1), torre (2), ziggurat (1) |
| Palace | palazzo (13) | abbazia (3), castello (12), cattedrale (7), edificio (2), monastero (1), nc (10), nr (6), parlamento (1), pl (4), reggia (7), residenza reale (1), tenuta (1), villa (6) |
| Pyramid | piramide (77) | montagna di sabbia (2), nc (5), tetto (1) |
| Shanty | baracca (42) | bungalow (1), capanna (2), casa diroccata (1), casa in rovina (1), catapecchia (6), favelas (3), nc (15), nr (6), pl (4), ruderi (1) |
| Silo | silo (1) | cisterna (3), container (1), faro (2), nc (35), nr (7), pl (5), silos (18), torre (3) |
| Skyscraper | grattacielo (75) | palazzo (9), torri (1) |
| Tower | torre (72) | abbazia (1), campanile (2), fortino (1), mastio (1), nc (2), palazzo (1), torretta (1), torrione (1) |
| **Clothing** |  |  |
| Bathrobe | accappatoio (75) |  |
| Biretta | tocco (11) | berretto (1), cappello (14), cappello da diploma (4), cappello da laurea (4), cappello di laurea (11), cappello per laureandi (1), cappello per laureati (1), cappello universitario (1), nc (4), nr (15), pl (18) |
| Cap | cappello (38) | berretto (16), berretto con frontino (1), berretto con visiera (3), berretto sportivo (1), cappellino (13), cappellino da baseball (1), cappello con frontino (3), cappello con visiera (6) |
| Clog | zoccolo (29) | canoa (2), nc (34), nr (2), pl (2), scarpa (1), vaso (1), zoccolo olandese (3) |
| Coat | cappotto (77) | cappotto da donna (1), giacca (1), giaccone (2), pelliccia (3) |
| Glove | guanto (64) | guanti (2), guanto di pelle (8), guanto in pelle (8) |
| Jacket | giacca (68) | blazer (1), giacca da abito (1), giacca da uomo (3), giacca da vestito (1), giacca elegante (1) |
| Shirt | camicia (84) |  |
| Shoe | scarpa (68) | mocassino (4), pl (1), scarpa da uomo (4), scarpa di vernice (1), scarpa elegante (2), scarpe (2) |
| Skirt | gonna (72) | gonna a tubino (1), gonna a vita alta (1), gonna elegante (1) |
| Socks | calzini (49) | calza (3), calze (9), calzette (1), calzetti (1), calzino (18), nc (1), nr (1), paio di calzini (2) |
| Trousers | pantaloni (78) | pantaloni beige (1), pantaloni da uomo (1), pantaloni di tela (1), pantaloni maschili (1) |
| Undershirt | canottiera (59) | canotta (14), canottiera intima (2) |
| Desk Material |  |  |
| Compasses | compasso (83) | nr (1), pl (1) |
| Eraser | gomma (42) | chewingum (1), gomma da cancellare (17), gomma per cancellare (5), mattone (4), nc (10), sapone (2), spugna (1) |
| Felt-tip pen | pennarello (49) | indelebile (12), pennarello indelebile (9), pennarello per lavagna (2), pl (1), trattopen (2) |
| Folder | cartella (5) | agenda (1), carpetta (3), carpetta con elastico (1), carpetta di plastica (1), cartella portadocumenti (1), cartelletta (2), cartellina (24), contenitore (2), dispensa (1), faldone (1), formulario (1), nc (1), nr (4), pl (9), portacarte (1), portadocumenti (2), quaderno (2), quadernone (1), quadernone ad anelli (1), raccoglitore (19), teca (2) |
| Fountain pen | penna stilografica (44) | nc (2), nr (2), penna (8), penna a inchiostro (2), penna a stilo (1), penna stilo (3), pennino (1), pl (2), stilografica (17) |
| Ink pad | No Response | inchiostro (21), inchiostro a stampo (1), inchiostro per timbri (12), inchiostro per timbro (4), nc (5), nr (19), pl (6), spugna inchiostro (1), spugnetta di inchiostro (1), spugnetta per inchiostro (1), timbro (3) |
| Paperclip | graffetta (63) | clip (3), ferma carte (2), fermafogli (2), fermaglio (2), nr (1), pl (11), uncorrect alternative response (1) |
| Pen | penna (53) | bic (1), biro (6), penna a sfera (5), penna bic (1), penna biro (13), penna blu (1), penna stilo (1), stilo (1) |
| Pencil | matita (73) | lapis (1), pl (1) |
| Pencil sharpener | temperino (66) | appuntalapis (1), appuntamatite (1), temperalapis (1), temperamatite (16) |
| Rubber stamp | timbro (66) | nc (1), nr (2), pedina degli scacchi (1), pedone (2), pezzo degli scacchi (1), pl (3), pomello (1), stampo (4), timbrino (1) |
| Ruler | righello (77) | riga (5) |
| Set square | squadra (67) | squadretta (8) |
| Square ruler | squadra (77) | riga (1), righello (1), squadra geometrica (1), squadretta (5) |
| Stapler | No Response | bucafogli (1), campanello (1), cric (2), macchina per codice morse (1), nc (54), nr (9), pedale (1), pressa (1), rullante (1), spillatrice (1), telegrafo (2), uncorrect alternative response (1) |
| **Food** |  |  |
| Anchovy | acciughe (31) | alici (22), filetto di carne (1), filetto di pesce (1), nc (15), nr (4), pesce (1), pesce crudo (1), pl (3), sardine (5), seppia (1) |
| Black pudding | No Response | nc (61), nr (3), patata americana (1), pl (2), salame (3), salsiccia (11), wurstel (1) |
| Caviar | caviale (31) | catrame (1), ciliegie (1), lampone (1), mirtilli (6), mora (2), more (2), nc (22), nr (4), olive (1), perle (1), ribes (1), uva (2) |
| Cheese | formaggio (53) | caciotta (2), forma di formaggio (4), formaggio grana (1), nc (11), nr (3), pecorino (2), pl (2), ricotta (1), sapone (1), saponetta (3), tomino (1) |
| Chorizo | salsiccia (32) | chorizo (1), nc (16), nr (4), peperoncino (5), pigna (1), salame (7), salame piccante (4), salamino (3), salamino piccante (4), salsiccia di maiale (1), salsiccia piccante (2), sigaro (1), wurstel (1) |
| Cookie | biscotto (63) | biscotto ai cereali (1), biscotto frollino (1), frollino (2), nc (3), torta (5) |
| Creme caramel | creme caramel (38) | budino (21), budino al caramello (1), budino cr√®me caramel (1), cr√®me brul√® (1), dessert (1), nr (3), panna cotta (13), pl (4), pudding (1), souffl√® (1) |
| Fritter | No Response | bacon (1), biscotto (31), churro (2), churros (3), nc (15), nr (17), pancetta (1), pastafrolla (1), pl (7), sfogliatella (1), sfogliatina (2), tarallo (1) |
| Millefeuille | millefoglie (4) | cassata (1), diplomatico (1), dolce (16), fetta di torta (1), gelato (3), meringata (2), mignon (1), nc (18), nr (8), pasticcino (1), pl (8), porzione di torta (1), semifreddo (2), torta (15), torta alla ricotta (1), torta gelato (1) |
| Paella | paella (62) | gamberetti (1), misto di pesce (1), nc (2), nr (1), pasta (1), pasta alla marinara (1), pasta alle vongole (1), pasta allo scoglio (1), risotto (2), zuppa di pesce (2) |
| Pasty | panzerotto (23) | biscotto (1), biscotto della fortuna (1), brioche (1), calzone (3), cibo fritto (1), dolce (1), frittella (1), involtino primavera (1), nc (28), nr (7), panzerotto fritto (1), pizza fritta (1), pl (11), raviolo (2), saccottino (1), samosa (1) |
| Pie | torta (13) | biscotto (6), focaccia (10), frittata (1), nc (32), nr (6), pancake (1), pane (3), pasticciotto (1), pl (3), plum cake (1), rustico (1), salatino (1), torta di frutta (1) |
| Steak | bistecca (28) | arrosto (1), bistecca di vitello (1), braciola (8), carne (12), carne ai ferri (1), carne alla griglia (1), carne cotta (1), costata (1), fetta di carne (1), fettina di carne (1), manzo (1), minerale (1), nc (17) |
| **Furniture** |  |  |
| Armchair | poltrona (83) | poltrona in pelle (1) |
| Bed | letto (74) | letti (1), letto ad una piazza (2), letto antico (1), letto singolo (4) |
| Bedside table | comodino (55) | cassapanca (1), cassetti (1), cassettiera (2), cassetto (1), mobile (5), nr (3), pl (5), tavolino (1) |
| Bookcase | libreria (80) | armadio (2), pl (1), scaffale (1), vetrinetta (1) |
| Chair | sedia (79) | sedia antica (1), sedia di legno (1), seggiola (1) |
| Chest of drawers | cassettiera (29) | armadio (2), cassapanca (2), cassetti (1), cassettone (4), com√≤ (12), comodino (1), credenza (3), mobile (6), mobiletto (2), nr (3), pl (4), settimino (1) |
| Couch | divano (31) | chaise long (1), crinale (1), divanetto (2), divano antico (1), lettiga (1), lettino (3), lettino romano (1), nc (4), nr (8), ottomana (1), pl (15), poltrona (6), sofa (1), sof√† (2), triclino (6), triclivium (1) |
| Filling cabinet | schedario (6) | archivio (7), archivio a muro (1), armadietto (2), cassetti (2), cassettiera (25), cassettiera da ufficio (1), cassettiera portadocumenti (2), cassetto (13), cassetto dei documenti (1), mobile (1), mobiletto (2), mobiletto da ufficio (1), nc (1), nr (4), pl (6), portadocumenti (3), scaffale (2), settimanale (1), vano documenti (1) |
| Lamp | lampada (61) | abat jour (10), lampada da tavolo (2), lampadario (1), paralume (1) |
| Lectern | leggio (11) | anta (1), banco (1), cassetta di legno (1), cofanetto (1), mobile (3), nc (27), nr (18), panca (1), pl (9), poggialibro (1), porta (1), porta carte (1), portagioie (3), portagioielli (1), portalibro (1), reggilibro (1), scatola (1), scatoletta (1), scrigno (1) |
| Rocking chair | sedia a dondolo (79) | dondolo (1), pl (1), sedia (1) |
| Sofa | No Response | divano (74), divano di pelle (1) |
| Stool | sgabello (81) | sedia (2), seggiola (1), tavolo (1) |
| Table | tavolo (73) | tavola (3), tavolino (3), tavolino basso (1), tavolo in legno (2) |
| Wardrobe | armadio (71) | armadio da camera (1), guardaroba (1), mobile (1), porta abiti (1) |
| **Jewellery** |  |  |
| Bangle | braccialetto (34) | bracciale (26), braccialettto (1), catena (10), catenella (1), nc (5), nr (4), orologio (1), pl (2) |
| Bracelet | braccialetto (41) | bracciale (35), catena (1), catenina (3), collana (1), collier (1) |
| Brooch | spilla (54) | fermacapelli (1), fermaglio (1), gioiello (3), nc (7), nr (4), orecchino (3), spilla d'oro (1), spilla da balia (1) |
| Cufflinks | gemelli (45) | anelli (2), gioie (1), nc (11), nr (4), orecchini (13), paio di orecchini (1), pl (5), polsino (1), specchietti (1), spilla (1) |
| Diadem | diadema (22) | corona (49), coroncina (6), nr (1), pl (2), tiara (2) |
| Diamond | diamante (55) | brillante (1), cristallo (5), gemma (4), nc (1), nr (2), pietra (1), pietra preziosa (3), pl (3) |
| Medal | medaglia (6) | amuleto (1), cammeo (1), ciondolo (25), ciondolo con immagine santa (1), ciondolo religioso (1), icona (1), madonna (1), madonnina (1), medaglia con madonna (1), medaglietta (21), medaglietta religiosa (1), medaglione (5), monile (1), nc (2), nr (3), pallina (1), pendaglio (2), pendente (3), pl (7) |
| Necklace | collana (77) | braccialetto (2), collana blu (1), collana di turchesi (1), nr (1) |
| Pendant | orecchini (45) | fari (1), nc (3), nr (1), orecchini a perla (2), orecchini di perla (5), orecchini di perle (10), perle (7) |
| Ring | anello (85) |  |
| Seal ring | anello con sigillo (1) | anello (72), anello con stemma (2), anello d'oro (1), anello in oro (1), anello papale (1), anello per sigillo (1), pl (2), sigillo (1) |
| Tie clip | fermacravatta (7) | fermacapelli (2), fermacarte (1), fermaglio (3), fermo (1), ferrettino (1), fischietto (1), forcina (22), forcina per capelli (1), nc (30), nr (5), spilletta (1) |
| **Kitchen Utensils** |  |  |
| Cooking pot | No Response | anfora (1), bollitore (1), brocca (6), caraffa di terracotta (1), ciotola (1), contenitore (2), contenitore di terracotta (1), infusore (1), nc (18), nr (12), pentolame (1), pl (16), tazza (4), tazzina (1), teiera (10), uncorrect alternative response (1), vaso (8) |
| Cup | tazza (57) | bicchiere (14), pl (1), tazza di vetro (2), tazza verde (1), tazzina (6), tazzina in vetro (1) |
| Fondue | fonduta (11) | barbecue (1), bourguignon (2), fondue (1), fornellino (1), fornello (1), nc (24), nr (12), pentola (3), pentola per founduta (2), pentolino (1), pentolino per fonduta (1), pl (11), set da founduta (1), uncorrect alternative response (2) |
| Fork | forchetta (85) |  |
| Frying pan | padella (68) | padella antiaderente (3), pentola (10), pentola antiaderente (1) |
| Peeler | pelapatate (27) | coltello (1), lampadina (1), nc (25), nr (9), pelacarote (1), pelaverdura (1), pl (3), sbuccia carote (1), sbuccia patate (3), scalpello (1), togli torsolo (1), uncorrect alternative response (1) |
| Pot | pentola (76) | nr (1), pentola a pressione (3), pentola con coperchio (2) |
| Saucepan | casseruola (2) | pentola (67), pentola con coperchio (1), pentola terracotta (1), pl (1), tegame (3) |
| Sharpening steel | affilacoltelli (1) | acciarino (1), affila coltelli (8), affilalama (1), affilalame (1), affilalamine (1), affilatore (1), affilatrice (1), arrotacoltelli (1), cacciavite (22), cacciavite liscio (1), coltello (1), lima (3), limacoltelli (1), nc (9), nr (10), pl (8), punteruolo (1), scalpello (1), spada (7), spadino (2) |
| Small saucepan | pentolino (19) | casseruola (1), nr (1), padella (19), pentola (42), tegame (3) |
| Strainer | colino (18) | colabrodo (1), filtrino (1), mestolo (1), nc (2), nr (5), passatoio (1), passino (20), pl (9), retina (1), scolapasta (2), scolino (9), setaccio (5) |
| Teapot | teiera (71) | bollitore (4), caffettiera (1), nc (1), pl (8) |
| **Musical Instruments** |  |  |
| Accordion | fisarmonica (73) | armonica (4), pl (5) |
| Balalaika | balalaika (2) | cetra (1), chitarra (13), chitarra medievale (1), chitarrone (1), mandolino (1), nc (35), nr (10), pl (6), sitar (2), strumento musicale (1), ukulele (1) |
| Bugle | tromba (68) | corno (1), nc (4), pl (4), strumento musicale (1), trombetta (7) |
| Clarinet | clarinetto (43) | flauto (10), flauto traverso (4), nr (9), oboe (3), pl (9), saxofono (1), tromba (1), violoncello (1), xilofono (1) |
| Drum | tamburo (65) | batteria (2), grancassa (1), nr (1), percussioni (1), pl (2), rullante (3) |
| Flute | flauto (76) | flauto dolce (9) |
| Guitar | chitarra (62) | chitarra acustica (6), chitarra classica (14) |
| Harmonica | armonica (22) | armonica a bocca (8), diamonica (1), diapason (1), fisarmonica (26), nc (1), nr (1), pl (15) |
| Harp | arpa (79) | arco (1), arpa classica (1), cetra (1), pl (2), usignolo (1) |
| Maracas | maracas (65) | nacchere (3), nc (4), nr (6), pl (4) |
| Piano | pianoforte (57) | piano (3), piano a coda (1), pianoforte a coda (14) |
| Saxophone | sassofono (35) | contrabbasso (1), nc (1), nr (2), pl (3), sax (22), saxofono (9), tromba (9), trombone (3) |
| Tambourine | tamburello (43) | cembalo (11), clavicembalo (2), gioco (1), nr (4), piatto (1), pl (8), sonaglio (2), tamburello a sonagli (3), tamburello per bambini (1), tamburo (6) |
| Trumpet | tromba (72) | pl (1), trombetta (2) |
| Tuba | tuba (3) | corno (2), flicorno (1), nc (4), nr (9), pl (1), sassofono (1), strumento musicale (1), tromba (35), trombone (28) |
| Violin | violino (70) | chitarra classica (1), nr (1), pl (1), viola (2), violoncello (7) |
| **Sports/Games** |  |  |
| Ball | palla (67) | palla di gomma (1), pallone (15), pallone di gomma (1) |
| Chess | scacchi (29) | dama (2), gioco degli scacchi (1), scacchiera (46), scacchiera con pedine (2), scacchiera con scacchi (1), scacchiera in legno (1) |
| Dart | freccetta (62) | dardo (1), freccia (6), nc (1), pl (4), puntina (1) |
| Dartboard | bersaglio (23) | bersaglio per freccette (1), freccette (2), nr (8), pl (31), segnapunti (2), tabellone (2), tabellone da freccette (1), tabellone delle freccette (1), tabellone per freccette (1), tirassegno (1), tiro a segno (10), tiro al bersaglio (1) |
| Diabolo | No Response | attrezzo da giocoleria (1), diablo (14), gioco (1), nc (18), nr (36), pl (11), rola bola (1) |
| Dice | dado (74) | nr (1) |
| Doll | bambola (85) |  |
| Jump rope | corda per saltare (40) | corda (35), corda da ginnastica (1), corda per bambini (1), corda per fitness (1), fune (1), funicella (1), nr (1), pl (1) |
| Ludo | gioco da tavolo (11) | dama (1), gioco (1), gioco da tavola (14), gioco di societ√† (1), gioco in scatola (4), nc (23), non ti arrabbiare (2), nr (8), pl (1), trivial (3), trivial pursuit (5), twist (1) |
| Racket | racchetta (56) | pl (1), racchetta da tennis (28) |
| Skate | pattino (35) | monopattino (1), nr (1), pattino a rotelle (19), pattino in linea (4), pattino roller (1), roller (3), rollerblade (17) |
| Ski | sci (75) |  |
| Skittle | birillo (82) | birillo del bowling (1), nr (1), pl (1) |
| Soccer ball | pallone da calcio (24) | palla (2), palla da calcio (1), pallone (47), pallone di cuoio (1) |
| Spinning top | trottola (58) | bobina (1), fuso (1), girandola (2), lampadina (1), nc (7), nr (10), pl (2) |
| Table football | calcio balilla (17) | biliardino (23), biliardo (1), calcetto (20), calcetto balila (1), calcetto balilla (6), nr (1), pl (3), tavolo da biliardo (1), uncorrect alternative response (1) |
| **Tools** |  |  |
| Axe | ascia (33) | accetta (33), falce (1), mannaia (1), martello (9), nr (1), pl (6), scure (1) |
| Bit | punta da trapano (7) | cacciavite (2), nc (12), nr (19), pl (5), punta (2), punta del trapano (11), punta di trapano (4), punta per trapano (2), punta perforatrice (1), trapano (1), vite (15), vite da trapano (1) |
| Chisel | No Response | cacciavite (2), lima (5), lima da tavolo (1), nc (21), nr (17), pl (7), scalpello (16), spatola (5), uncorrect alternative response (1) |
| Cold chisel | scalpello (15) | asta di ferro (1), barra di ferro (1), bastone di ferro (1), chiodo (8), lama (1), leva (1), nc (34), nr (7), picchetto (1), piede di porco (1), pl (10), scalpellino (1), spranga (1), spranga di ferro (1), utensile (1) |
| Hammer | martello (80) | nr (2) |
| Handsaw | sega (68) | nc (1), sega a mano (2), seghetto (3) |
| Leveller | livella (9) | bolla (16), calibro (1), caricabatterie (1), livella a bolla (1), misurapendenza (1), n (1), nc (30), nr (9), pl (13), righello (1), strumento tecnico per misurare (1) |
| Nail (tool) | chiodo (74) | asta con punta (1), chiodo in ferro (1), nr (1), pl (2), vite (3) |
| Nut | dado (7) | bobina (1), bullone (52), nc (1), nr (5), pl (2), rondella (1), vite (6) |
| Pincers | pinze (7) | attrezzo (1), nc (1), nr (2), pinza (47), pl (18), tenaglia (7), tronchesi (1) |
| Pliers | tenaglie (5) | cacciavite (2), cagnetta (1), cesoia (1), chiave (1), chiave inglese (3), nc (2), nr (5), pinza (23), pinze (5), pl (12), tenaglia (20), trancino (1) |
| Screw | vite (46) | bullone (17), chiodo (2), dado (1), nr (3), pl (6) |
| Screwdriver | cacciavite (75) | cacciavite a linea (1), cacciavite a taglio (2), cacciavite italiano (1), giravite (2), pl (4) |
| Shovel | pala (40) | badile (20), pala da lavoro (1), paletta (3), pl (2), vanga (6), zappa (9) |
| Trowel | cazzuola (5) | attrezzo edile (1), levigatrice (1), manico (1), maniglia (2), nc (13), nr (19), pialla (7), piallatrice (1), pl (9), spatola (16) |
| **Vehicles** |  |  |
| Boat | barca (68) | barca a remi (6), barchetta (3), barchetta giocattolo (1), nr (1), peschereccio (1), pl (1), scialuppa (3), uncorrect alternative response (1) |
| Bus | autobus (22) | bus (5), bus turistico (1), corriera (16), pullman (36), pullman granturismo (1) |
| Car | automobile (37) | auto (7), auto peugeot (2), automobile peugeot 206 (1), automobile utilitaria (1), autovettura (1), berlina (1), macchina (14), macchina peugeot (2), peugeot (3), peugeot 206 (5), peugeot 208 (1) |
| Cart | carro (28) | carretto (37), carriola (8), carro da traino (1), carrucola (1), nc (1), nr (2), pl (4), traino (2) |
| Motorbike | moto (54) | moto 125 (1), moto da corsa (2), moto turismo (1), motocicletta (19), motociclettta (1), motociclo (2), motore (1), motorino (1) |
| Paragliding | parapendio (8) | deltaplano (9), nr (1), paracadute (33), paracadutismo (2), paracadutista (7), pl (14), tandem (1) |
| Plane | aereo (66) | aereo di linea (2), aeroplano (13), boeing (2), boeing 747 (2) |
| Scooter | monopattino (80) | pl (2) |
| Ship | nave (2) | barca (45), barca a motore (1), barca da pesca (2), barca merci (1), barca per pescatori (1), motoscafo (3), nc (1), nr (1), peschereccio (8), pl (6), traghetto (2) |
| Skateboard | skateboard (79) | skate (6) |
| Tractor | trattore (82) |  |
| Train | treno (71) | metro (1), metropolitana (2), nr (1) |
| Van | furgone (63) | camion (3), camioncino (6), furgoncino (9), pl (1), pulmino (2), van (1) |
| **Weapons** |  |  |
| Armour | armatura (72) | armatura completa (1), armatura da cavaliere (1), armatura medievale (1), armatura medioevale (2), corazza (1), nr (1), pl (2) |
| Arrow | freccia (72) | dardo (1), lancia (1), pl (1) |
| Bayonet | baionetta (10) | freno (1), nc (62), nr (8), paletta (1), pl (2), rubinetto (1) |
| Boomerang | boomerang (54) | anguilla (1), arco (1), frisbee (4), giavellotto (1), nc (9), nr (3), oloturia (1), pl (5), tegolina (2), volano (1) |
| Bow | arco (74) | arco di legno (1) |
| Cannon | cannone (80) | carro armato (1), nc (1), pl (2) |
| Crossbow | balestra (31) | arco (6), baionetta (1), balaustra (1), catapulta (1), nc (21), nr (10), pl (6), sega (5) |
| Grenade | granata (16) | bomba (11), bomba a mano (30), borraccia (1), lanterna (1), mina (2), nc (11), nr (2), pl (1) |
| Gun | pistola (74) | doppietta (1), luger (2), nc (2), nr (1), pistola semiautomatica (1), rivoltella (4) |
| Helmet | elmo (22) | cappello (4), caschetto (3), casco (3), elmetto (33), elmetto da guerra (2), elmetto in ferro (1), elmetto militare (1), elmo da guerra (1), elmo da lavoratore (1), nc (3), nr (6), pl (1) |
| Machine gun | mitragliatrice (10) | arma (2), carabina (3), fucile (33), fucile da cecchino (1), fucile mitragliatore (1), mitra (4), mitraglia (1), mitragliatore (2), nc (13), nr (4), pl (1) |
| Revolver | No Response | nr (1), pistola (78), revolver (6) |
| Shield | scudo (72) | pl (1), scudetto (1), scudo medievale (1), stemma (5), stemma araldico (1), stendardo (1) |
| Slingshot | fionda (63) | fune (1), lancia (1), nc (2), nr (1), pl (7) |
| Sword | spada (81) | nr (2), spadaccino (1), spadino (1) |

Proportion of target names in brackets; Alternative names, acceptanle synonyms of each item, plus “Don’t Know - non conosco” (nc), “Don’t remember – non ricordo” (nr), “Tip of the tongue – punta della lingua” (pl) responses
