# Supplementary material for: Italian norms and naming latencies for 357 high quality color images
Source: PLoS One. 2019 Feb 22;14(2):e0209524. doi: 10.1371/journal.pone.0209524 (PMC6386297; doi:10.1371/journal.pone.0209524)
Supplement: S3 Table — (DOCX) [file pone.0209524.s003.docx]

| English | Italian | Difficulty | Point Biserial | Biserial |
| --- | --- | --- | --- | --- |
| **Animals** |  |  |  |  |
| Armadillo | armadillo | 0.31 | 0.47 | 0.61 |
| Bat | pipistrello | 1 | NA | NA |
| Cat | gatto | 0.95 | -0.19 | -0.39 |
| Cheetah | ghepardo | 0.4 | 0.26 | 0.33 |
| Cow | mucca | 0.83 | 0.29 | 0.42 |
| Crocodile | coccodrillo | 1 | NA | NA |
| Dromedary | dromedario | 0.69 | 0.26 | 0.33 |
| Elephant | elefante | 0.94 | 0.04 | 0.07 |
| Giraffe | giraffa | 1 | NA | NA |
| Hippopotamus | ippopotamo | 0.84 | 0.28 | 0.41 |
| Horse | cavallo | 1 | NA | NA |
| Kangaroo | canguro | 1 | NA | NA |
| Lioness | leonessa | 0.6 | 0.18 | 0.23 |
| Lynx | lince | 0.5 | -0.27 | -0.33 |
| Platypus | ornitorinco | 0.21 | 0.51 | 0.7 |
| Rhino | rinoceronte | 0.96 | 0.07 | 0.15 |
| Snake | serpente | 0.78 | 0.25 | 0.34 |
| Tapir | tapiro | 0.32 | 0.46 | 0.59 |
| Tiger | tigre | 0.96 | 0.08 | 0.19 |
| Turtle | tartaruga | 0.83 | 0.42 | 0.6 |
| Zebra | zebra | 1 | NA | NA |
| **Birds** |  |  |  |  |
| Barn owl | barbagianni | 0.13 | 0.08 | 0.13 |
| Duck | anatra | 0.44 | 0.27 | 0.32 |
| Goldfinch | cardellino | 0 | NA | NA |
| Goose | oca | 0.84 | 0.26 | 0.39 |
| Hen | gallina | 0.83 | 0.38 | 0.56 |
| Hummingbird | colibrì | 0.26 | 0.56 | 0.73 |
| Kiwi | kiwi | 0.15 | 0.36 | 0.55 |
| Magpie | gazza | 0.33 | -0.33 | -0.42 |
| Ostrich | struzzo | 0.95 | 0.11 | 0.22 |
| Owl | gufo | 0.87 | 0.08 | 0.12 |
| Partridge | pernice | 0.11 | -0.05 | -0.09 |
| Pelican | pellicano | 0.42 | 0.42 | 0.52 |
| Penguin | pinguino | 0.98 | 0.05 | 0.14 |
| Pheasant | fagiano | 0.56 | 0.26 | 0.31 |
| Pigeon | piccione | 0.89 | 0.47 | 0.78 |
| Raven | corvo | 0.78 | 0.3 | 0.42 |
| Rooster | gallo | 0.78 | 0.22 | 0.3 |
| Seagull | gabbiano | 0.89 | 0.43 | 0.71 |
| Sparrow | passero | 0.38 | 0.17 | 0.22 |
| Toucan | tucano | 0.39 | 0.6 | 0.74 |
| **Body Parts** |  |  |  |  |
| Arm | braccio | 0.79 | 0.39 | 0.53 |
| Beard | barba | 0.2 | 0.32 | 0.45 |
| Bone | osso | 0.83 | 0.6 | 0.88 |
| Brain | cervello | 1 | NA | NA |
| Ear | orecchio | 0.98 | 0.19 | 0.57 |
| Eye | occhio | 1 | NA | NA |
| Finger | dito | 0.26 | 0.1 | 0.13 |
| Foot | piede | 1 | NA | NA |
| Hand | mano | 0.67 | 0.36 | 0.45 |
| Kidney | rene | 0.11 | 0.1 | 0.17 |
| Leg | gamba | 0.95 | -0.02 | -0.03 |
| Liver | fegato | 0.5 | 0.25 | 0.3 |
| Lung | polmone | 0.21 | 0.09 | 0.13 |
| Mouth | bocca | 0.51 | 0.36 | 0.45 |
| Nail (bodypart) | unghia | 0.94 | 0.04 | 0.07 |
| Nose | naso | 1 | NA | NA |
| Pelvis | bacino | 0.53 | 0.52 | 0.63 |
| Skull | cranio | 0.16 | -0.07 | -0.1 |
| Tongue | lingua | 0.89 | 0.47 | 0.78 |
| Vertebra | vertebra | 0.07 | 0.21 | 0.38 |
| **Flowers** |  |  |  |  |
| Araceae | calla | 0.39 | 0.31 | 0.39 |
| Bellflower | campanule | 0.11 | 0.06 | 0.1 |
| Carnation | garofano | 0.2 | 0.13 | 0.19 |
| Daisy | margherita | 0.94 | -0.22 | -0.44 |
| Geranium | geranio | 0.11 | 0.3 | 0.49 |
| Lilac | lilla | 0.07 | 0.18 | 0.34 |
| Orchid | orchidea | 0.33 | 0.02 | 0.02 |
| Pansy | viola del pensiero | 0 | NA | NA |
| Poppy | papavero | 0.85 | 0.28 | 0.43 |
| Rose | rosa | 0.94 | 0.24 | 0.48 |
| Sunflower | girasole | 1 | NA | NA |
| Tulip | tulipano | 0.78 | 0.15 | 0.21 |
| **Fruits** |  |  |  |  |
| Apple | mela | 0.94 | 0.19 | 0.38 |
| Avocado | avocado | 0.16 | 0.15 | 0.23 |
| Banana | banana | 1 | NA | NA |
| Cherries | ciliegie | 0.94 | 0.45 | 0.89 |
| Coconut | cocco | 0.53 | 0.21 | 0.26 |
| Custard apple | anona | 0 | NA | NA |
| Fig | fico | 0.61 | 0.51 | 0.63 |
| Flat peach | pesca tabacchiera | 0 | NA | NA |
| Grapes | uva | 0.78 | 0.25 | 0.34 |
| Kiwi fruit | kiwi | 0.94 | 0.19 | 0.38 |
| Lemon | limone | 1 | NA | NA |
| Mango | mango | 0.15 | -0.06 | -0.09 |
| Melon | melone | 0.11 | 0.32 | 0.52 |
| Orange | arancia | 1 | NA | NA |
| Peach | pesca | 0.29 | 0.03 | 0.04 |
| Pear | pera | 1 | NA | NA |
| Pomegranate | melagrana | 0.05 | 0.11 | 0.22 |
| Quince | mela cotogna | 0.02 | 0.14 | 0.42 |
| Redcurrant | ribes | 0.44 | 0.43 | 0.53 |
| Strawberry | fragola | 1 | NA | NA |
| Watermelon | anguria | 0.89 | -0.13 | -0.22 |
| **Insects** |  |  |  |  |
| Ant | formica | 0.94 | -0.01 | -0.03 |
| Bee | ape | 0.32 | 0.4 | 0.51 |
| Beetle | scarabeo | 0.47 | 0.23 | 0.28 |
| Butterfly | farfalla | 1 | NA | NA |
| Centipede | millepiedi | 0.47 | 0.38 | 0.47 |
| Cockroach | scarafaggio | 0.51 | 0.04 | 0.04 |
| Dragonfly | libellula | 0.78 | 0.05 | 0.07 |
| Fly | mosca | 0.95 | 0.68 | 1 |
| Grasshopper | cavalletta | 0.75 | 0.35 | 0.47 |
| Ladybird | coccinella | 0.83 | 0.32 | 0.47 |
| Mosquito | zanzara | 0.63 | 0.27 | 0.34 |
| Moth | tarma | 0 | NA | NA |
| Praying mantis | mantide religiosa | 0.33 | -0.56 | -0.7 |
| Scorpion | scorpione | 0.84 | 0.58 | 0.86 |
| Spider | ragno | 0.69 | -0.14 | -0.18 |
| Termite | termite | 0.11 | -0.05 | -0.09 |
| Wasp | vespa | 0.21 | 0.33 | 0.46 |
| **Marine Creatures** |  |  |  |  |
| Cockle | conchiglia | 0.91 | 0.06 | 0.1 |
| Crab | granchio | 1 | NA | NA |
| Dolphin | delfino | 1 | NA | NA |
| Eel | anguilla | 0.36 | 0.2 | 0.26 |
| Goose barnacle | cirripedi | 0 | NA | NA |
| Killer whale | orca | 0.68 | 0.51 | 0.65 |
| Lobster | aragosta | 0.53 | 0.53 | 0.65 |
| Manatee | lamantino | 0.06 | 0.01 | 0.03 |
| Mussel | cozza | 0.89 | 0.2 | 0.32 |
| Narwhal | narvalo | 0.05 | 0.3 | 0.61 |
| Oyster | ostrica | 0.17 | 0.15 | 0.22 |
| Pomfret | pampo | 0 | NA | NA |
| Ray | razza | 0.31 | 0.26 | 0.33 |
| Razor-shell | cannolicchio | 0 | NA | NA |
| Shark | squalo | 0.42 | 0.36 | 0.45 |
| Sperm whale | capodoglio | 0.07 | 0.06 | 0.1 |
| Starfish | stella marina | 1 | NA | NA |
| Whale | balena | 0.37 | 0.21 | 0.26 |
| **Nuts** |  |  |  |  |
| Acorn | ghianda | 0.64 | 0.11 | 0.14 |
| Almond | mandorla | 0.39 | -0.17 | -0.21 |
| Chestnut | castagna | 0.63 | 0.27 | 0.34 |
| Date | dattero | 0.29 | 0.19 | 0.26 |
| Hazelnut | nocciola | 0.39 | 0.55 | 0.68 |
| Peanut | arachide | 0.47 | 0.56 | 0.68 |
| Pine kernel | pinolo | 0.02 | -0.05 | -0.14 |
| Pipe | seme | 0.22 | -0.36 | -0.49 |
| Pistachio | pistacchio | 0.63 | 0.56 | 0.7 |
| Raisin | uva passa | 0.07 | 0.13 | 0.24 |
| Walnut | noce | 0.89 | 0.05 | 0.09 |
| **Trees** |  |  |  |  |
| Black poplar | pioppo | 0 | NA | NA |
| Cedar | cedro | 0 | NA | NA |
| Cypress | cipresso | 0.39 | 0.05 | 0.06 |
| Eucalyptus | eucalipto | 0 | NA | NA |
| Fig tree | fico | 0.04 | -0.11 | -0.26 |
| Fir | abete | 0.44 | 0.38 | 0.47 |
| Holm oak | leccio | 0 | NA | NA |
| Olive tree | olivo | 0.04 | 0.22 | 0.51 |
| Palm tree | palma | 0.94 | -0.01 | -0.03 |
| Pine tree | pino | 0.11 | 0.2 | 0.33 |
| Willow | salice | 0.36 | 0.47 | 0.6 |
| **Vegetables** |  |  |  |  |
| Artichoke | carciofo | 0.78 | 0.36 | 0.49 |
| Asparagus | asparago | 0.74 | 0.65 | 0.85 |
| Cabbage | cavolo | 0.09 | -0.08 | -0.13 |
| Carrot | carota | 0.94 | 0.19 | 0.38 |
| Cauliflower | cavolfiore | 0.68 | 0.47 | 0.6 |
| Celery | sedano | 0.85 | -0.08 | -0.13 |
| Chard | bietola | 0.22 | -0.36 | -0.49 |
| Cucumber | cetriolo | 0.74 | 0.51 | 0.67 |
| Eggplant | melanzana | 0.98 | 0.23 | 0.69 |
| Endive | indivia | 0.22 | -0.36 | -0.49 |
| Leek | porro | 0.42 | 0.44 | 0.54 |
| Lettuce | lattuga | 0.27 | 0.09 | 0.12 |
| Onion | cipolla | 0.94 | 0.24 | 0.48 |
| Pepper | peperone | 0.84 | -0.29 | -0.42 |
| Potato | patata | 0.98 | 0 | 0.01 |
| Pumpkin | zucca | 0.89 | -0.4 | -0.64 |
| Red cabbage | cavolo rosso | 0 | NA | NA |
| Spinach | spinaci | 0.35 | 0.27 | 0.35 |
| Tomato | pomodoro | 0.78 | 0.36 | 0.49 |
| Turnip | rapa | 0 | NA | NA |
| **Nature** |  |  |  |  |
| Cliff | scogliera | 0.42 | 0.25 | 0.31 |
| Cloud | nuvola | 0.95 | 0.23 | 0.46 |
| Coal | carbone | 0.28 | 0.14 | 0.18 |
| Gold | oro | 0.05 | -0.19 | -0.38 |
| Ice | ghiaccio | 0.22 | 0.15 | 0.21 |
| Iceberg | iceberg | 0.83 | -0.22 | -0.31 |
| Island | isola | 0.95 | 0.68 | 1 |
| Moon | luna | 0.84 | 0.13 | 0.19 |
| Mountain | montagna | 0.89 | -0.02 | -0.03 |
| Puddle | pozzanghera | 0.53 | 0.02 | 0.02 |
| Sea | mare | 0.96 | -0.16 | -0.37 |
| Stone | pietra | 0.22 | 0.14 | 0.2 |
| Sun | sole | 1 | NA | NA |
| Volcano | vulcano | 0.98 | 0.19 | 0.57 |
| Waterfall | cascata | 0.72 | 0.25 | 0.33 |
| Wave | onda | 0.42 | 0.18 | 0.22 |
| **Buildings** |  |  |  |  |
| Castle | castello | 0.69 | 0.2 | 0.26 |
| Cathedral | cattedrale | 0.44 | -0.16 | -0.2 |
| Church | chiesa | 0.68 | 0.18 | 0.23 |
| Factory | fabbrica | 0.49 | 0.32 | 0.4 |
| Granary | granaio | 0 | NA | NA |
| House | casa | 0.84 | -0.04 | -0.05 |
| Lighthouse | faro | 0.76 | 0.19 | 0.26 |
| Mill | mulino | 0.56 | 0.4 | 0.49 |
| Pagoda | pagoda | 0.05 | 0.19 | 0.39 |
| Palace | palazzo | 0.15 | 0.24 | 0.37 |
| Pyramid | piramide | 0.89 | -0.32 | -0.52 |
| Shanty | baracca | 0.32 | 0.37 | 0.47 |
| Silo | silo | 0.02 | 0.08 | 0.23 |
| Skyscraper | grattacielo | 0.94 | 0.14 | 0.28 |
| Tower | torre | 0.79 | 0.48 | 0.65 |
| **Clothing** |  |  |  |  |
| Bathrobe | accappatoio | 1 | NA | NA |
| Biretta | tocco | 0 | NA | NA |
| Cap | cappello | 0.32 | -0.47 | -0.6 |
| Clog | zoccolo | 0.42 | 0.41 | 0.52 |
| Coat | cappotto | 0.83 | 0.23 | 0.33 |
| Glove | guanto | 0.79 | 0.15 | 0.2 |
| Jacket | giacca | 0.89 | 0.24 | 0.4 |
| Shirt | camicia | 1 | NA | NA |
| Shoe | scarpa | 0.84 | -0.15 | -0.23 |
| Skirt | gonna | 0.96 | 0.29 | 0.67 |
| Socks | calzini | 0.56 | 0.3 | 0.37 |
| Trousers | pantaloni | 1 | NA | NA |
| Undershirt | canottiera | 0.76 | 0.1 | 0.14 |
| **Desk Material** |  |  |  |  |
| Compasses | compasso | 1 | NA | NA |
| Eraser | gomma | 0.47 | 0.04 | 0.05 |
| Felt-tip pen | pennarello | 0.64 | 0.25 | 0.32 |
| Folder | cartella | 0.11 | -0.09 | -0.15 |
| Fountain pen | penna stilografica | 0.47 | 0.03 | 0.04 |
| Ink pad | tampone di inchiostro | 0 | NA | NA |
| Paperclip | graffetta | 0.83 | -0.37 | -0.54 |
| Pen | penna | 0.58 | -0.09 | -0.11 |
| Pencil | matita | 0.96 | 0.04 | 0.08 |
| Pencil sharpener | temperino | 0.83 | -0.03 | -0.04 |
| Rubber stamp | timbro | 0.68 | 0.38 | 0.48 |
| Ruler | righello | 0.95 | 0.27 | 0.55 |
| Set square | squadra | 0.91 | 0.07 | 0.12 |
| Square ruler | squadra | 0.94 | -0.07 | -0.13 |
| Stapler | pinzatrice | 0 | NA | NA |
| **Food** |  |  |  |  |
| Anchovy | acciughe | 0.28 | -0.04 | -0.05 |
| Black pudding | sanguinaccio | 0 | NA | NA |
| Caviar | caviale | 0.38 | 0.54 | 0.69 |
| Cheese | formaggio | 0.67 | 0.53 | 0.67 |
| Chorizo | salsiccia | 0.26 | 0.16 | 0.2 |
| Cookie | biscotto | 0.85 | 0.14 | 0.21 |
| Creme caramel | creme caramel | 0.44 | 0.31 | 0.38 |
| Fritter | frittella | 0 | NA | NA |
| Millefeuille | millefoglie | 0.11 | 0.47 | 0.76 |
| Paella | paella | 0.84 | 0.16 | 0.23 |
| Pasty | panzerotto | 0.22 | 0.06 | 0.08 |
| Pie | torta | 0.21 | -0.09 | -0.12 |
| Steak | bistecca | 0.35 | 0.08 | 0.11 |
| **Furniture** |  |  |  |  |
| Armchair | poltrona | 0.94 | -0.12 | -0.23 |
| Bed | letto | 0.89 | -0.2 | -0.33 |
| Bedside table | comodino | 0.73 | 0.02 | 0.03 |
| Bookcase | libreria | 0.72 | 0.33 | 0.43 |
| Chair | sedia | 0.95 | 0.03 | 0.05 |
| Chest of drawers | cassettiera | 0.42 | 0.1 | 0.12 |
| Couch | divano | 0.28 | 0.01 | 0.01 |
| Filling cabinet | schedario | 0.05 | 0 | 0 |
| Lamp | lampada | 0.84 | 0.22 | 0.32 |
| Lectern | leggio | 0.28 | 0.06 | 0.08 |
| Rocking chair | sedia a dondolo | 0.84 | 0.28 | 0.41 |
| Sofa | sofà | 0 | NA | NA |
| Stool | sgabello | 1 | NA | NA |
| Table | tavolo | 0.84 | 0.06 | 0.09 |
| Wardrobe | armadio | 0.95 | 0.04 | 0.09 |
| **Jewellery** |  |  |  |  |
| Bangle | braccialetto | 0.56 | -0.12 | -0.15 |
| Bracelet | braccialetto | 0.37 | -0.1 | -0.12 |
| Brooch | spilla | 0.69 | 0.01 | 0.02 |
| Cufflinks | gemelli | 0.5 | 0.18 | 0.21 |
| Diadem | diadema | 0.26 | 0.23 | 0.3 |
| Diamond | diamante | 0.76 | 0.36 | 0.49 |
| Medal | medaglia | 0.06 | 0.01 | 0.03 |
| Necklace | collana | 1 | NA | NA |
| Pendant | orecchini | 0.64 | 0.27 | 0.35 |
| Ring | anello | 0.78 | 0.36 | 0.49 |
| Seal ring | anello con sigillo | 0 | NA | NA |
| Tie clip | fermacravatta | 0.11 | -0.11 | -0.18 |
| **Kitchen Utensils** |  |  |  |  |
| Cooking pot | pentola da cottura | 0.22 | -0.36 | -0.49 |
| Cup | tazza | 0.84 | 0.16 | 0.24 |
| Fondue | fonduta | 0.13 | 0.01 | 0.02 |
| Fork | forchetta | 0.89 | 0.24 | 0.39 |
| Frying pan | padella | 0.68 | 0.59 | 0.75 |
| Peeler | pelapatate | 0.42 | 0.08 | 0.1 |
| Pot | pentola | 0.95 | 0.03 | 0.05 |
| Saucepan | casseruola | 0 | NA | NA |
| Sharpening steel | affilacoltelli | 0 | NA | NA |
| Small saucepan | pentolino | 0.28 | -0.33 | -0.43 |
| Strainer | colino | 0.25 | -0.02 | -0.02 |
| Teapot | teiera | 0.61 | 0.19 | 0.24 |
| **Musical Instruments** |  |  |  |  |
| Accordion | fisarmonica | 0.84 | 0.53 | 0.78 |
| Balalaika | balalaika | 0.02 | 0.16 | 0.48 |
| Bugle | tromba | 0.78 | 0.08 | 0.11 |
| Clarinet | clarinetto | 0.42 | 0.23 | 0.28 |
| Drum | tamburo | 0.87 | 0.2 | 0.31 |
| Flute | flauto | 0.94 | 0.19 | 0.38 |
| Guitar | chitarra | 0.68 | 0.31 | 0.4 |
| Harmonica | armonica | 0.33 | 0.38 | 0.48 |
| Harp | arpa | 0.94 | -0.12 | -0.23 |
| Maracas | maracas | 0.68 | 0.35 | 0.45 |
| Piano | pianoforte | 0.76 | 0.25 | 0.34 |
| Saxophone | sassofono | 0.72 | 0.23 | 0.29 |
| Tambourine | tamburello | 0.37 | 0.17 | 0.21 |
| Trumpet | tromba | 0.95 | 0.15 | 0.31 |
| Tuba | tuba | 0.28 | -0.09 | -0.12 |
| Violin | violino | 0.89 | 0.36 | 0.58 |
| **Sports/Games** |  |  |  |  |
| Ball | palla | 0.89 | 0.28 | 0.45 |
| Chess | scacchi | 0.21 | -0.4 | -0.55 |
| Dart | freccetta | 0.84 | 0.15 | 0.22 |
| Dartboard | bersaglio | 0.33 | -0.56 | -0.7 |
| Diabolo | diabolo | 0 | NA | NA |
| Dice | dado | 0.98 | 0 | 0.01 |
| Doll | bambola | 1 | NA | NA |
| Jump rope | corda per saltare | 0.42 | 0.24 | 0.29 |
| Ludo | gioco da tavolo | 0.16 | 0.07 | 0.1 |
| Racket | racchetta | 0.67 | 0.46 | 0.58 |
| Skate | pattino | 0.37 | -0.56 | -0.7 |
| Ski | sci | 1 | NA | NA |
| Skittle | birillo | 0.83 | 0.29 | 0.42 |
| Soccer ball | pallone da calcio | 0.29 | 0.02 | 0.03 |
| Spinning top | trottola | 0.58 | 0.66 | 0.81 |
| Table football | calcio balilla | 0.24 | 0.15 | 0.2 |
| **Tools** |  |  |  |  |
| Axe | ascia | 0.44 | 0.5 | 0.61 |
| Bit | punta da trapano | 0 | NA | NA |
| Chisel | cesello | 0 | NA | NA |
| Cold chisel | scalpello | 0.28 | -0.51 | -0.67 |
| Hammer | martello | 1 | NA | NA |
| Handsaw | sega | 0.91 | 0.33 | 0.57 |
| Leveller | livella | 0.06 | -0.24 | -0.48 |
| Nail (tool) | chiodo | 0.95 | 0.68 | 1 |
| Nut | dado | 0.09 | 0.18 | 0.32 |
| Pincers | pinze | 0 | NA | NA |
| Pliers | tenaglie | 0.16 | 0.55 | 0.82 |
| Screw | vite | 0.62 | 0.13 | 0.17 |
| Screwdriver | cacciavite | 0.83 | 0.54 | 0.79 |
| Shovel | pala | 0.47 | 0.29 | 0.35 |
| Trowel | cazzuola | 0.04 | 0.11 | 0.26 |
| **Vehicles** |  |  |  |  |
| Boat | barca | 0.78 | 0.42 | 0.57 |
| Bus | autobus | 0.16 | 0.14 | 0.2 |
| Car | automobile | 0.47 | 0 | 0.01 |
| Cart | carro | 0.28 | 0.43 | 0.56 |
| Motorbike | moto | 0.63 | -0.12 | -0.15 |
| Paragliding | parapendio | 0.11 | 0.33 | 0.54 |
| Plane | aereo | 0.78 | 0.45 | 0.61 |
| Scooter | monopattino | 0.95 | 0.03 | 0.05 |
| Ship | nave | 0.04 | -0.02 | -0.05 |
| Skateboard | skateboard | 0.78 | 0.48 | 0.65 |
| Tractor | trattore | 1 | NA | NA |
| Train | treno | 0.95 | 0.03 | 0.06 |
| Van | furgone | 0.72 | 0.51 | 0.67 |
| **Weapons** |  |  |  |  |
| Armour | armatura | 0.89 | 0.22 | 0.35 |
| Arrow | freccia | 0.96 | 0.15 | 0.36 |
| Bayonet | baionetta | 0.22 | -0.28 | -0.38 |
| Boomerang | boomerang | 0.68 | 0.48 | 0.61 |
| Bow | arco | 0.98 | 0.15 | 0.44 |
| Cannon | cannone | 1 | NA | NA |
| Crossbow | balestra | 0.37 | 0.45 | 0.56 |
| Grenade | granata | 0.22 | 0.46 | 0.64 |
| Gun | pistola | 0.89 | -0.1 | -0.15 |
| Helmet | elmo | 0.26 | 0.5 | 0.66 |
| Machine gun | mitragliatrice | 0.15 | 0.16 | 0.25 |
| Revolver | rivoltella | 0 | NA | NA |
| Shield | scudo | 0.95 | 0.03 | 0.05 |
| Slingshot | fionda | 0.85 | 0.42 | 0.65 |
| Sword | spada | 1 | NA | NA |

**Note. Indexes of individual item analysis including a measure of item difficulty and two indexes of item discrimination based on item-test correlations (point-biserial and biserial).** NA = not available
