# Supplementary material for: Italian norms and naming latencies for 357 high quality color images
Source: PLoS One. 2019 Feb 22;14(2):e0209524. doi: 10.1371/journal.pone.0209524 (PMC6386297; doi:10.1371/journal.pone.0209524)
Supplement: S1 Text — (DOCX) [file pone.0209524.s004.docx]

**Supplemental Analysis**

*Analysis Type-2* including all the variables. A multiple regression analysis was conducted with the following variables: Agreement, H statistic, typicality, AoA, familiarity, frequency, manipulability and visual complexity. As can be seen in Table S1, significant effects of agreement, H statistic, typicality, AoA and manipulability were obtained; while the effect of familiarity, frequency and visual complexity were not significant. However, some Tolerance statistics were below .5 and the average of the variance inflation factor (VIF) was 4.674, suggesting collinearity problems on the regression model [1].

**S1 Table. Results of the multiple regression analysis, with naming latencies as the criterion variable and agreement, H statistic, typicality, age of acquisition, familiarity, lexical frequency, manipulability and visual complexity as predictors variables.**

|  | Estimate | St.Error | t value | Pr (>\|t\|) | Tolerance | VIF |
| --- | --- | --- | --- | --- | --- | --- |
| Intercept | 248.856 | 290.014 | 0.858 | 0.391 |  | - |
| Agreement | 528.701 | 260.683 | 2.028 | 0.0439 | 0.079 | 12.511 |
| H statistic | 274.271 | 59.786 | 4.588 | 8.20x10^-6^ | 0.075 | 13.310 |
| Typicality | -51.634 | 18.248 | -2.830 | 0.005 | 0.565 | 1.767 |
| Age of acquisition | 110.713 | 17.557 | 6.306 | 2.01x10^-9^ | 0.360 | 2.773 |
| Familiarity | 19.360 | 19.744 | 0.981 | 0.328 | 0.352 | 2.838 |
| Lexical frequency | -1.076 | 7.353 | -0.146 | 0.883 | 0.685 | 1.457 |
| Manipulability | -20.781 | 8.850 | -2.348 | 0.019 | 0.790 | 1.264 |
| Visual complexity | -11.250 | 17.573 | -0.640 | 0.522 | 0.679 | 1.472 |

Notes: VIF = variance inflation factor. Degrees of freedom = 187.

__________________________________________________________________________________

*Analysis Type-3* including all the variables. Naming latencies were analyzed using mixed effects regression model performed at the single trial level with all the variables as predictors (see main text for details). As can be seen in Table S2, significant effects of agreement, H statistic, typicality and AoA were obtained; the effect of manipulability was marginally significant; while the effects of familiarity, frequency and visual complexity were not significant.

**S2 Table. Results of Mixed effects Model with naming latencies as the criterion variable and agreement, H statistic, typicality, age of acquisition, familiarity, lexical frequency, manipulability and visual complexity as predictors variables.**

|  | Estimate | St.Error | t value | Pr (>\|t\|) |
| --- | --- | --- | --- | --- |
| Intercept | 1.572 | 1.446x10^-3^ | 1087.063 | < .001 |
| Agreement | 1.389x10^-3^ | 6.940x10^-4^ | 2.001 | 0.046 |
| H statistic | 1.724x10^-3^ | 2.203x10^-4^ | 7.824 | < .001 |
| Typicality | -7.030x10^-4^ | 1.730x10^-4^ | -4.064 | < .001 |
| Age of acquisition | 9.798x10^-4^ | 1.550x10^-4^ | 6.320 | < .001 |
| Familiarity | 1.459x10^-4^ | 1.914x10^-4^ | 0.762 | 0.446 |
| Lexical frequency | -2.005x10^-5^ | 6.132x10^-5^ | -0.327 | 0.744 |
| Manipulability | -1.379x10^-4^ | 8.090x10^-5^ | -1.705 | 0.089 |
| Visual complexity | -1.442x10^-4^ | 1.536x10^-4^ | -0.939 | 0.348 |

The table reports the results of the mixed effects model, with Box-Cox transformed Naming Latencies as dependent variable. The first column reports the name of each term. The second column reports the estimated coefficients (beta) for each term. The third column reported the standard error of the estimate. The fourth column reports the t-value associated with the term. The last column reports the p-values. The model included also a by-subject adjustment to intercept (SD = 0.001505) and a by-item adjustment to intercept (SD = 0.001340) as random effects.

**References**

1. Hutcheson, G. D., & Sofroniou, N. (1999). *The multivariate social scientist: Introductory statistics using generalized linear models*. Sage.
